# Synthesis and Cytotoxic Activity Study of Conjugates of N-Acyl Derivatives of 3,5-Bis(benzylidene)-4-piperidones and Phenothiazine

**DOI:** 10.3390/ijms27094104

**Published:** 2026-05-04

**Authors:** Pavel Yudaev, Yulia Aleksandrova, Inna Shagina, Oleg Artyushin, Elena Sharova, Alexey Rodionov, Margarita Neganova, Valery Brel

**Affiliations:** Nesmeyanov Institute of Organoelement Compounds, Russian Academy of Sciences, Vavilova St., 28, Bld. 1, Moscow 119991, Russiaaleksyu@ineos.ac.ru (Y.A.); schagina.in@yandex.ru (I.S.); oleg.artyushin@gmail.com (O.A.); sharovaev@mail.ru (E.S.); rodalex@ineos.ac.ru (A.R.)

**Keywords:** 3,5-bis(benzylidene)-4-piperidone, phenothiazine, 1,2,3-triazol cycle, conjugate, scaffold, cancer cell, cytotoxicity, hydrochloride

## Abstract

In this study, a simple and efficient method for the synthesis of conjugates of N-acyl derivatives of 3,5-bis(benzylidene)-4-piperidones and phenothiazine was developed. The method was based on the acylation of 3,5-bis(benzylidene)-4-piperidones with chloroacetic acid chloride, followed by treatment of the product with sodium azide and an azide-alkyne [3+2] cycloaddition reaction between the resulting azide and 10-(prop-2-yn-1-yl)-10H-phenothiazine in the final step. Using this method, a series of seven compounds **23**–**29** were synthesized. The structure of synthesized compounds **23**–**29** was studied using ^1^H, ^13^C, and ^19^F NMR spectroscopy and ESI-MS mass spectrometry. The cytotoxicity of compounds **23**–**29** and their hydrochloride salts **30**–**36** towards pancreatic adenocarcinoma Panc-1, bladder cancer T-24, glioblastoma T98G, breast adenocarcinoma BT-20, and normal dermal fibroblast DF-1 cells was studied using an MTT assay. Compound **29**, containing 3,4,5-trimethoxyl radicals at the aromatic ring, and its hydrochloride salt **36**, demonstrated the best cytotoxicity against Panc-1, T-24, T98G, and BT-20 cancer cells. Hydrochloride salts were found to exhibit superior cytotoxicity against Panc-1, T-24, T98G, and BT-20 cancer cells compared to the original 3,5-bis(benzylidene)-4-piperidones and free bases. Selective cytotoxic action against Panc-1, T-24, T98G, and BT-20 cancer cells compared to normal DF-1 cells was also observed for all the obtained compounds and their salts.

## 1. Introduction

Natural products, as well as their structural analogs, are always of interest as a source of effective medicinal products. Interest in them is associated with their high chemical diversity, biochemical specificity, significant molecular activity and pharmacological properties, namely the ability to cause cell cycle arrest, trigger apoptotic death and suppress the growth of malignant cells [1,2]. About 47% of all drugs used in practice were created using natural molecules. These include native compounds, their semi-synthetic analogs, and biomimetics [3]. Curcumin is an example of a natural compound with clinical potential. The wide range of beneficial properties of curcumin, in particular its antioxidant [4], anti-inflammatory [5], neuroprotective [5,6] and antiproliferative [7] properties, has prompted researchers to study its therapeutic efficacy in various diseases. In numerous preclinical studies, curcumin has shown both preventive and potential therapeutic effects in a variety of cancers, including colorectal, liver, pancreatic, prostate, breast, lung, ovarian, and bladder cancers, as well as melanoma and lymphoma [8,9].

Along with curcumin itself, much attention has been drawn to its mono-carbonyl structural analogs, in particular 3,5-bis(benzylidene)-4-piperidones. Thus, it was found that 3,5-bis(2-fluorobenzylidene)-4-piperidone (EF24) is superior to curcumin in pharmacokinetic parameters, possessing increased bioavailability [10,11,12,13].

Almost the entire spectrum of biological activity of curcumin, which includes antitumor, neuroprotective and antioxidant activity and many other properties, is also characteristic of 3,5-bis(benzylidene)-4-piperidones [14,15]. In particular, Das et al. showed that double 3,5-bis(benzylidene)-4-piperidones are highly toxic to human malignant cells (promyelocytic leukemia HL-60 and squamous cell carcinoma HSC-2, HSC-3 and HSC-4) [14]. In turn, Karki et al. demonstrated that 3,5-bis(benzylidene)-4-piperidones are cytotoxic to Molt4/C8 human T-cell leukemia cells, CEM human T-cell lymphoma cells, and L1210 mouse lymphocytic leukemia cells [15].

Furthermore, the structural diversity of 3,5-bis(benzylidene)-4-piperidones and their availability represent a rich source of pharmacophoric molecules for the scaffold-oriented design of hybrid molecular systems with improved antitumor properties, using various pharmacophores with specific biological activity for conjugation. Our research group has previously synthesized hybrid molecular systems based on 3,5-bis(benzylidene)-4-piperidones and secondary plant metabolites such as sesquiterpene lactones (isoalantolactone, alantolactone, dehydrocostus lactone) [16] and terpenoids with a 1,7,7-trimethylbicyclo[2.2.1]heptane framework [17].

In this work, phenothiazine, characterized by the presence of the 10 H-dibenzo[b,e]-1,4-thiazine system, was chosen as a pharmacophore for conjugation. In addition to their primary use as antipsychotic drugs for the treatment of schizophrenia [18,19], phenothiazine-containing compounds have antitumor activity against cervical cancer cells, glioblastoma, and ovarian cancer [20]. The antitumor activity of phenothiazine derivatives is associated with their ability to inhibit carbonic anhydrase IX (an enzyme that supports the survival and proliferation of cancer cells) [21], cause DNA damage and cell cycle arrest, and also cause reactive oxygen species-induced cell death [22,23,24,25]. Some phenothiazine derivatives also induced apoptotic and/or autophagic programmed cell death pathways in various cancer cells, including leukemia, oral cancer, breast cancer, esophageal cancer, and glioblastoma cells [26,27,28,29,30]. For example, thioridazine, a clinically approved antipsychotic, suppresses glioblastoma proliferation by activating AMPK, inducing autophagy, and promoting apoptosis [30]. In addition, phenothiazine derivatives increase the sensitivity of cancer cells to chemotherapy by affecting P-glycoproteins and ATP-dependent membrane pumps, and subsequently increasing the intracellular accumulation of chemotherapeutic drugs [31].

**Figure 1:**
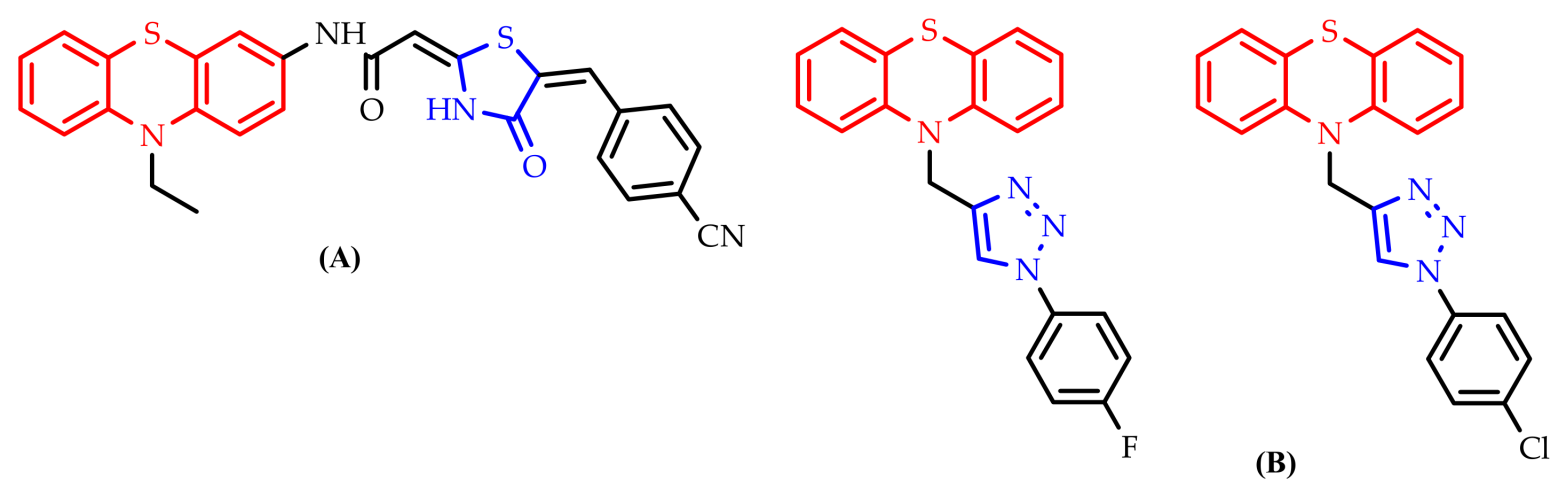
Examples of phenothiazine scaffolds showing anticancer activity against human cancer cell lines: (**A**) prostate and breast cancer cells; (**B**) gastric, esophageal, prostate, breast, and hepatocellular carcinoma cancer cells.

Phenothiazine can also be covalently linked to other pharmacophores (Figure 1), providing new hybrid molecules with increased potency and the potential to overcome the problem of drug resistance in oncology research, reducing dosage and the incidence of adverse reactions [32,33,34]. For example, phenothiazinyl-thiazole hybrids (Figure 1A) exhibited significant cytotoxic activity against PC3 prostate and MCF-7 breast cell lines (IC_50_ less than 10 μM) [35]. Hybrids of phenothiazine and 1,2,3-triazole (Figure 1B) demonstrated a more potent antiproliferative effect against gastric (MGC-803), esophageal (EC-109), prostate (PC-3), breast (MCF-7), and hepatocellular carcinoma (HepG-2) cancer cells (IC_50_ 0.5–9.6 μM) compared to the standard drug, 5-fluorouracil (IC_50_ 10.7–17.6 μM) [36].

Data on the synthesis and biological properties of conjugates of 3,5-bis(benzylidene)-4-piperidone with phenothiazine are absent in the literature, therefore their synthesis and biological screening are an important task in the search for new compounds with improved antitumor activity.

It is known that N-acyl derivatives of 3,5-bis(benzylidene)-4-piperidone containing an amide nitrogen atom in the piperidone cycle have better cytotoxicity compared to the original piperidones. Thus, Dimmock et al. [37] showed that N-acyl derivatives of 3,5-bis(benzylidene)-4-piperidones were significantly more active against murine leukemia cells P388, L1210 and human leukemia T-lymphoblast Molt 4/C8, CEM neoplasms (IC_50_ less than 10 μM) than the original 3,5-bis(benzylidene)-4-piperidones. Therefore, in this work, to compare the antitumor activity, we synthesized conjugates of 3,5-bis(benzylidene)-4-piperidones with amide nitrogen atoms of the piperidone ring.

It should also be noted that the 1,2,3-triazole ring can form various non-covalent interactions, such as hydrogen bonds, van der Waals forces, and dipole–dipole bonds, with various enzymes, proteins, and receptors. These interactions explain its antitumor potential, causing cell cycle arrest and apoptosis in cancer cells [38,39]. For example, in [40] it was found that compounds containing a 1,2,3-triazole ring exhibit binding affinity with the active center of the epidermal growth factor receptor EGFR. The gene encoding EGFR causes uncontrolled growth and division of cancer cells. Also, 1,2,3-triazoles are characterized by low toxicity and excellent pharmacokinetics due to the structure of the five-membered heterocycles and density of the electron clouds. Due to their large dipole moment, 1,2,3-triazoles can act as hydrogen bond donors, mimicking the amide functional group. Triazoles are resistant to metabolic degradation and hydrolysis and are stable under acidic, alkaline and oxidative conditions in living biological systems [41,42]. In this work, it is assumed that the 1,2,3-triazole ring will act not only as a connecting element (linker), but also as an additional pharmacophore.

## 2. Results and Discussion

### 2.1. Chemistry

The aim of this work was to synthesize conjugates of N-acyl derivatives of 3,5-bis(benzylidene)-4-piperidones and phenothiazine derivatives. It has been previously shown that the biological properties of 3,5-bis(benzylidene)-4-piperidones significantly depend on the substituents in the aromatic rings, in particular, their steric and electronic effects dramatically affect cytotoxicity [17,43]. Therefore, as objects of conjugation, we used 3,5-bis(benzylidene)-4-piperidones (**2**–**8**) containing electron-donating, electron-withdrawing, and bulky substituents.

**Scheme 1:**
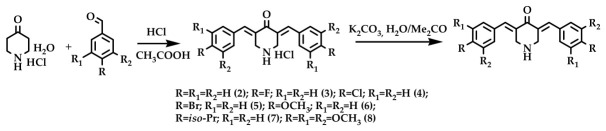
Synthesis of 3,5-bis(benzylidene)-4-piperidones (**2**–**8**).

The synthesis of compounds (**2**–**8**) was carried out according to Scheme 1. The target 3,5-bis(benzylidene)-4-piperidones (**2**–**8**) were obtained by Claisen–Schmidt condensation from 4-piperidone monohydrate hydrochloride and the corresponding aldehyde in the presence of gaseous hydrogen chloride in acetic acid according to the procedure presented in the work [37]. The yield of products **2**–**8** was 65–70%.

The structure of compounds **2**–**8** was determined by ^1^H and ^13^C NMR spectroscopy. Thus, a singlet signal in the 8.2 ppm region indicates the presence of protons in the *sp^2^*-hybridized carbon atom. Signals from protons of the aromatic ring are present in the range of 8.1–7.4 ppm, and signals from methylene protons of the piperidone ring are located in the range of 4.5 ppm. ^13^C NMR spectra confirm the structure of compounds **2**–**8**. For example, for compound **2**, a signal in the range of 188.02 ppm is present in the spectra, belonging to the C=O group. The signals of atoms in the range of 134.98 ppm (s, C=) and 136.02 ppm (s, CH=) belong to carbon atoms of the double bond. The signals of aromatic carbon atoms are located at 128–135 ppm. A singlet with a chemical shift of 46.71 ppm indicates the presence of an NCH_2_ fragment in the molecule. The spectral data for compounds **2**–**6** correspond to the literature data [37].

**Scheme 2:**
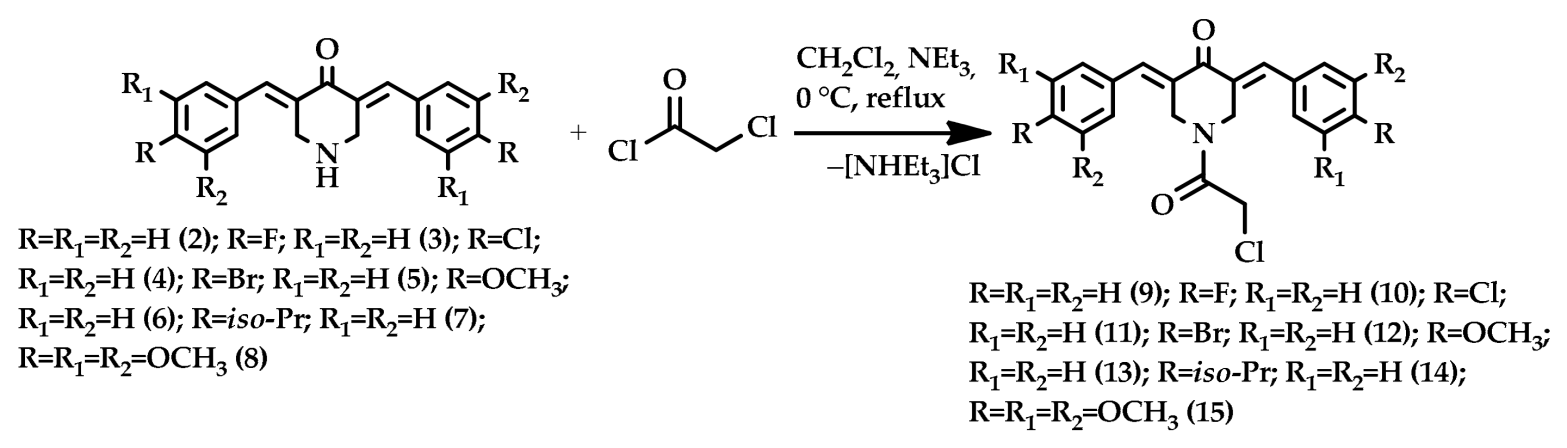
Synthesis of *3,5-bis(benzylidene)-1-(2-chloroacetyl)piperidin-4-ones* (**9**–**15**).

In the next step, to obtain N-acylated derivatives, 3,5-bis(benzylidene)-4-piperidones **2**–**8** were condensed using chloroacetyl chloride (Scheme 2). The acylation reaction was carried out in dichloromethane in the presence of triethylamine as a base according to the previously described procedure [44,45]. Since the acylation reaction is exothermic, a solution of 3,5-bis(benzylidene)-4-piperidones **2**–**8** and triethylamine in dichloromethane was first cooled to 0 °C. Then, chloroacetyl chloride was added dropwise with stirring to a solution of 3,5-bis(benzylidene)-4-piperidones **2**–**8** and triethylamine in dichloromethane (molar ratio of piperidone: chloroacetyl chloride 1:1.5). The reaction was then carried out with the solvent boiling. The reaction progress was monitored by thin-layer chromatography (eluent: CHCl_3_:CH_3_OH/10:0.3). The yield of target compounds **9**–**15** was 74–80%.

**Scheme 3:**
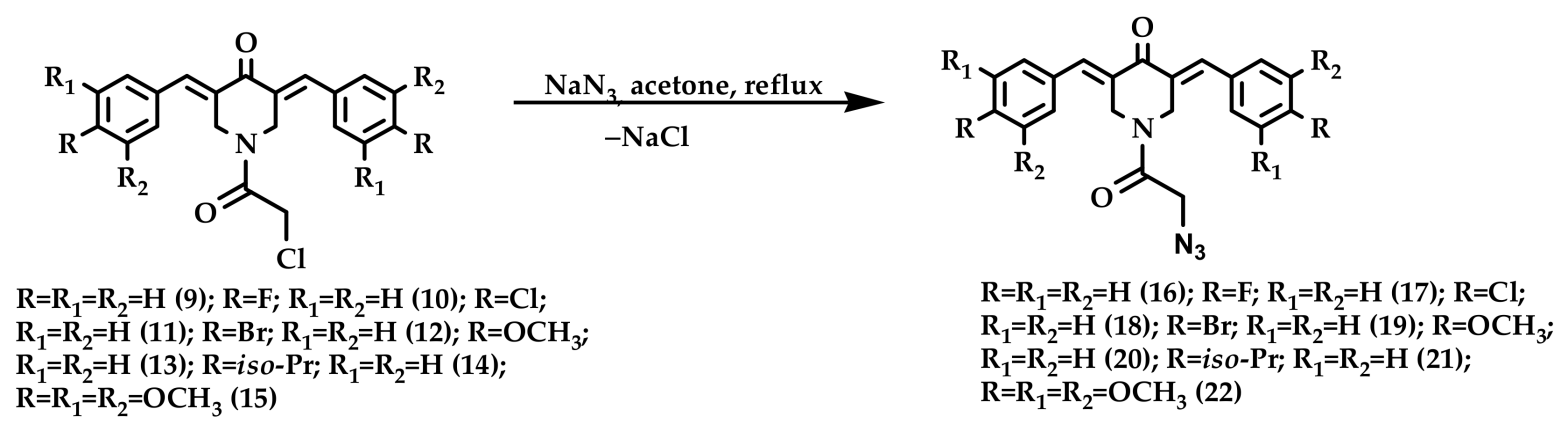
Synthesis of *1-(2-azidoacetyl)-3,5-bis(benzylidene)-piperidin-4-ones* (**16**–**22**).

In the third step, 3,5-bis(benzylidene)-1-(2-chloroacetyl)piperidin-4-ones **9**–**15** were treated with sodium azide (piperidone/sodium azide molar ratio 1/3) in boiling acetone or acetonitrile (Scheme 3). The reaction progress was monitored using thin-layer chromatography (CHCl_3_:CH_3_OH/10:0.08, *v*/*v*). The target azides **16**–**22** were isolated by column chromatography (eluent: CHCl_3_:CH_3_OH/100:0.8, *v*/*v*). The resulting azides **16**–**22** were crystalline substances that decomposed with the release of nitrogen. The structures of the obtained compounds **16**–**22** were confirmed by spectral methods (^1^H, ^13^C, ^19^F NMR spectroscopy, ESI-MS mass spectrometry and IR spectroscopy, Supplementary Materials, Figures S1–S22). In the ^1^H NMR spectra of azides **16**–**22**, a shift in the signals of the C(O)CH_2_ group protons to a strong field was observed compared to the starting piperidones **2**–**8**. The IR spectra of the obtained compounds **16**–**22** showed characteristic bands of the stretching vibrations of the azide group (e.g., for azide **16**, in the region of 2108 cm^–1^).

**Scheme 4:**
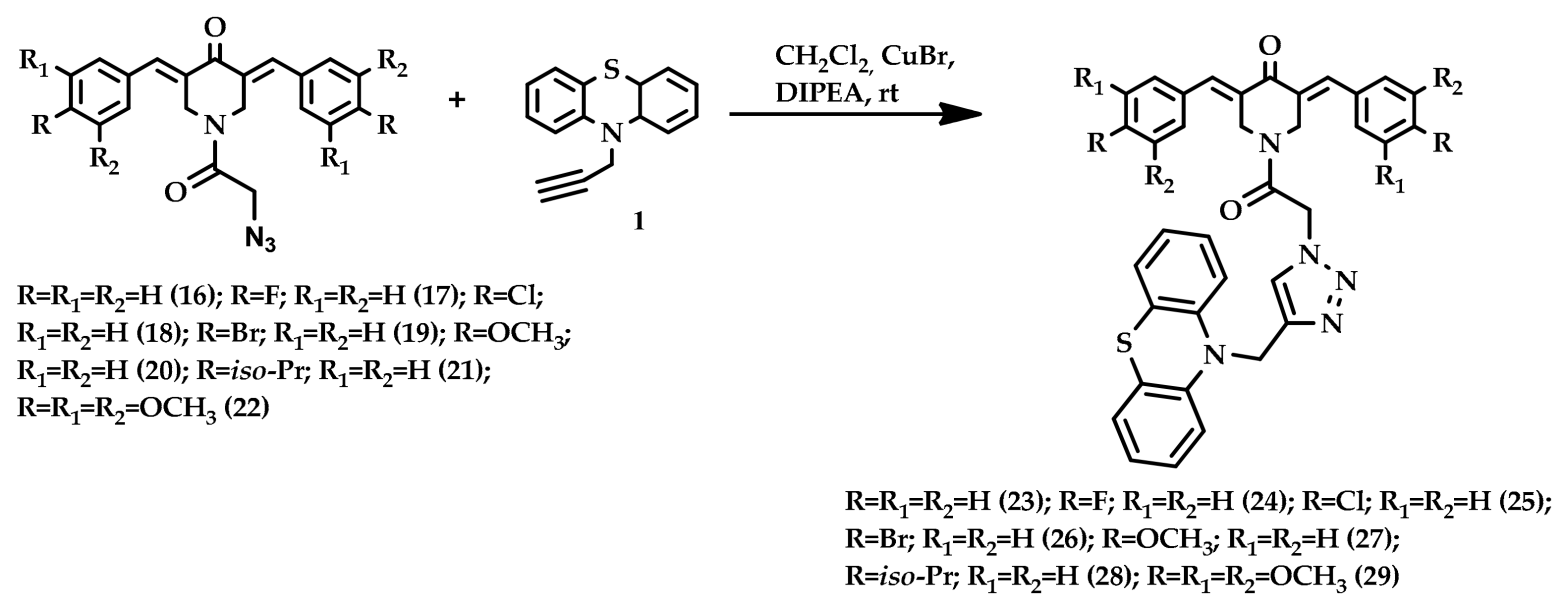
Synthesis of *(3E,5E)-1-(2-(4-((10H-phenothiazine-10-yl)methyl)-1H-1,2,3-triazol-1-yl)acetyl)-3,5-bis(benzilydene)-piperidin-4-ones* (**23**–**29**).

The method chosen for synthesizing conjugates containing the 1,2,3-triazole ring was the “click” chemistry method, which consists of the azide-alkyne cycloaddition reaction of 1-(2-azidoacetyl)-3,5-bis(benzylidene)-piperidin-4-one **16**–**22** and 10-(prop-2-yn-1-yl)-10H-phenothiazine **1**, previously synthesized according to a known method [43], in a methylene chloride medium in the presence of catalytic amounts of 5 mol.% CuBr and 10 mol.% DIPEA (Scheme 4)**.** The reaction was carried out at room temperature. The reaction progress was monitored by TLC (CHCl_3_:CH_3_OH/10:0.15, *v*/*v*). The target conjugates **16**–**22** were isolated in pure form by column chromatography (eluent: CHCl_3_:CH_3_OH/100:1.5) in moderate yields (30–40%). The choice of this method is due to the greater nucleophilicity of the nitrogen atom of the piperidone ring compared to the nitrogen atom of phenothiazine and the possibility of carrying out acylation at the nitrogen atom under milder conditions.

The structure of conjugates **23**–**29** was confirmed by NMR (^1^H, ^13^C, ^19^F) and ESI-MS mass spectrometry (see Supplementary Materials, Figures S23–S43). The proton and carbon signals in the ^1^H and ^13^C NMR spectra of compounds **23**–**29** confirm the formation of a 1,4-disubstituted 1,2,3-triazole ring with a *3E,5E* configuration of the double bonds in the 3,5-bis(benzylidene)piperidin-4-one moiety.

For compound **23**, the signals of the methylene CH_2_ groups of phenothiazine and the CH_2_ group bound to the carbonyl C(O) at the nitrogen atom of the piperidone ring are present in the ^1^H NMR spectrum in the region of 4.98 and 5.19 ppm, respectively. In turn, the signals of the protons of the phenothiazine skeleton are in the region of 6.75–7.11 ppm. The singlet signal of the proton of the 1,2,3-triazole ring in the region of 7.45 ppm was clearly distinguishable in the ^1^H NMR spectrum of compound **29**, which contains a 3,4,5-trimethoxyl radical at the aromatic ring.

It should also be noted that the obtained compounds **16**–**22** and **23**–**29** represent two isomers (rotamers) in deuterochloroform solution. The formation of isomers corresponds to the literature data for amides [46,47]. The presence of two isomers is indicated by the doubling of the signals of the protons and carbons of the methylene groups of the N-CH_2_ piperidin-4-onium ring due to their magnetic non-equivalence in the ^1^H and ^13^C NMR spectra of compounds **16**–**22** and **23**–**29**, as well as the presence of two singlet signals in the ^19^F NMR spectrum of compounds **17** and **24** (see Supplementary Materials).

**Scheme 5:**
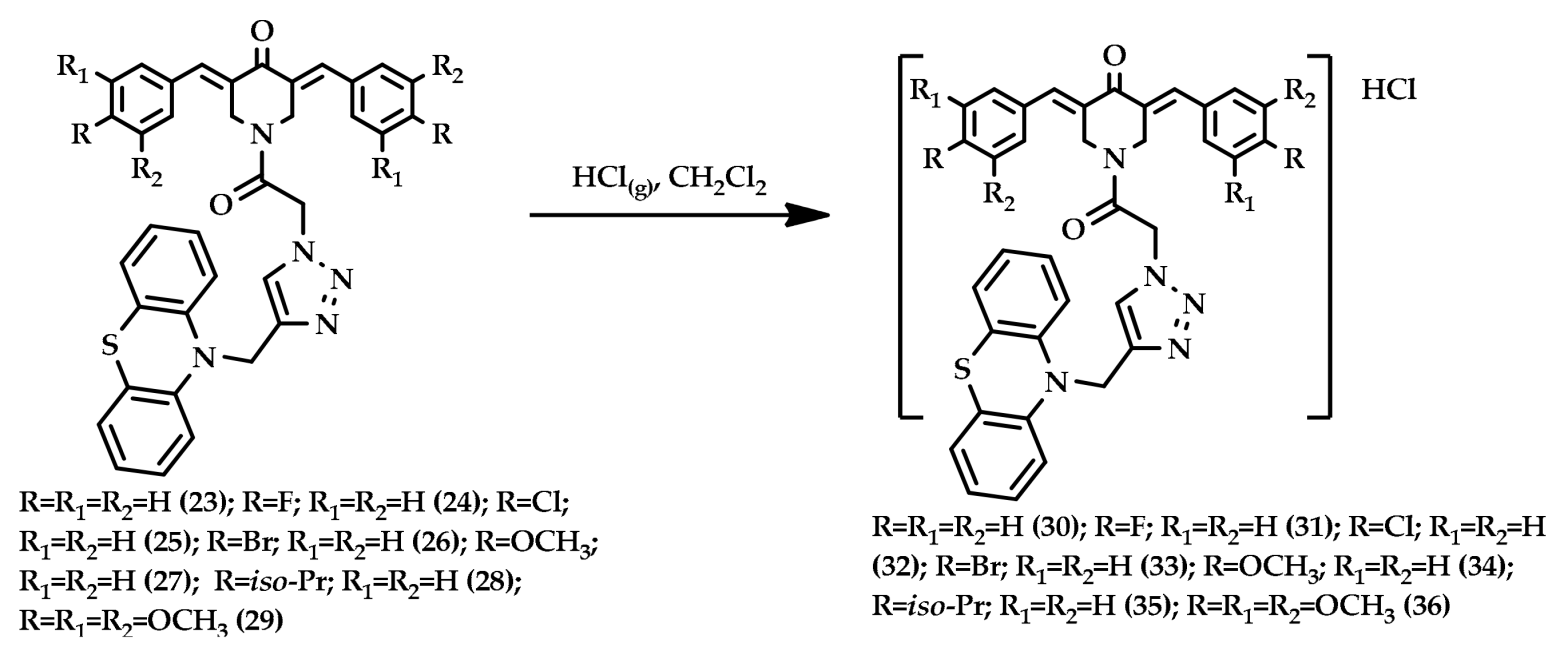
Synthesis of *(3E,5E)-1-(2-(4-((10H-phenothiazine-10-yl)methyl)-1H-1,2,3-triazol-1-yl)acetyl)-3,5-bis(benzylidene)-piperidin-4-ones hydrochloride* (**30**–**36**).

In addition to the obtained conjugates **23**–**29**, we decided to synthesize their hydrochloride salts. Salts **30**–**36** were obtained by bubbling hydrogen chloride through a solution of compounds **23**–**29** in dichloromethane, followed by removal of the solvent (Scheme 5). We confirmed the preservation of the conjugate structure after treatment with hydrogen chloride using ^1^H and ^13^C NMR spectroscopy (Figures S44–S53, Supplementary Materials). ESI-MS was not used to study the structure of salts **30**–**36**. Changes in the chemical shifts in the carbon atom signals in the ^13^C NMR spectrum may indicate the formation of salts. For example, for compound **29** and salt **36**, the signal for the carbon of the methylene group at phenothiazine is observed at 50.86 and 54.18 ppm (a difference of 3.32 ppm), the carbon of the methylene group at carbonyl C(O) at 46.52 and 52.23 (a difference of 5.71 ppm), and the carbon of the methylene group of the piperidone ring at 44.93 and 51.73 (a difference of 6.8 ppm). For further biological screening, salts **30**–**36** were used without additional purification.

Thus, we have developed a method for synthesizing a series of new hybrid molecules containing two pharmacophores: 3,5-bis(benzylidene)-4-piperidone and a phenothiazine moiety.

### 2.2. Biological Evaluation

In the first stage of the study, we assessed the cytotoxic properties of the parent piperidones **2**–**8** and their phenothiazine conjugates **23**–**29** against the pancreatic adenocarcinoma cell line Panc-1.

Curcumin was selected as a positive control for the cytotoxic activity of the synthesized 3,5-bis(arylidene)-4-piperidones—a natural biphenylylhexanoid serving as a structural prototype for the tested compounds due to the presence of conjugated α,β-unsaturated carbonyl fragments and aromatic substituents, with a well-documented anti-tumor effect across panels of cancer cell lines. This choice ensures validation of result reproducibility in the employed cellular models (positive correlation with literature data) and enables quantitative extrapolation of the advantages of chemical modifications of piperidones relative to the natural analog.

As shown in Table 1, the vast majority of the synthesized hybrid molecules exhibited more pronounced cytotoxicity than the parent piperidones. Specifically, the IC_50_ value (the concentration inducing a 50% reduction in cell viability) for the conjugates was achieved at concentrations 2–3 times lower than those for the piperidones.

Therefore, it was advisable to conduct further, expanded studies of the cytotoxic effect specifically for the conjugates that demonstrated the most pronounced activity.

The cytotoxic profile of the synthesized compounds was evaluated on a panel of human cancer cell lines representing aggressive and treatment-resistant malignancies: bladder cancer (T-24), glioblastoma (T98G), and breast cancer (BT-20). A human dermal fibroblast cell line (DF-1) was used to assess potential selectivity. The results, expressed as half-maximal inhibitory concentrations (IC_50_) after 72 h of exposure to the test compounds, are presented in Table 2.

Using a structure-activity relationship, we found that the nature of the substituents in the 3,5-bis(benzylidene) fragment of the piperidone ring critically determines the degree of cytotoxic action of the molecules. Thus, conjugates containing a bromine atom (**26**, **33**), single methoxy group (**27**, **34**), and an isopropyl group (**28**, **35**) were completely inactive (IC_50_ > 30 μM) at the maximum concentration used against all cell lines tested. Apparently, this may be due to several factors: (1) electronic effects—Br, being a weakly electron-withdrawing halogen, provides weak double bond acceptability compared to Cl/F; the single methoxy group provides an insufficient donor effect, thereby insufficiently activating the bis-benzylidene fragment for nucleophilic interaction with protein thiols, which is often considered as the mechanism of the cytotoxic action of piperidones; (2) steric hindrance—the isopropyl group and Br, being large fragments, can hinder the conformation of the molecule and its access to the biotarget; and (3) reduced solubility in aqueous media due to increased lipophilicity and, as a consequence, reduced solubility in aqueous media of cell cultures, hindering penetration through membranes in contrast to the more polar Cl/F and methoxy groups.

A noteworthy result was the observed pronounced cytotoxic effect of hydrochloride salts (compounds **30**–**36**) not only in comparison with the parent piperidones **2**–**8**, but also with their main analogs (compounds **23**–**29**) and curcumin. Thus, compound **36** demonstrated a 2.8–4.6-fold increase in activity against cancer cell lines compared with **29**. This effect is likely due to the improved physicochemical properties of these molecules. In particular, protonation has made it possible to increase the solubility of a number of already known molecules [48,49] and improve cellular uptake through passive diffusion of an ion pair or active transport mechanisms [50]. In addition, a positive charge can enhance electrostatic interactions with negatively charged phospholipid membranes or biological targets, such as DNA or other specific enzymes.

Furthermore, we noted that none of the studied compounds at the maximum tested concentration of 30 μM significantly affected the viability of normal DF-1 cells (the proportion of viable cells exceeded 50%, and the selectivity index reached >50.8 for **36** on the T-24 cell line), suggesting some selectivity for tumor-derived cells. Thus, the obtained compounds are valuable for the development of antitumor agents with reduced toxicity to normal tissues.

### 2.3. Docking

**Figure 2:**
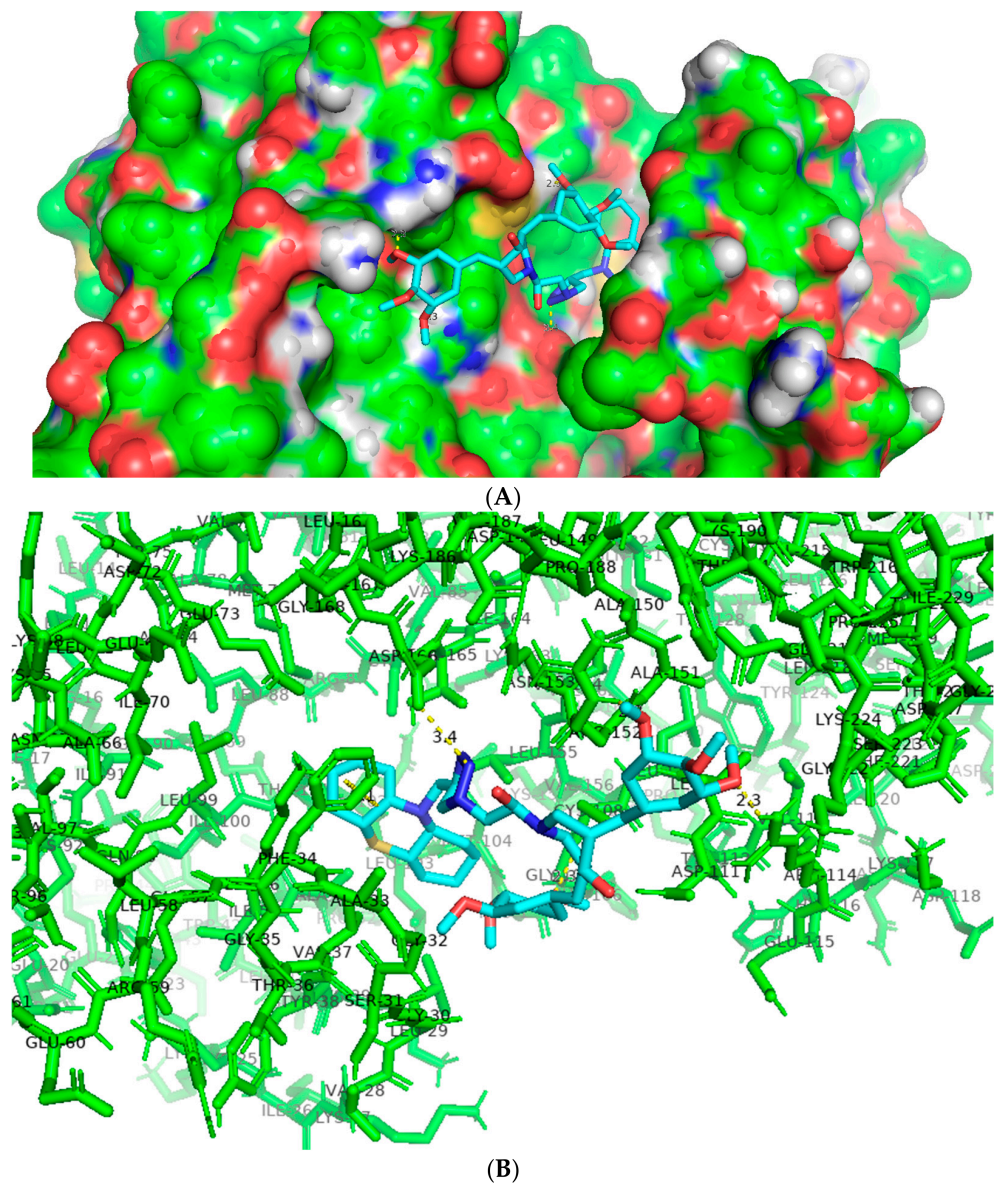
Location of the ligand **29** in the active site of the EGFR (1M14) receptor: (**A**) Surface representation; (**B**) ribbon structure.

Molecular docking is a computational technique used to predict the most likely binding pose of a ligand to a protein. It is a powerful tool for drug discovery and structural biology. In this study, docking analysis was performed to evaluate the binding behavior of the most bioactive compound (**29**) among the synthesized amides within the ATP-binding cleft of EGFR tyrosine kinase (PDB ID: 1M14) (Figure 2A,B), a well-established target in cancer therapeutics. As in [40], we used erlatinib as a reference substance. Compound **29** demonstrated a notable binding free energy of −7.4 kcal/mol, which is comparable to that of the reference inhibitor erlotinib (−6.7 kcal/mol). These data suggest that compound **29** has a strong affinity for the EGFR active site and may act as a competitive kinase inhibitor.

In conclusion, the docking results indicate that compound **29** exhibits a strong binding affinity to EGFR in the binding pocket of the clinically used inhibitor erlotinib and demonstrated promising anticancer activity in vitro.

## 3. Materials and Methods

### 3.1. Materials

All commercial reagents were used as purchased without further purification; all solvents used in the reactions were freshly distilled from appropriate drying agents before use. Phenothiazine (98%) was purchased from ABCR GmbH (Karlsruhe, Germany). 4-Piperidone monohydrate hydrochloride (98%), propargyl bromide (80% in toluene), d-chloroform (≥99.8%), dimethyl sulfoxide-d_6_ (≥99.8%), and N,N-diisopropylethylamine (DIPEA, 99.5%) were obtained from Sigma-Aldrich (Saint Louis, MO, USA). Chloroacetyl chloride (98%) was purchased from Acmec (Shanghai Acmec Biochemical Technology Co., Ltd., Fengxian, Shanghai, China). Methanol (≥99.5%) was purchased from Vekton (St. Petersburg, Russia). Chloroform (≥99.85%), dichloromethane (≥99.0%), acetone (≥99.75%), acetonitrile (≥99.7%), triethylamine (≥99.5%), ethyl acetate (≥99%), n-hexane (≥97%), dimethylformamide (DMF, ≥99.95%), hydrochloric acid (35–38%), acetic acid (99.8%), and potassium carbonate (>98%) were purchased from Component-Reactive (Moscow, Russia). Copper (I) bromide (99.9%) was purchased from Ecotec, Ltd. (Moscow, Russia). Sodium sulfate (95%) was purchased from Chemistry 21 century (Moscow, Russia). Dimethyl sulfoxide (DMSO, ≥99.9%) was purchased from WuHan ServiceBio Technology Co., Ltd. (Wuhan, Hubei, China). Sodium azide (99%) was purchased from Ruskhim (Moscow, Russia). Potassium tert-butoxide (99.99%) was purchased from Merck, Ltd. (Darmstadt, Germany).

Analytical TLC was performed on Merck silica gel 60 F_254_ plates (Merck, Darmstadt, Germany), visualized under UV light (λ_max_ = 254 nm) or by staining with potassium permanganate. Column chromatography was carried out using Merck silica gel (Kieselgel 60, 0.063–0.200 mm, Darmstadt, Germany).

### 3.2. Synthesis of 10-(Prop-2-yn-1-yl)-10H-phenothiazine (***1***)

Compound **1** was synthesized according to the literature procedure [51]. A mixture of DMF (2 mL) with phenothiazine (1 mmoL), potassium tertiary butoxide (1.5 mmoL) and propargyl bromide (1.2 mmoL) was stirred for 4 h. Upon completion of the reaction, as controlled by TLC (n-hexane: ethyl acetate, *v*/*v*, 9:1), the mixture was extracted twice by 5 mL of ice-cold water–diethyl ether and the combined organic layers were dried by anhydrous Na_2_SO_4_. The organic solvent was rotary-evaporated, and the crude product was subjected to column chromatography (n-hexane: ethyl acetate, *v*/*v*, 9:1) to afford pure compound **1** as yellowish-brown solid. Yield: 94%. ^1^H NMR spectrum, δ, ppm: 2.74 (1H, s, CH), 4.58 (2H, s, CH_2_), 6.735–6.75 (2H, m, Ar-H), 6.97–7.09 (4H, m, Ar-H), 7.28–7.31 (2H, m, Ar-H). ^13^C NMR spectrum, δ, ppm: 144.18, 129.05, 128.05, 127.43, 124.09, 121.31, 116.12, 98.57, 74.08, 45.89.


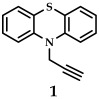


### 3.3. General Procedure of 3,5-Bis(benzylidene)-4-piperidone (***2***–***8***) Synthesis

Compounds **2**–**8** were synthesized according to the literature procedure with some modifications [37]. The appropriate aryl aldehyde (26.71 mmol) was added to a suspension of 4-piperidone monohydrate hydrochloride (13.03 mmol) in glacial acetic acid (35 mL). Hydrogen chloride (prepared from sodium chloride and sulfuric acid) was passed through this mixture for 0.5 h, during which time a clear solution was obtained. After stirring at room temperature for 24 h, the precipitate was collected and added to a mixture of a saturated aqueous potassium carbonate solution (25% *w*/*v*, 25 mL) and acetone (25 mL); the resultant mixture was stirred for 0.5 h. The free base was collected, washed with water (50 mL), and dried. The compounds were recrystallized from 95% ethanol. Yields are given for compounds isolated without further purification.

*3,5-bis(benzylidene)-4-piperidone (***2***).* The yield of non-recrystallized compound **2** was 80%. ^1^H NMR, δ: 4.17 (s, 4H, 2CH_2_); 7.38–7.43 (m, 10H, C_6_H_5_); 7.83 (s, 2H). ^13^C NMR, δ: 46.71 (s, 2CH_2_), 128.59, 129.11, 130.54, 135.2 (s, 12C, Ph), 134.98 (s, C=), 136.02 (s, CH=), 188.02 (s, C=O) [17].

*3,5-bis(4-fluorobenzylidene)-4-piperidone (***3***).* The yield of non-recrystallized compound **3** was 79%. ^1^H NMR, δ: 4.15 (s, 4H, 2CH_2_,); 7.10–7.16 (m, 4H, C_6_H_4_F); 7.37–7.42 (m, 4H, C_6_H_4_F); 7.88 (s, 2H). ^13^C NMR, δ: 46.62 (s, 2CH_2_), 115.79 (d, J_C–F_ = 40 Hz, Ar), 131.35 (s, C=),132.46 (d, J_C–F_ = 5 Hz, Ar), 134.54 (s, CH=), 134.92 (s, Ar), 163.45 (d, J_C–F_ = 460 Hz, Ar), 187.69 (s, C=O). ^19^F NMR, δ: 110. 70 [17].

3,5-bis(4-chlorobenzylidene)-4-piperidone (**4**). The yield of non-recrystallized compound **4** was 79%. ^1^H NMR, δ: 4.13 (s, 4H, 2CH_2_); 7.31–7.42 (m, 8H, C_6_H_5_); 7.75 (s, 2H). ^13^C NMR, δ: 48.15 (s, 2CH_2_), 129.05, 132.48, 132.76, 134.36 (s, 12C, Ar), 134.41 (s, C=), 137.06 (s, CH=), 187.57 (s, C=O) [17].

3,5-bis(4-bromobenzylidene)-4-piperidone (**5**). ^1^H NMR, δ: 4.13 (s, 4H, 2CH_2_); 7.25–7.58 (m, 8H, C_6_H_5_); 7.73 (s, 2H).

3,5-bis(4-methoxybenzylidene)-4-piperidone (**6**). The yield of non-recrystallized compound **6** was 76%. ^1^H NMR, δ: 3.87 (s, 6H, 2 OCH_3_); 4.17 (s, 4H, 2CH_2_); 6.95–6.97 (m, 4 H, Ar); 7.37–7.39 (m, 4H, Ar); 7.78 (s, 2H). ^13^C NMR, δ: 48.21 (s, 2CH_2_), 55.39 (s, 2OCH_3_); 113.04, 127.98, 132.45, 133.15 (s, 12C, Ar), 135.63 (s, C=), 160.31 (s, CH=), 187.93 (s, C=O) [17].

3,5-bis(4-iso-propylbenzylidene)-4-piperidone (**7**). The yield of non-recrystallized compound **7** was 75%. ^1^H NMR, δ: 1.30 (d, 12H, J = 6.0 Hz); 2.96 (six, 2H, J = 6.0 Hz); 4.18 (s, 4H, 2CH_2_); 7.28–7.37 (m, 8H, Ar); 7.81 (s, 2H). ^13^C NMR, δ: 23.83 (s, 2CH_3_), 34.06 (s, 2CH); 48.20 (s, 2CH_2_), 126.71, 130.78, 132.82, 134.31 (s, 12C, Ar), 135.97 (s, C=), 150.28 (s, CH=), 188.07 (s, C=O) [17].

3,5-bis(3,4,5-trimethoxybenzylidene)-4-piperidone (**8**). The yield of non-recrystallized compound **8** was 77%. ^1^H NMR, δ: 3.88, 3.89 (s, 18H, 9 OCH_3_); 4.19 (s, 4H, 2CH_2_); 6.61 (s, 4H, Ar); 7.72 (s, 2H). ^13^C NMR, δ: 23.83 (s, 2CH_3_), 34.06 (s, 2CH); 48.20 (s, 2CH_2_), 107.90, 130.65, 134.18, 136.17 (s, 12C, Ar), 139.10 (s, C=), 153.07 (s, CH=), 187.53 (s, C=O) [17].

### 3.4. General Procedure of (3E,5E)-3,5-Bis(benzylidene)-1-(2-chloroacetyl)piperidin-4-one (***9***–***15***) Synthesis

Compounds **9**–**15** were synthesized according to the literature procedure [43,44,52]. A mixture of **2**–**8** (0.65 mmoL) and triethylamine (0.975 mmoL) in dichloromethane was maintained at 0 °C (ice bath). To this cooled mixture, chloroacetyl chloride (0.975 mmoL) was added dropwise. After the complete addition of chloroacetyl chloride, the reaction mixture was stirred at reflux. The reaction progress was controlled by TLC (chloroform:methanol 10:0.3, *v*/*v*). After completion of the reaction, the solvent was evaporated and the residue thus obtained was washed with water, filtered and dried. The products obtained were pure enough to be used for the subsequent step (74–80% yield).

*(3E,5E)-3,5-bis(benzylidene)-1-(2-chloroacetyl)piperidin-4-one (***9***).*^1^H NMR (DMSO-d_6_) δ (ppm): 4.35 (s, 2H, CH_2_Cl), 4.86 (s, 2H, piperidinyl NCH_2_), 4.89 (s, 2H, piperidinyl NCH_2_), 7.47–7.75 (m, 12H, 10 arom. H + 2 olefinic CH). ^13^C NMR (DMSO-d_6_) δ (ppm): 41.6 (CH_2_Cl), 42.6, 46.7 (piperidinyl NCH_2_), 128.8, 129.6, 130.4, 130.7, 131.9, 134.2, 136.4, 136.5 (arom. C + olefinic C), 165.0 (acyl CO), 185.8 (piperidinyl CO) [52].

*(3E,5E)-3,5-bis(4-fluorobenzylidene)-1-(2-chloroacetyl)piperidin-4-one (***10***).* ^1^H NMR (500 MHz, DMSO-d_6_) δ (ppm): 4.37 (s, 2H, CH_2_Cl), 4.84 (s, 2H, piperidinyl NCH_2_), 4.86 (s, 2H, piperidinyl NCH_2_), 7.35 (t, J = 8.7 Hz, 4H, arom. H), 7.64–7.72 (m, 6H, 4 arom. H + 2 olefinic CH). ^13^C-NMR (125 MHz, DMSO-d_6_) δ (ppm): 41.5 (CH_2_Cl), 42.5, 46.5 (piperidinyl NCH_2_), 115.7, 115.9, 130.71, 130.73, 131.6, 131.7, 132.8, 133.0, 135.2, 135.4, 161.5, 163.5 (arom. C + olefinic C), 165.0 (acyl CO), 185.6 (piperidinyl CO) [52].

*(3E,5E)-3,5-bis(4-chlorobenzylidene)-1-(2-chloroacetyl)piperidin-4-one (***11***).* 1H NMR (DMSO-d_6_) δ (ppm): 4.36 (s, 2H, CH_2_Cl), 4.83 (s, 2H, piperidinyl NCH_2_), 4.85 (s, 2H, piperidinyl NCH_2_), 7.56–7.70 (m, 10H, 8 arom. H + 2 olefinic CH). ^13^C NMR (DMSO-d_6_) δ (ppm): 41.5 (CH_2_Cl + piperidinyl NCH_2_), 128.8, 132.4, 133.0, 134.3 (arom. C + olefinic C), 165.0 (acyl CO), 185.5 (piperidinyl CO) [52].

*(3E,5E)-3,5-bis(4-bromobenzylidene)-1-(2-chloroacetyl)piperidin-4-one (* **12** *).*

^1^H NMR (500 MHz, DMSO-d_6_) δ (ppm): 4.36 (s, 2H, CH_2_Cl), 4.80 (s, 2H, 2 piperidinyl NCH_2_), 4.84 (s, 2H, 2 piperidinyl NCH_2_), 7.51–7.52 (m, 4H, arom. H), 7.63–7.71 (m, 6H, 4 arom. H + 2 olefinic CH). ^13^C NMR (125 MHz, DMSO-d_6_) δ (ppm): 41.6 (CH_2_Cl + piperidinyl NCH_2_), 123.2, 131.8, 132.6, 133.4 (arom. C + olefinic C), 165.1 (acyl CO), 185.6 (piperidinyl CO) [52].

*(3E,5E)-3,5-bis(4-methoxybenzylidene)-1-(2-chloroacetyl)piperidin-4-one (***13***).* ^1^H NMR (500 MHz, CDCl_3_) δ (ppm): 3.82 (s, 6H), 3.92 (s, 2H), 4.75 (s, 2H), 4.88 (s, 2H), 6.93 (d, 2H), 6.96 (d, 2H), 7.33 (d, 2H), 7.41 (d, 2H), 7,77 (s, 1H), 7.80 (s, 1H). ^13^C NMR (125 MHz, CDCl_3_) δ (ppm): 40.9, 44.1, 47.0, 55.4, 114.4, 114.5, 127.0, 127.3, 128.9, 129.1, 132.2, 132.7, 137.4, 138.4, 160.9, 165.2, 185.8 [45].

*(3E,5E)-3,5-bis(4-isopropylbenzylidene)-1-(2-chloroacetyl)piperidin-4-one (***14***).* ^1^H NMR (300 MHz, CDCl_3_) δ (ppm): 1.24 (s, 6H, CH_3_ isopropyl), 2.91 (m, 2H, CH isopropyl), 3.94 (s, 2H, CH_2_Cl), 4.78 (s, 2H, 2 piperidinyl NCH_2_), 4.89 (s, 2H, 2 piperidinyl NCH_2_), 7.25–7.35 (m, 8H, arom. H), 7.79 (s, 1H), 7.81 (s, 1H).

*(3E,5E)-3,5-bis(3,4,5-trimethoxybenzylidene)-1-(2-chloroacetyl)piperidin-4-one (***15***).* ^1^H NMR (400 MHz, CDCl_3_): δ 3.91 (s, 18H), 3.97 (s, 2H), 4.89 (s, 2H), 4.94 (s, 2H), 6.64 (s, 2H), 6.72 (s, 2H), 7.78 (s, 1H), 7.81 (s, 1H) [44].

### 3.5. General Procedure of (3E,5E)-1-(2-Azidoacetyl)-3,5-bis(benzylidene)-piperidin-4-ones (***16***–***22***) Synthesis

The sodium azide (10.86 mmoL) was added to the solution of compounds **9***–***15** (3.62 mmoL) in acetone or acetonitrile (15 mL). The reaction was carried out with stirring at reflux. Reaction progress was controlled by TLC (chloroform: methanol 10:0.08, *v*/*v*). The solution was separated from the precipitate. Acetonitrile was removed under vacuum. Azides were obtained in quantitative yield. The products were purified by column chromatography (eluent: chloroform/methanol 100:0.8, *v*/*v*).

*(3E,5E)-1-(2-azidoacetyl)-3,5-bis(* *benzylidene)-piperidin-4-one (* **16** *)*


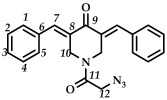


M.p. > 165 °C (decomposition with release of N_2_ gas). ^1^H NMR (300 MHz, CDCl_3_) δ (ppm): 7.86, 7.89 (2H, s, H-7), 7.37–7.45 (10H, m, H-1, H-2, H-3, H-4, H-5), 4.62, 4.94 (4H, s, H-10), 3.73 (2H, s, H-12). ^13^C NMR (100 MHz, CDCl_3_) δ (ppm): 185.80 (C9), 166.06 (C11), 138.98 (C7), 137.90 (C8), 134.10–134.39 (C6), 130.97, 130.68, 130.14, 129–83-129.92 (C3), 128.88–129.03, 49.51 (C12), 43.72–45.45 (C10). IR (KBr, ν/cm^−1^): 2108 (N_3_), 1672, 1655, 1606 and 1571 (C=O), 1491, 1468, 1275, 1232, 1175, 986, 779, 701,691, 639, 514. HRMS (ESI+) of C_21_H_18_N_4_O_2_, *m*/*z*: calcd for [M+H]^+^ 359.1503, found 359.1506.

*(3E,5E)-1-(2-azidoacetyl)-3,5-bis(4-fluorobenzylidene)-piperidin-4-one (* **17** *)*


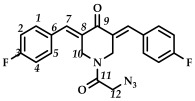


M.p. ˃ 80°C (decomposition with release of N_2_ gas). ^1^H NMR (300 MHz, CDCl_3_) δ (ppm): 7.72, 7.75 (2H, s, H-7), 7.08–7.39 (8H, m, H-1, H-2, H-4, H-5), 4.62, 4.87 (4H, s, H-10), 3.83 (2H, s, H-12). ^13^C NMR (100 MHz, CDCl_3_) δ (ppm): 185.52 (C9), 166.12 (C11), 164.49, 161.98, 137.51 (C7), 136.62 (C8), 132.35–132.76 (C6), 130.53, 130.23, 116.12–116.26, 45.58 (C12), 43.63–45.58 (C10). ^19^F NMR, δ (ppm): −108.98, −109.31. IR (KBr, ν/cm^−1^):2108 (N_3_), 1661, 1615, 1600 and 1508 (C=O), 1455, 1273, 1230, 1159, 1099, 990, 836, 529. HRMS (ESI+) of C_21_H_16_F_2_N_4_O_2_, *m*/*z*: calcd for [M+H]^+^395.1314, found 395.1319.

*(3E,5E)-1-(2-azidoacetyl)-3,5-bis(4-chlorobenzylidene)-piperidin-4-one (* **18** *)*


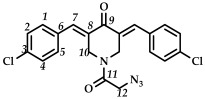


M.p. ˃ 157. °C (decomposition with release of N_2_ gas). ^1^H NMR (300 MHz, CDCl_3_) δ (ppm): 7.79, 7.83 (2H, s, H-7), 7.29–7.45 (8H, m, H-1, H-2, H-4, H-5), 4.62, 4.91 (4H, s, H-10), 3.78 (2H, s, H-12). ^13^C NMR (100 MHz, CDCl_3_) δ (ppm): 185.62 (C9), 166.06 (C11), 137.90 (C7), 136.89 (C8), 136.17–136.79, 132.92–133.12 (C6), 132.18–132.34, 132.01, 131.23–131.54, 124.53, 50.56 (C12), 43.80–45.69 (C10). IR (KBr, ν/cm^−1^): 2102 (N_3_), 1675, 1657, 1612 and 1587 (C=O), 1492, 1464, 1407, 1274, 1260, 1232, 1172,1096, 1013, 990, 839, 522. HRMS (ESI+) of C_21_H_16_Cl_2_N_4_O_2_, *m*/*z*: calcd for [M+H]^+^427.0723, found 427.0725.

*(3E,5E)-1-(2-azidoacetyl)-3,5-bis(4-bromobenzylidene)-piperidin-4-one (* **19** *)*


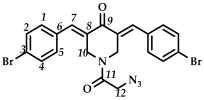


M.p. ˃ 168. °C (decomposition with release of N_2_ gas). ^1^H NMR (300 MHz, CDCl_3_) δ (ppm): 7.80, 7.84 (2H, s, H-7), 7.26–7.63 (8H, m, H-1, H-2, H-4, H-5), 4.62, 4.92 (4H, s, H-10), 3.77 (2H, s, H-12). ^13^C NMR (100 MHz, CDCl_3_) δ (ppm): 185.57 (C9), 165.85 (C11), 137.86 (C7), 136.79 (C8), 136.17–136.79, 132.47–132.74 (C6), 131.82, 131.33, 131.14, 129.21–129.40, 50.02 (C12), 43.69–45.69 (C10). IR (KBr, ν/cm^−1^): 2101 (N_3_), 1672, 1656, 1611 and 1582 (C=O), 1488, 1466, 1402, 1280, 1258, 1227, 1167, 1073, 1009, 991, 836, 826, 518. HRMS (ESI+) of C_21_H_16_Br_2_N_4_O_2_, *m*/*z*: calcd for [M+H]^+^516.9693, found 516.9695.

*(3E,5E)-1-(2-azidoacetyl)-3,5-bis(4-methoxybenzylidene)-piperidin-4-one (* **20** *)*


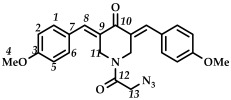


M.p. ˃ 147. °C (decomposition with release of N_2_ gas). ^1^H NMR (300 MHz, CDCl_3_) δ (ppm): 7.71 (2H, s, H-8), 6.88–7.32 (8H, m, H-1, H-2, H-5, H-6), 4.53, 4.82 (4H, s, H-11), 3.77 (2H, s, H-13). ^13^C NMR (100 MHz, CDCl_3_) δ (ppm): 185.30 (C10), 166.05 (C12), 160.82, 137.26–138.23 (C8), 132.24–132.77 (C9), 128.94, 126.70–127.15, 114.33–114.48, 55.57 (C4), 49.74 (C13), 44.19–46.18 (C11). IR (KBr, ν/cm^−1^): 2108 (N_3_), 1678, 1655, 1595 and 1567 (C=O), 1511, 1456, 1423, 1263, 1169, 1031, 993, 823, 529. HRMS (ESI+) of C_23_H_22_N_4_O_4_, *m*/*z*: calcd for [M+H]^+^419.1714, found 419.1720.

*(3E,5E)-1-(2-azidoacetyl)-3,5-bis(4-isopropylbenzylidene)-piperidin-4-one (* **21** *)*


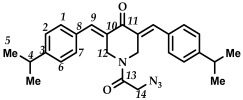


M.p. ˃ 110 °C (decomposition with release of N_2_ gas). ^1^H NMR (300 MHz, CDCl_3_) δ (ppm): 7.83, 7.85 (2H, s, H-9), 7.26–7.38 (8H, m, H-1, H-2, H-6, H-7), 4.63, 4.93 (4H, s, H-12), 3.77 (2H, s, H-14), 2.92–2.97 (2H, m, H-4), 1.27, 1.28 (14H, s, H-5, H-14). ^13^C NMR (100 MHz, CDCl_3_) δ (ppm): 186.07 (C11), 166.36 (C13), 151.04–151.16, 138.88–139.03, 137.80–137.96, 131.66–132.04, 131.00, 130.49, 130.13–130.19, 126.99–127.18, 50.25 (C14), 44.00–45.73 (C12), 34.05–34.12 (C5), 23.65–23.93 (C4). IR (KBr, ν/cm^−1^): 2961 (CH), 2871, 2105 (N_3_), 1660, 1606, 1575 and 1509 (C=O), 1459, 1419, 1276, 1232, 1175, 1056, 1017, 992, 834, 559. HRMS (ESI+) of C_27_H_30_N_4_O_2_, *m*/*z*: calcd for [M+H]^+^ 443.2442, found 443.2447.

*(3E,5E)-1-(2-azidoacetyl)-3,5-bis(3,4,5-trimethoxybenzylidene)-piperidin-4-one (* **22** *)*


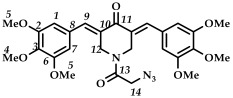


M.p. ˃ 180 °C (decomposition with release of N_2_ gas). ^1^H NMR (300 MHz, CDCl_3_) δ (ppm): 7.71 (2H, s, H-9), 6.54–6.66 (4H, s, H-1, H-7), 4.66–4.90 (4H, s, H-12), 3.86 (18H, s, H-4, H-5), 3.82 (2H, s, H-14). ^13^C NMR (100 MHz, CDCl_3_) δ (ppm): 185.80 (C11), 166.07 (C13), 153.23–153.35, 138.12–139.72, 129.85–130.16, 107.68–107.96, 60.95 (C5), 56.25 (C4), 50.50 (C14), 40.91–45.96 (C12). IR (KBr, ν/cm^−1^): 2110 (N_3_), 1648, 1602, 1580 and 1507 (C=O), 1449, 1435, 1419, 1274, 1254, 1161, 1132, 1034, 999, 830, 622, 594. HRMS (ESI+) of C_27_H_30_N_4_O_8_, *m*/*z*: calcd for [M+H]^+^ 539.2137, found 539.2144; for [M+Na]^+^ 561.1956, found 561.1962.

### 3.6. General Procedure of (3E,5E)-1-(2-(4-((10H-Phenothiazine-10-yl)methyl)-1H-1,2,3-triazol-1-yl)acetyl)-3,5-bis(benzylidene)-piperidin-4-ones (***23***–***29***) Synthesis

To a stirred mixture of 10-(prop-2-yn-1-yl)-10H-phenothiazine **1** (0.2 mmoL, 1.0 eq.) and the corresponding azides **16**–**22** (0.2 mmoL, 1.0 eq.) in methylene chloride CH_2_Cl_2_ (5 mL), copper(I) bromide (0.01 mmoL, 5 mol.%) and DIPEA (0.02 mmol, 10 mol.%) were added. The solution obtained was stirred at room temperature (TLC monitoring). The solvent was removed in vacuo, and the remaining crude product was purified via column chromatography (chloroform:methanol 100:1.5 (*v*/*v*) for compounds **23**–**28** and 100:2.5 (*v*/*v*) for compound **29**).

*(3E,5E)-1-(2-(4-((10H-phenothiazine-10-yl)methyl)-1H-1,2,3-triazol-1-yl)acetyl)-3,5-bis(benzylidene-piperidin-4-one (* **23** *)*


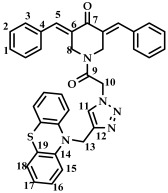


M.p. = 170–173 °C. ^1^H NMR (300 MHz, CDCl_3_) δ (ppm): 7.87, 7.94 (2H, s, H-5), 7.40–7.50 (11H, m, H-1, H-2, H-3, H-11), 6.75–7.11 (8H, m, H-15, H-16, H-17, H-18), 5.19 (2H, s, H-10), 4.98 (2H, s, H-13), 4.90, 4.74 (4H, s, H-8). ^13^C NMR (100 MHz, CDCl_3_) δ (ppm): 185.52 (C7), 163.51 (C9), 144.27–145.09 (C5), 138.45–139.36 (C6), 129.86–130.65, 128.87–129.22, 127.14–127.43, 123.82–124.12, 122.84, 115.22, 50.77 (C13), 45.87 (C10), 44.88, 44.08 (C8). HRMS (ESI+) of C_36_H_30_N_5_O_2_S, *m*/*z*: calc for [M+H]^+^ 596.2115, found 596.2097; for [M+Na]^+^ 618.1934, found 618.1928.

*(3E,5E)-1-(2-(4-((10H-phenothiazine-10-yl)methyl)-1H-1,2,3-triazol-1-yl)acetyl)-3,5-bis(4-fluorobenzylidene)-piperidin-4-one (* **24** *)*


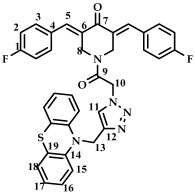


M.p. = 177–180 °C. ^1^H NMR (300 MHz, CDCl_3_) δ (ppm): 7.77, 7.83 (2H, s, H-5), 7.41–7.62 (8H, m, H-2, H-3), 6.75–7.11 (9H, m, H-15, H-16, H-17, H-18, H-11), 5.20 (2H, s, H-10), 5.01 (2H, s, H-13), 4.83, 4.70 (4H, s, H-8). ^13^C NMR (100 MHz, CDCl_3_) δ (ppm): 185.29 (C7), 163.56 (C9), 144.20 (C5), 137.27–138.02 (C6), 132.65, 132.56, 130.07, 127.38, 127.16, 123.94, 122.81, 116.58, 116.21, 115.99, 115.27, 50.97 (C13), 45.97 (C10), 44.78, 43.82 (C8). ^19^F NMR, δ (ppm): −108.51, −109.17. HRMS (ESI+) of C_36_H_27_F_2_N_5_O_2_S, *m*/*z*: calc for [M+H]^+^ 632.1926, found 632.1913; for [M+Na]^+^ 654.1746, found 654.1739.

*(3E,5E)-1-(2-(4-((10H-phenothiazine-10-yl)methyl)-1H-1,2,3-triazol-1-yl)acetyl)-3,5-bis(4-chlorobenzylidene)-piperidin-4-one (* **25** *)*


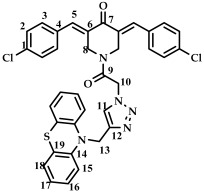


M.p. = 190–191 °C. ^1^H NMR (300 MHz, CDCl_3_) δ (ppm): 7.77, 7.85 (2H, s, H-5), 7.33–7.49 (9H, m, H-2, H-3, H-11), 6.74–7.11 (8H, m, H-15, H-16, H-17, H-18), 5.19 (2H, s, H-10), 5.02 (2H, s, H-13), 4.83, 4.71 (4H, s, H-8). ^13^C NMR (100 MHz, CDCl_3_) δ (ppm): 185.27 (C7), 163.55 (C9), 144.29–144.35 (C5), 137.50–137.74 (C6), 129.52, 129.21, 127.38, 127.17, 124.05, 123.89, 122.82, 115.16, 50.89 (C13), 45.97 (C10), 44.65, 43.87 (C8). HRMS (ESI+) of C_36_H_27_Cl_2_N_5_O_2_S, *m*/*z*: calcd for [M+H]^+^ 664.1319, found 664.1335; for [M+Na]^+^ 686.1146, found 686.1155.

*(3E,5E)-1-(2-(4-((10H-phenothiazin-10-yl)methyl)-1H-1,2,3-triazol-1-yl)acetyl)-3,5-bis(4-bromobenzylidene)-piperidin-4-one (* **26** *)*


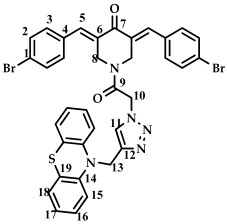


M.p. = 225–228 °C. ^1^H NMR (300 MHz, CDCl_3_) δ (ppm): 7.78, 7.85 (2H, s, H-5), 7.51–7.64 (8H, m, H-2, H-3), 7.12 (1H, s, H-11), 6.75–7.10 (8H, m, H-15, H-16, H-17, H-18), 5.21 (2H, s, H-10), 5.01 (2H, s, H-13), 4.84, 4.71 (4H, s, H-8). HRMS (ESI+) of C_36_H_27_Br_2_N_5_O_2_S, *m*/*z*: calcd for [M+H]^+^ 754.0305, found 754.0291; for [M+Na]^+^ 776.0124, found 776.0126.

*(3E,5E)-1-(2-(4-((10H-phenothiazine-10-yl)methyl)-1H-1,2,3-triazol-1-yl)acetyl)-3,5-bis(4-methoxybenzylidene)-piperidin-4-one (* **27** *)*


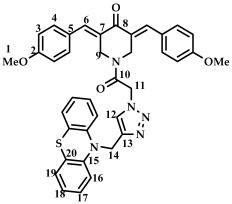


M.p. = 209–212 °C. ^1^H NMR (300 MHz, CDCl_3_) δ (ppm): 7.76, 7.82 (2H, s, H-6), 6.73–7.69 (17H, m, H-3, H-4, H-12, H-16, H-17, H-18, H-19), 5.16 (2H, s, H-11), 5.03 (2H, s, H-14), 4.84, 4.70 (4H, s, H-9), 3.86 (6H, s, H-1). ^13^C NMR (100 MHz, CDCl_3_) δ (ppm): 185.55 (C8), 161.02 (C10), 144.18–145.00 (C6), 137.82–138.69 (C7), 128.49, 127.10–127.40, 127.02, 124.24, 123.70, 122.76, 55.43 (C1), 50.78 (C14), 45.98 (C11), 44.89, 44.08 (C9). HRMS (ESI+) of C_38_H_33_N_5_O_4_S, *m*/*z*: calcd for [M+H]^+^ 656.2326, found 656.2315; for [M+Na]^+^ 678.2145, found 678.2139.

*(3E,5E)-1-(2-(4-((10H-phenothiazine-10-yl)methyl)-1H-1,2,3-triazol-1-yl)acetyl)-3,5-bis(4-isopropylbenzylidene)-piperidin-4-one (* **28** *)*


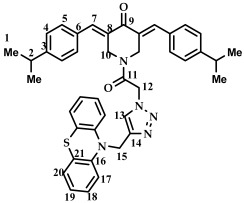


M.p. = 165–170 °C. ^1^H NMR (300 MHz, CDCl_3_) δ (ppm): 7.93, 7.99 (2H, s, H-7), 6.84–7.51 (17H, m, H-4, H-5, H-13, H-17, H-18, H-19, H-20), 5.29 (2H, s, H-12), 5.15 (2H, s, H-15), 4.98, 4.85 (4H, s, H-10), 2.99–3.10 (2H, m, H-2), 1.37 (6H, d, H-1). ^13^C NMR (100 MHz, CDCl_3_) δ (ppm): 185.58 (C9), 163.45 (C11), 151.10–151.39 (C7), 144.15 (C8), 138.36–139.19, 130.60–130.89, 126.99–127.39, 122.78–123.80, 115.25, 50.86 (C15), 46.02 (C12), 44.11, 44.87 (C10), 34.10 (C2), 23.77 (C1). HRMS (ESI+) of C_42_H_41_N_5_O_2_S, *m*/*z*: calcd for [M+H]^+^ 680.3054, found 680.3044; for [M+Na]^+^ 702.2873, found 702.2866.

*(3E,5E)-1-(2-(4-((10H-phenothiazine-10-yl)methyl)-1H-1,2,3-triazol-1-yl)acetyl)-3,5-bis(3,4,5-trimethoxybenzylidene)-piperidin-4-one (* **29** *)*


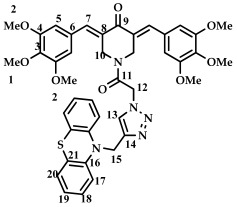


M.p. = 165–167 °C. ^1^H NMR (300 MHz, CDCl_3_) δ (ppm): 7.76, 7.84 (2H, s, H-7), 7.45 (1H, s, H-13), 6.61–7.10 (12H, m, H-5, H-17, H-18, H-19, H-20), 5.19 (2H, s, H-12), 5.06 (2H, s, H-15), 4.90, 4.83 (4H, s, H-10), 3.87, 3.90, 3.93 (18H, s, H-1, H-2). ^13^C NMR (100 MHz, CDCl_3_) δ (ppm): 185.06 (C9), 163.88 (C11), 153.06 (C7), 144.08–145.46 (C8), 138.46–139.51, 129.75, 127.32, 124.07, 123.52, 122.72, 115.12, 107.77, 61.21 (C1), 56.54 (C2), 50.86 (C15), 46.52 (C12), 44.13, 44.93 (C10). HRMS (ESI+) of C_42_H_41_N_5_O_8_S, *m*/*z*: calcd for [M+H]^+^ 776.2749, found 776.2741; for [M+Na]^+^ 798.2568, found 798.2562.

### 3.7. General Procedure of (3E,5E)-1-(2-(4-((10H-Phenothiazine-10-yl)methyl)-1H-1,2,3-triazol-1-yl)acetyl)-3,5-bis(benzylidene)-piperidin-4-one Hydrochlorides (***30***–***36***) Synthesis

To obtain the hydrochloride salts, piperidones **23**–**29** were dissolved in methylene chloride and hydrogen chloride, obtained by heating hydrochloric acid, which was passed into the solution until the pH of the solution reached 1. After the desired pH was reached, the methylene chloride was removed under vacuum until constant weight. The product was used for further studies without further purification.

*(3E,5E)-1-(2-(4-((10H-phenothiazine-10-yl)methyl)-1H-1,2,3-triazol-1-yl)acetyl)-3,5-bis(benzylidene)-piperidin-4-one hydrochloride (* **30** *)*


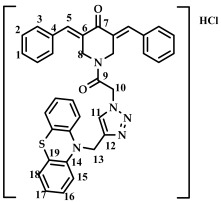


M.p. = 170–173 °C. ^1^H NMR (300 MHz, CDCl_3_) δ (ppm): 7.87, 7.94 (2H, br. s, H-5), 7.43–7.47 (11H, br. m, H-1, H-2, H-3, H-11), 6.74–7.11 (8H, br. m, H-15, H-16, H-17, H-18), 5.19 (2H, br. s, H-10), 4.98 (2H, br. s, H-13), 4.90, 4.74 (4H, br. s, H-8).

*(3E,5E)-1-(2-(4-((10H-phenothiazine-10-yl)methyl)-1H-1,2,3-triazol-1-yl)acetyl)-3,5-bis(4-fluorobenzylidene)-piperidin-4-one hydrochloride (* **31** *)*


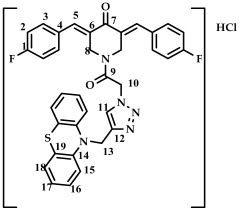


M.p. = 148–152 °C. ^1^H NMR (300 MHz, CDCl_3_) δ (ppm): 7.89, 7.82 (2H, br. s, H-5), 7.22–7.41 (9H, br. m, H-2, H-3, H-11), 6.76–7.12 (8H, br. m, H-15, H-16, H-17, H-18), 5.23 (2H, br. s, H-10), 5.08 (2H, br. s, H-13), 4.86, 4.74 (4H, br. s, H-8).

*(3E,5E)-1-(2-(4-((10H-phenothiazine-10-yl)methyl)-1H-1,2,3-triazol-1-yl)acetyl)-3,5-bis(4-chlorobenzylidene)-piperidin-4-one hydrochloride (* **32** *)*


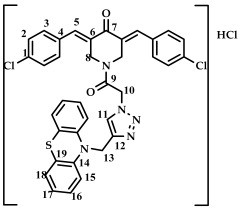


M.p. = 121–122 °C. ^1^H NMR (300 MHz, CDCl_3_) δ (ppm): ^1^H NMR (300 MHz, CDCl_3_) δ (ppm): 789, 7.82 (2H, br. s, H-5), 7.41 (4H, br. m, H-2, H-3), 6.75–7.11 (9H, br. m, H-15, H-16, H-17, H-18, H-11), 5.23 (2H, br. s, H-10), 5.08 (2H, br. s, H-13), 4.86, 4.74 (4H, br. s, H-8). ^13^C NMR (100 MHz, CDCl_3_) δ (ppm): 185.31 (C7), 163.44 (C9), 144.19–145.16 (C5), 137.29–138.04 (C6), 131.74, 131.50, 129.21, 127.38, 127.17, 124.05, 123.89, 122.82, 115.23, 51.04 (C13), 45.75 (C10), 44.73, 43.87 (C8).

*(3E,5E)-1-(2-(4-((10H-phenothiazine-10-yl)methyl)-1H-1,2,3-triazol-1-yl)acetyl)-3,5-bis(4-methoxybenzylidene)-piperidin-4-one hydrochloride (* **34** *)*


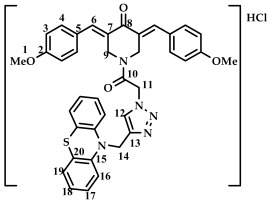


M.p. = 209–210 °C. ^1^H NMR (300 MHz, CDCl_3_) δ (ppm): 7.81, 7.87 (2H, br. s, H-6), 6.75–7.43 (17H, br. m, H-3, H-4, H-12, H-16, H-17, H-18, H-19), 5.19 (2H, br. s, H-11), 5.03 (2H, br. s, H-14), 4.88, 4.73 (4H, br. s, H-9), 3.86, 3.88 (6H, br. s, H-1). ^13^C NMR (100 MHz, CDCl_3_) δ (ppm): 185.49 (C8), 163.67 (C10), 144.31–145.10 (C6), 137.87–138.81 (C7), 128.73, 127.40, 127.10, 126.48, 124.24, 123.70, 122.72, 55.79 (C1), 50.75 (C14), 46.03 (C11), 43.78 (C9).

*(3E,5E)-1-(2-(4-((10H-phenothiazine-10-yl)methyl)-1H-1,2,3-triazol-1-yl)acetyl)-3,5-bis(4-isopropylbenzylidene)-piperidin-4-one hydrochloride (* **35** *)*


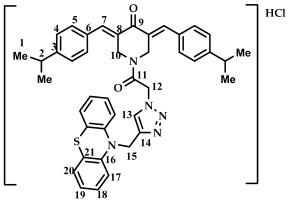


M.p. = 192–193 °C. ^1^H NMR (300 MHz, CDCl_3_) δ (ppm): 7.93, 7.99 (2H, br. s, H-7), 6.85–7.55 (17H, br. m, H-4, H-5, H-13, H-17, H-18, H-19, H-20), 5.30 (2H, br. s, H-12), 5.16 (2H, br. s, H-15), 4.99, 4.85 (4H, br. s, H-10), 3.01–3.06 (2H, br. m, H-2), 1.36–1.38 (6H, br. d, H-1). ^13^C NMR (100 MHz, CDCl_3_) δ (ppm): 185.71 (C9), 163.52 (C11), 151.12–151.41 (C7), 144.30 (C8), 139.22–138.27, 130.88–130.60, 127.40–127.00, 122.78, 115.12, 51.27 (C15), 46.28 (C12), 44.15, 44.96 (C10), 34.10 (C2), 23.48 (C1).

*(3E,5E)-1-(2-(4-((10H-phenothiazine-10-yl)methyl)-1H-1,2,3-triazol-1-yl)acetyl)-3,5-bis(3,4,5-trimethoxybenzylidene)-piperidin-4-one hydrochloride (* **36** *)*


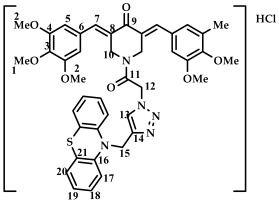


M.p. = 104–106 °C. ^1^H NMR (300 MHz, CDCl_3_) δ (ppm): 7.78, 7.86 (2H, br. s, H-7), 7.45 (1H, br. s, H-13), 6.62–7.11 (12H, br. m, H-5, H-17, H-18, H-19, H-20), 5.20 (2H, br. s, H-12), 5.05 (2H, br. s, H-15), 4.91, 4.84 (4H, br. s, H-10), 3.88, 3.91, 3.94 (18H, br. s, H-1, H-2). ^13^C NMR (100 MHz, CDCl_3_) δ (ppm): 185.93 (C9), 164.22 (C11), 153.07 (C7), 138.70, 137.07, 136.48, 132.36, 130.57, 128.36, 127.80, 127.52, 126.78, 124.90, 115.12, 107.78, 61.20 (C1), 56.23 (C2), 54.18 (C15), 52.23 (C12), 51.73 (C10).

### 3.8. Methods

^1^H NMR were recorded on a Bruker Avance 300 spectrometer (Bruker, Rheinstetten, Germany) operating at 300 MHz. ^13^C NMR and ^19^F NMR were recorded on a Bruker Avance 400 spectrometer (Bruker, Rheinstetten, Germany) operating at 100.6 MHz and 376.5 MHz, respectively. Deuterochloroform (≥99.8 atom % D, Sigma Aldrich, Saint Louis, MO, USA) was used as a solvent for recording NMR spectra. Chloroform solvent signals (δ_H_ 7.24 ppm, δ_C_ 76.90 ppm) were used as an internal standard.

High-resolution mass spectra were recorded on a LCMS-9030 device (Shimadzu, Kyoto, Japan) by electrospray ionization mass spectrometry (ESI-MS). Measurements were carried out in positive ion mode; samples were dissolved in acetonitrile (≥99.7%, Component-Reactive, Moscow, Russia) and injected into the mass-spectrometer chamber from an HPLC system LC-40 Nexera (Shimadzu, Japan). The following parameters were used: capillary voltage: 4.0 kV; mass scanning range: *m*/*z* 100–1000; external calibration with solution NaI (99.9%, Merck, Darmstadt, Germany) in MeOH (≥99.5%, JSC Vekton, Saint Petersburg, Russia)/H_2_O; drying and heating gases (nitrogen) (each 10 L/min); nebulizing gas (nitrogen) (3 L/min); interface temperature: 250 C; flow rate 100% methanol 0.4 mL/min. Molecular ions in the spectra were analyzed and matched with the appropriately calculated *m*/*z* and isotopic profiles in the LabSolutions v.5.114 program (Shimadzu, Kyoto, Japan).

IR spectra were recorded in film or KBr pellets on a Fourier-transform spectrometer “Magna-IR750” (Nicolet, Glendale, WI, USA), with a resolution of 2 cm^−1^ and 128 scans.

Panc-1 (pancreatic adenocarcinoma), T-24 (bladder cancer), T98G (glioblastoma), BT-20 (breast adenocarcinoma), and DF-1 (dermal fibroblasts) cell lines were cultured under standard conditions at 37 °C in an atmosphere with 5% CO_2_ and high humidity. Cells were obtained from the collection of the Institute of Cytology of the Russian Academy of Sciences (St. Petersburg, Russia). DMEM (Dulbecco’s Modified Eagle Medium, Biolot, St. Petersburg, Russia) was used for Panc-1, and EMEM (Eagle’s Minimum Essential Medium, Biolot, Russia) for T-24, T98G, and BT-20. DF-1 cells were cultured in DMEM/F12 (Biolot). All types of culture media were supplemented with fetal bovine serum (10%, Biolot, Russia), 2 mM L-glutamine, and penicillin-streptomycin. Cells were maintained in logarithmic growth phase by passage every 2–4 days and dissociated using a trypsin-versene solution (at a 1:3 ratio) after reaching 80–90% confluency. Before experiments, cells were adapted in 96-well plates at a concentration of 10^4^ cells/well for 24 h to allow for adhesion and stabilization of their physiological state.

The test compounds were dissolved in DMSO to prepare stock solutions (10 mM). Working solutions were prepared immediately before experiments by serial dilution.

The cytotoxic effect of the compounds was determined using the MTT assay (3-(4,5-dimethylthiazol-2-yl)-2,5-diphenyltetrazolium bromide), which is based on the reduction in MTT by active mitochondrial dehydrogenases to insoluble purple formazan. After 72 h incubation of cells with the compounds (in the concentration range from 0.01 to 30 μM, the DMSO content did not exceed 1%), MTT solution (0.5 mg/mL, Sigma-Aldrich, Saint Louis, MO, USA) was added to the cells and they were incubated for 2 h at 37 °C. The formazan crystals that formed were dissolved by adding DMSO with subsequent gentle mixing. Optical density was measured using a plate spectrophotometer (at λ = 530 nm). The IC_50_ values were calculated as parameters of the effectiveness of the cytotoxic effect of the compounds. All experiments were conducted in triplicate.

The docking analysis of the molecules was carried out using AutoDock 4.2.6 (Scripps Research, La Jolla, CA, USA, 2014). Ligand molecules were sketched in 3D format using the ChemBio3D v. 14 (PerkinElmer, Shelton, Connecticut, USA) program. The Universal Force Field minimization algorithm was used to produce low-energy conformers. The structural coordinates of the ATP-binding cleft of EGFR tyrosine kinase (PDB ID: 1M14) were obtained from the protein databank (PDB). Re-docking of minimized molecules was performed to validate the docking algorithms of AutoDock 4.2. The lowest energy conformations in the binding site of the protein were determined. The Python Molecular Viewer 1.5.7 was utilized for docking visualization.

## 4. Conclusions

We developed a method for synthesizing 3,5-bis(benzylidene)-4-piperidones with various substituents on the aromatic ring (halogens, single or three methoxy groups, and an isopropyl group) and phenothiazine. Seven compounds were synthesized as free bases (**23**–**29**) and seven of their hydrochloride salts (**30**–**36**). The structures of compounds **23**–**29** were confirmed by ^1^H and ^13^C NMR spectroscopy and mass spectrometry.

In vitro cytotoxicity assays showed that hydrochloride salts **30**–**36** exhibited more potent antitumor activity against Panc-1 pancreatic adenocarcinoma cells compared to the parent piperidones **2**–**8**, which lack the phenothiazine moiety, and free bases **23**–**29**. Among the tested compounds, compounds **29** and **36**, which contain three methoxy groups at the aromatic ring, were found to be the most effective against pancreatic cancer cells Panc-1, bladder cancer T-24, glioblastoma T98G, and breast cancer BT-20. All tested compounds exhibited selectivity for tumor cells over normal human dermal fibroblast cells DF-1. Moreover, the docking results indicate that compound **29** exhibits a strong binding affinity to EGFR in the binding pocket of the clinically used inhibitor erlotinib.

Overall, based on the results obtained during in vitro cytotoxicity studies and docking, it can be concluded that compound **29** and its hydrochloride salt **36** can be considered potential candidates for the further study of antitumor mechanisms of cancer cell death.

## Figures and Tables

**Table 1:** Cytotoxic effect of the parent piperidones and phenothiazine conjugates against the pancreatic adenocarcinoma cell line (Panc-1).

Compound	Panc-1	Compound	Panc-1	Compound	Panc-1
	IC_50_, μM		IC_50_, μM		IC_50_, μM
**2**	16.11 ± 0.23	**23**	8.57 ± 0.04	**30**	7.08 ± 0.46
**3**	14.92 ± 0.90	**24**	8.10 ± 0.38	**31**	5.36 ± 0.11
**4**	11.21 ± 0.04	**25**	7.71 ± 0.16	**32**	5.47 ± 0.17
**5**	>30	**26**	>30	**33**	>30
**6**	>30	**27**	>30	**34**	>30
**7**	>30	**28**	>30	**35**	>30
**8**	9.01 ± 0.02	**29**	3.37 ± 0.05	**36**	0.73 ± 0.02
Phenothiazine			>30		
Curcumin			>30		

**Table 2:** Cytotoxic profile of 3,5-bis(benzylidene)-4-piperidones and phenothiazine conjugates.

Compound	T-24	T98G	BT-20	DF-1
**23**	8.60 ± 0.03	8.33 ± 0.27	29.11 ± 1.53	>30
**24**	8.12 ± 0.14	8.21 ± 0.09	28.76 ± 2.30	>30
**25**	6.36 ± 0.01	7.53 ± 0.21	28.45 ± 0.96	>30
**26**	>30	>30	>30	>30
**27**	>30	>30	>30	>30
**28**	>30	>30	>30	>30
**29**	2.39 ± 0.02	4.01 ± 0.16	26.05 ± 1.39	>30
**30**	5.73 ± 0.01	4.91 ± 0.08	27.94 ± 1.30	>30
**31**	6.11 ± 0.20	4.76 ± 0.24	24.41 ± 2.05	>30
**32**	4.95 ± 0.00	4.10 ± 0.05	27.00 ± 0.94	>30
**33**	>30	>30	>30	>30
**34**	>30	>30	>30	>30
**35**	>30	>30	>30	>30
**36**	0.59 ± 0.00	1.25 ± 0.10	19.69 ± 0.78	>30
Phenothiazine	>30	>30	>30	>30
Curcumin	8.49 ± 0.37	9.23 ± 0.14	>30	>30

## Data Availability

The original contributions presented in this study are included in the article/Supplementary Materials. Further inquiries can be directed to the corresponding authors.

## Supplementary Materials

The following supporting information can be downloaded at: https://www.mdpi.com/article/10.3390/ijms27094104/s1.

## Abbreviations

The following abbreviations and symbols are used in this manuscript:

AMPK	AMP-activated protein kinase
ATP	Adenosine triphosphate
DIPEA	Diisopropylethylamine
DMEM	Dulbecco’s Modified Eagle Medium
DMSO	Dimethylsulfoxide
DNA	Deoxyribonucleic acid
EGFR	Epidermal growth factor receptor
ESI-MS	Electrospray ionization mass spectrometry
GSH	Reduced glutathione
GST	Glutathione-S-transferase
MTT	3-(4,5-Dimethylthiazol-2-yl)-2,5-diphenyltetrazolium bromide
NMR	Nuclear magnetic resonance
ppm	Past per million
