# Supplementary material for: Synthesis and Cytotoxic Activity Study of Conjugates of N-Acyl Derivatives of 3,5-Bis(benzylidene)-4-piperidones and Phenothiazine
Source: Int J Mol Sci. 2026 May 4;27(9):4104. doi: 10.3390/ijms27094104 (PMC13164472; doi:10.3390/ijms27094104)
Supplement: Supplementary file 1 [file ijms-27-04104-s001.zip › ijms-4234662-supplementary.pdf]

Synthesis and Cytotoxic Activity Study of Conjugates of N-acyl Derivatives of 3,5-bis(benzylidene)-4-piperidones and Phenothiazine

Pavel Yudaev [1,†], Yulia Aleksandrova [1,†], Inna Shagina [1], Oleg Artyushin [1], Elena Sharova [1], Aleksei Rodionov [1], Margarita Neganova [1,*] and Valery Brel [1,*]

[1] Nesmeyanov Institute of Organoelement Compounds, Russian Academy of Sciences, Vavilova St., 28, Bld. 1, Moscow 119991, Russia
[†] Contribution equally

* Correspondence: v_brel@mail.ru (V.B.); neganovam@ineos.ac.ru (M.N.)

1. *Methods* 2

2. *Figure S1-S22. NMR and HRMS (ESI+) spectra of (3E,5E)-1-(2-azidoacetyl)-3,5-bis(benzylidene)-piperidin-4-ones (**16-22**)* 3

2.1 *Figure S1-S3. Spectra of (3E,5E)-1-(2-azidoacetyl)-3,5-bis(benzylidene)-piperidin-4-one (**16**)* 3

2.2 *Figure S4-S7. Spectra of (3E,5E)-1-(2-azidoacetyl)-3,5-bis(4-fluorobenzylidene)-piperidin-4-one (**17**)* 4

2.3 *Figure S8-S10. Spectra of (3E,5E)-1-(2-azidoacetyl)-3,5-bis(4-chlorobenzylidene)-piperidin-4-one (**18**)* 6

2.4 *Figure S11-S13. Spectra of (3E,5E)-1-(2-azidoacetyl)-3,5-bis(4-bromobenzylidene)-piperidin-4-one (**19**)* 8

2.5 *Figure S14-S16. Spectra of (3E,5E)-1-(2-azidoacetyl)-3,5-bis(4-methoxybenzylidene)-piperidin-4-one (**20**)* 9

2.6 *Figure S17-S19. Spectra of (3E,5E)-1-(2-azidoacetyl)-3,5-bis(4-isopropylbenzylidene)-piperidin-4-one (**21**)* 11

2.7 *Figure S20-S22. Spectra of (3E,5E)-1-(2-azidoacetyl)-3,5-bis(3,4,5-trimethoxybenzylidene)-piperidin-4-one (**22**)* 13

3. *Figure S23-S43. NMR and HRMS (ESI+) spectra of (3E,5E)-1-(2-(4-((10H-phenothiazine-10-yl)methyl)-1H-1,2,3-triazol-1-yl)acetyl)-3,5-bis(benzylidene)-piperidin-4-ones (**23-29**)* 15

3.1 *Figure S23-S25. Spectra of (3E,5E)-1-(2-(4-((10H-phenothiazine-10-yl)methyl)-1H-1,2,3-triazol-1-yl)acetyl)-3,5-bis(benzylidene)-piperidin-4-one (**23**)* 15

3.2 *Figure S26-S29. Spectra of (3E,5E)-1-(2-(4-((10H-phenothiazine-10-yl)methyl)-1H-1,2,3-triazol-1-yl)acetyl)-3,5-bis(4-fluorobenzylidene)-piperidin-4-one (**24**)* 17

3.3 *Figure S30-S32. Spectra of (3E,5E)-1-(2-(4-((10H-phenothiazine-10-yl)methyl)-1H-1,2,3-triazol-1-yl)acetyl)-3,5-bis(4-chlorobenzylidene)-piperidin-4-one (**25**)* 19

3.4 *Figure S33-S34. Spectra of (3E,5E)-1-(2-(4-((10H-phenothiazine-10-yl)methyl)-1H-1,2,3-triazol-1-yl)acetyl)-3,5-bis(4-bromobenzylidene)-piperidin-4-one (**26**)* 21

3.5 *Figure S35-S37. Spectra of (3E,5E)-1-(2-(4-((10H-phenothiazine-10-yl)methyl)-1H-1,2,3-triazol-1-yl)acetyl)-3,5-bis(4-methoxybenzylidene)-piperidin-4-one (**27**)* 22

3.6 *Figure S38-S40. Spectra of (3E,5E)-1-(2-(4-((10H-phenothiazine-10-yl)methyl)-1H-1,2,3-triazol-1-yl)acetyl)-3,5-bis(4-isopropylbenzylidene)-piperidin-4-one (**28**)* 24

3.7 *Figure S41-S43. Spectra of (3E,5E)-1-(2-(4-((10H-phenothiazine-10-yl)methyl)-1H-1,2,3-triazol-1-yl)acetyl)-3,5-bis(3,4,5-trimethoxybenzylidene)-piperidin-4-one (**29**)* 26

4. *Figure S44-S53. NMR spectra of (3E,5E)-1-(2-(4-((10H-phenothiazine-10-yl)methyl)-1H-1,2,3-triazol-1-yl)acetyl)-3,5-bis(benzylidene)-piperidin-4-ones hydrochloride (**30-36**)* 27

1. *Methods*

^{1}H NMR were recorded on a Bruker Avance 300 spectrometer (Bruker, Rheinstetten, Germany) operating at 300 MHz. ^{13}C NMR and ^{19}F NMR were recorded on a Bruker Avance 400 spectrometer (Bruker, Rheinstetten, Germany) operating at 100.6 MHz and 376.5 MHz respectively. Deuterochloroform (≥99.8 atom % D, Sigma Aldrich, Saint Louis, MO, USA) was used as a solvent for recording NMR spectra. Chloroform solvent signals (δ_H 7.24 ppm, δ_C 76.90 ppm) were used as an internal standard.

High-resolution mass spectra were recorded on a LCMS-9030 device (Shimadzu, Japan) by electrospray ionization mass spectrometry (ESI-MS). Measurements were carried out in positive ion mode; samples were dissolved in acetonitrile and injected into the mass-spectrometer chamber from an HPLC system LC-40 Nexera (Shimadzu, Japan). The following parameters were used: capillary voltage 4.0 kV; mass scanning range: m/z 100–1000; external calibration with solution NaI in MeOH/H_2O; drying and heating gases (nitrogen) (each 10 L/min); nebulizing gas (nitrogen) (3 L/min); interface temperature: 25^{0}C; flow rate 100% methanol 0.4 mL/min. Molecular ions in the spectra were analyzed and matched with the appropriately calculated m/z and isotopic profiles in the LabSolutions v.5.114 program (Shimadzu, Kyoto, Japan).

2. *Figure S1-S22. NMR and HRMS (ESI+) spectra of (3E,5E)-1-(2-azidoacetyl)-3,5-bis(benzylidene)-piperidin-4-ones (**16-22**)*

2.1 *Figure S1-S3. Spectra of (3E,5E)-1-(2-azidoacetyl)-3,5-bis(benzylidene)-piperidin-4-one (**16**)*

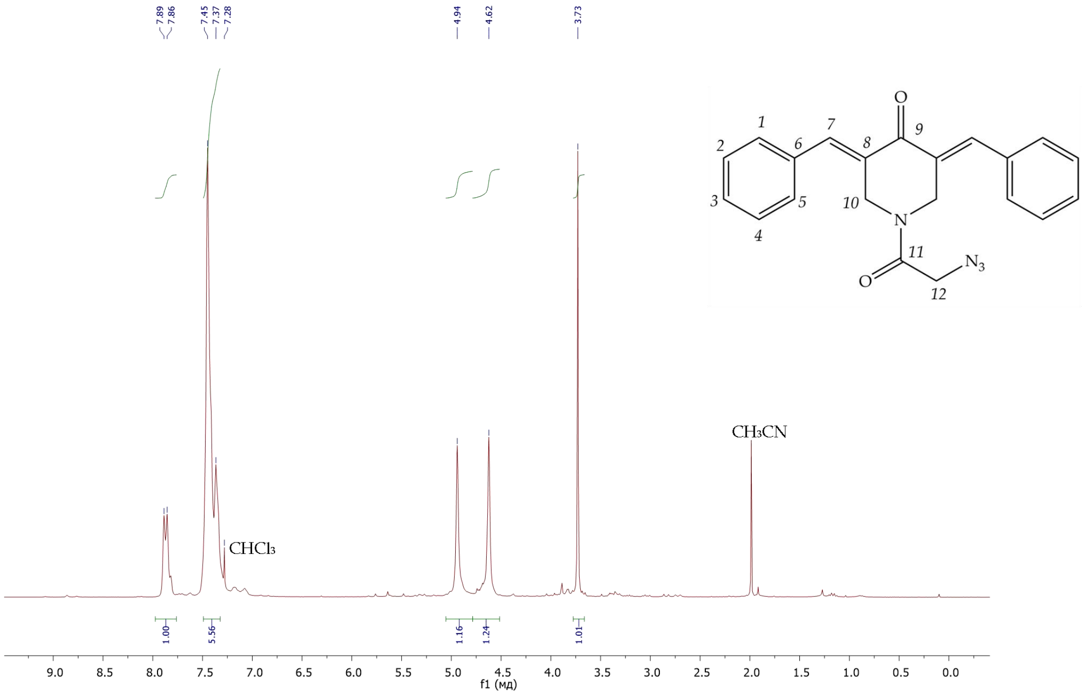

Figure S1. ^{1}H NMR spectrum of (*3E,5E*)-1-(2-azidoacetyl)-3,5-bis(benzylidene)-piperidin-4-one (**16**).

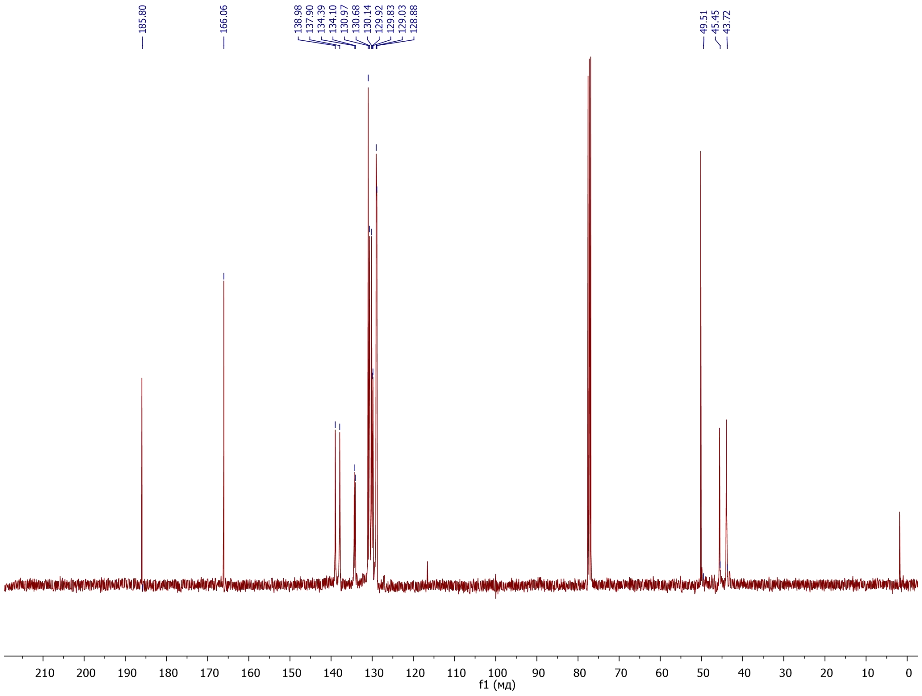

Figure S2. ^{13}C NMR spectrum of (*3E,5E*)-1-(2-azidoacetyl)-3,5-bis(benzylidene)-piperidin-4-one (**16**).

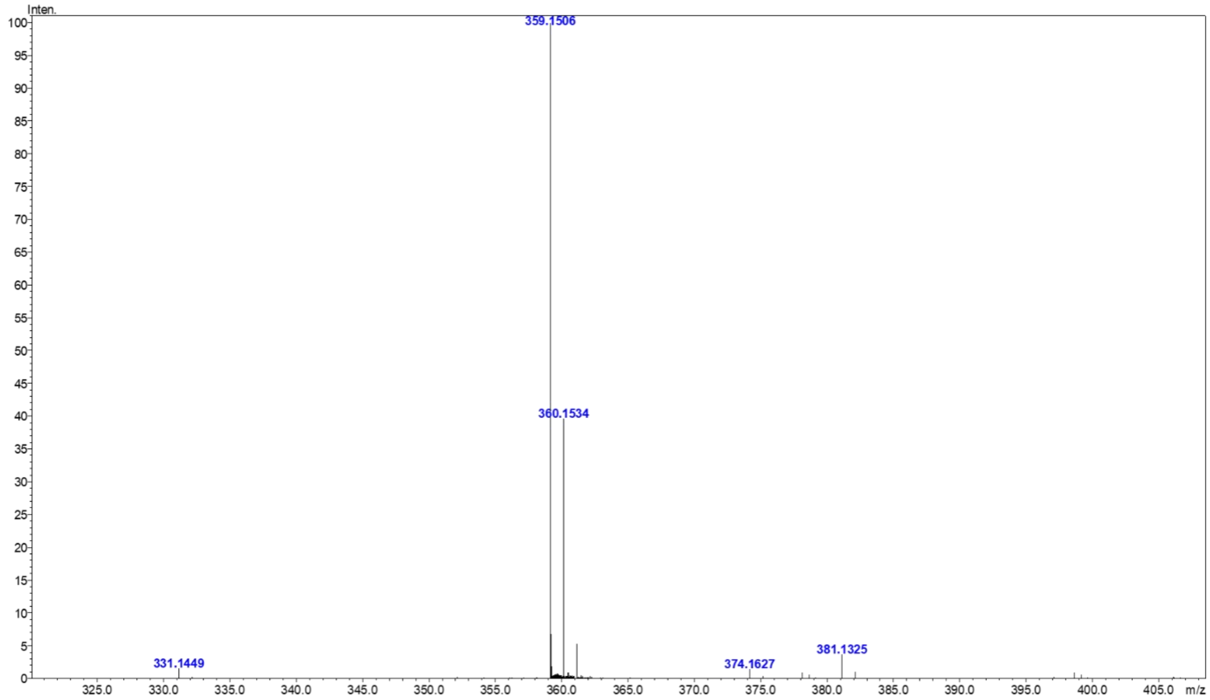

Figure S3. HRMS (ESI+) spectrum of (*3E,5E*)-1-(2-azidoacetyl)-3,5-bis(benzylidene)-piperidin-4-one (**16**).

*2.2 Figure S4-S7. Spectra of (3E,5E)-1-(2-azidoacetyl)-3,5-bis(4-fluorobenzylidene)-piperidin-4-one (**17**)*

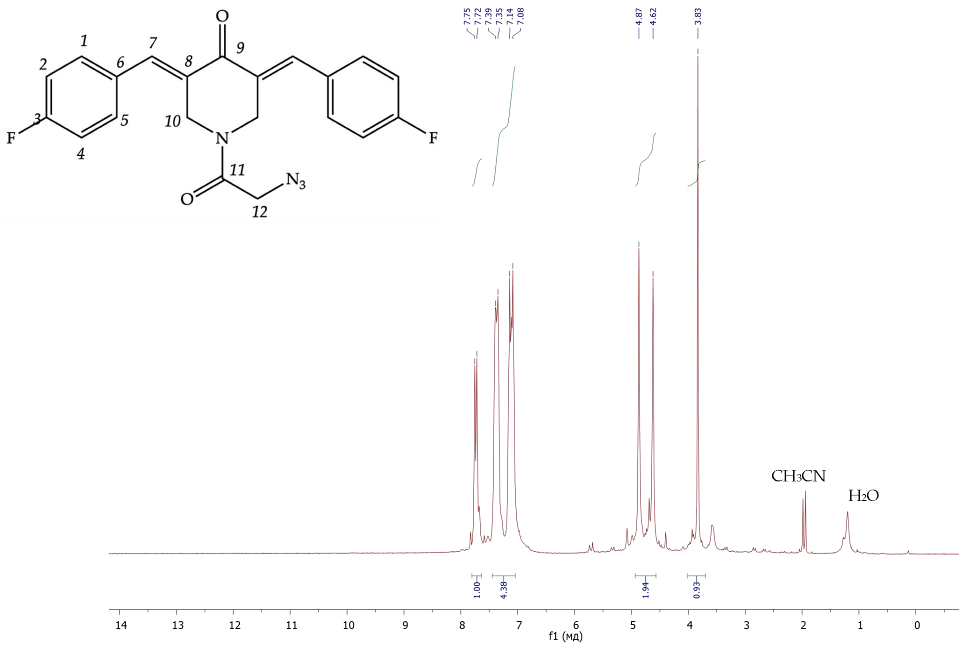

Figure S4. ^{1}H NMR spectrum of (*3E,5E*)-1-(2-azidoacetyl)-3,5-bis(4-fluorobenzylidene)-piperidin-4-one (**17**).

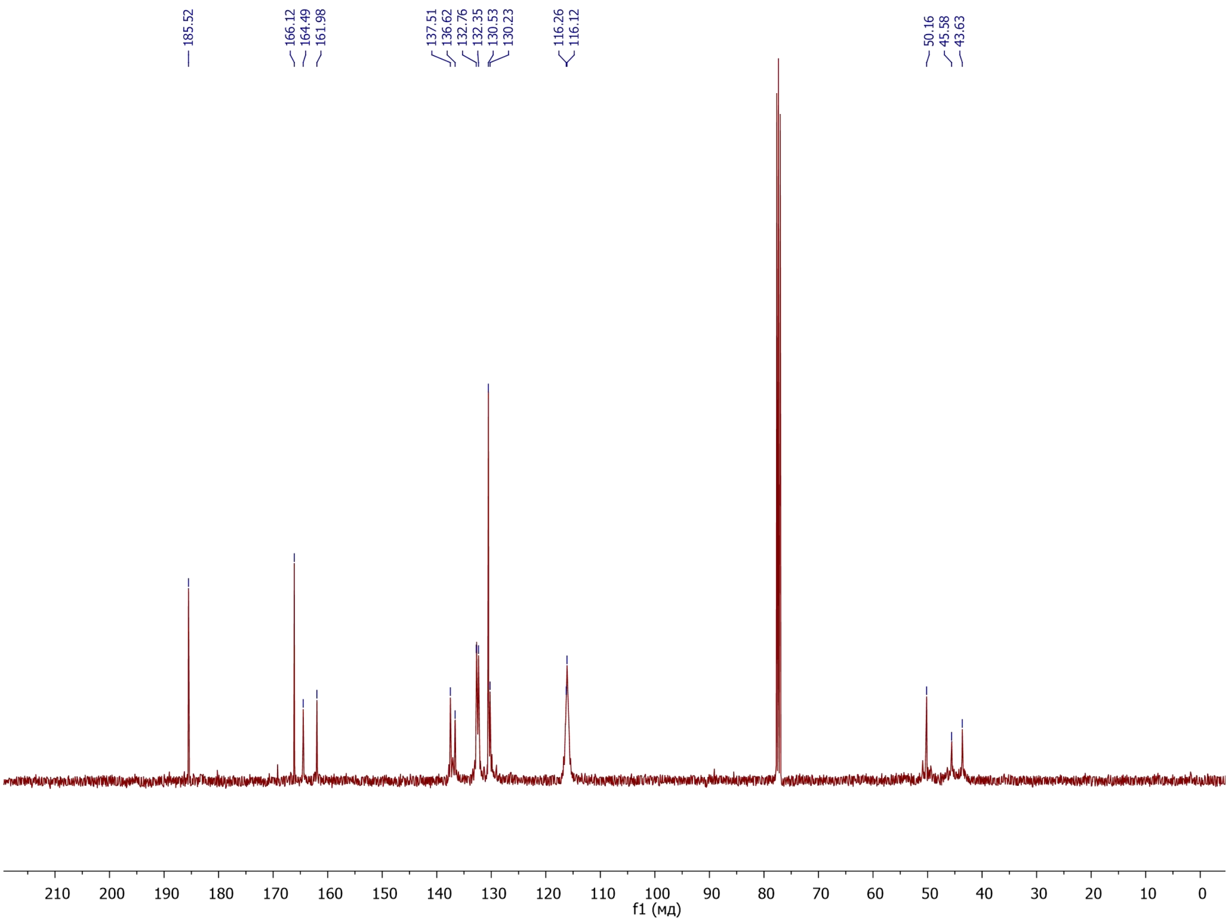

Figure S5. ^{13}C NMR spectrum of (*3E,5E*)-1-(2-azidoacetyl)-3,5-bis(4-fluorobenzylidene)-piperidin-4-one (**17**).

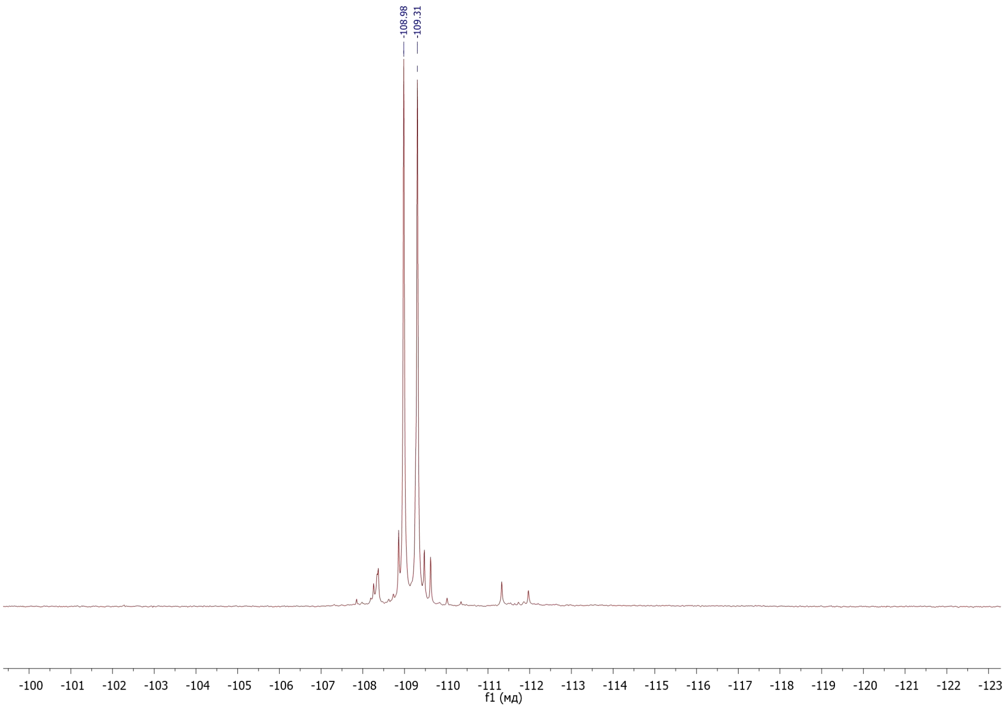

Figure S6. ^{19}F NMR spectrum of (*3E,5E*)-1-(2-azidoacetyl)-3,5-bis(4-fluorobenzylidene)-piperidin-4-one (**17**).

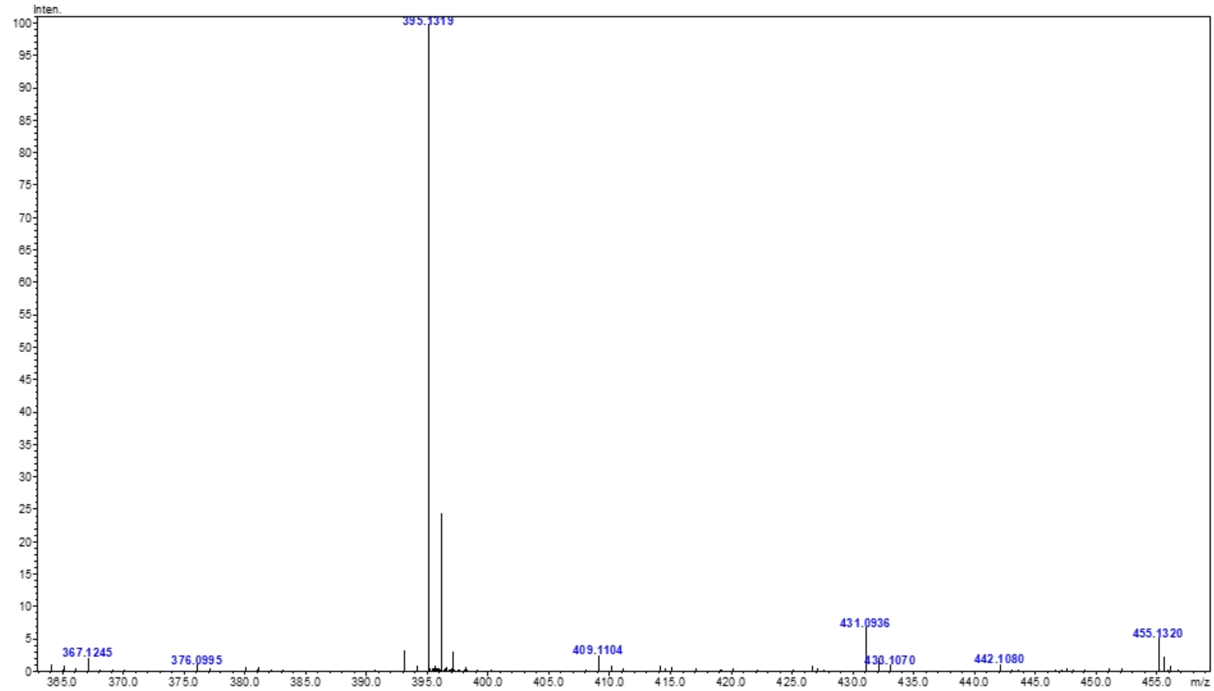

Figure S7. HRMS (ESI+) spectrum of (*3E,5E*)-1-(2-azidoacetyl)-3,5-bis(4-fluorobenzylidene)-piperidin-4-one (**17**).

*2.3 Figure S8-S10. Spectra of (3E,5E)-1-(2-azidoacetyl)-3,5-bis(4-chlorobenzylidene)-piperidin-4-one (**18**)*

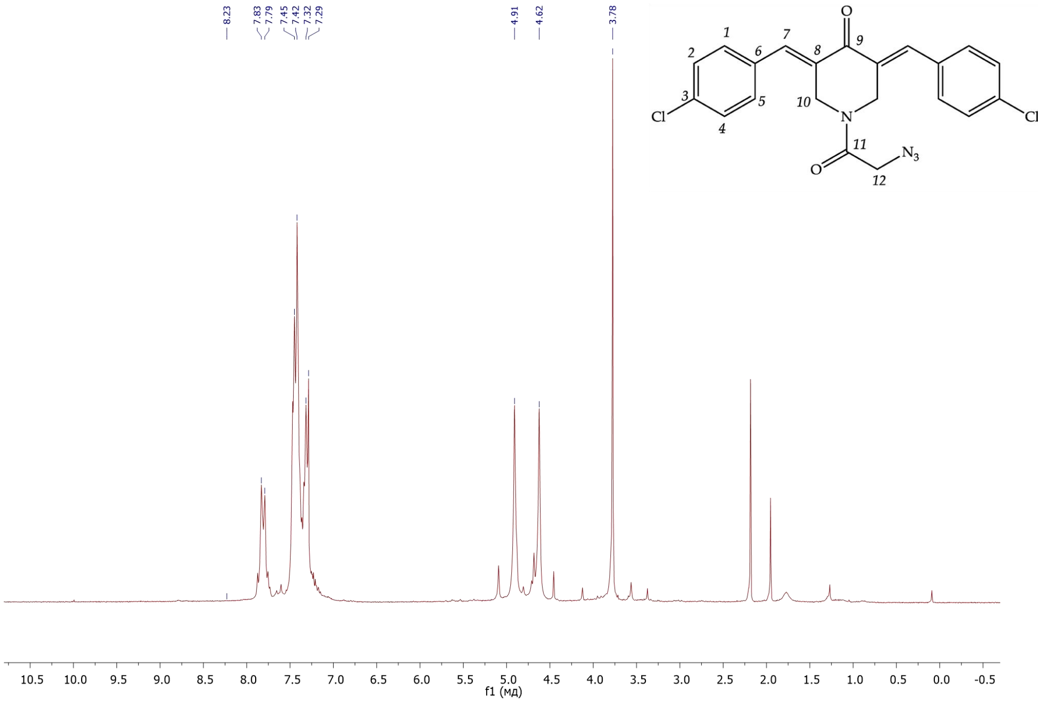

Figure S8. ^{1}H NMR spectrum of (*3E,5E*)-1-(2-azidoacetyl)-3,5-bis(4-chlorobenzylidene)-piperidin-4-one (**18**).

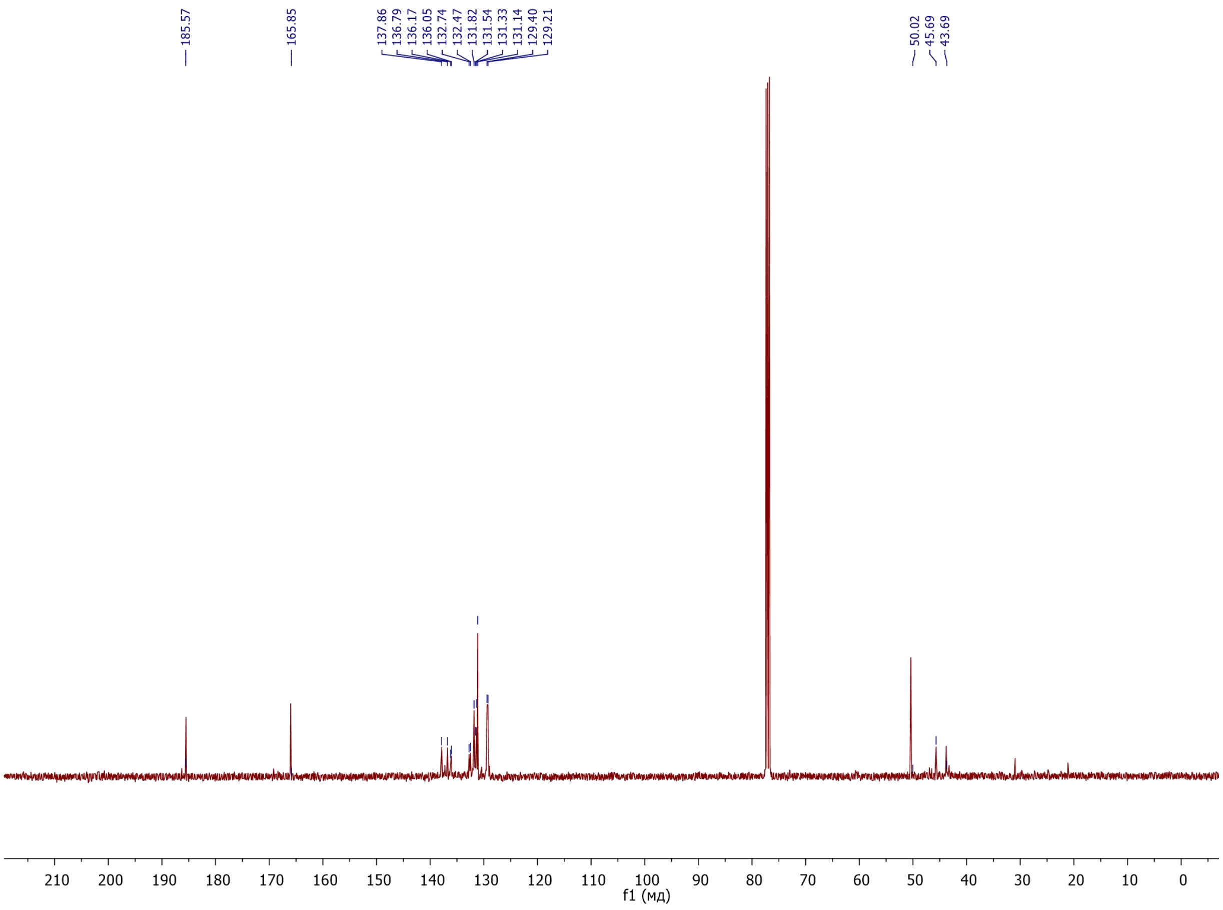

Figure S9. ^{13}C NMR spectrum of (*3E,5E*)-1-(2-azidoacetyl)-3,5-bis(4-chlorobenzylidene)-piperidin-4-one (**18**).

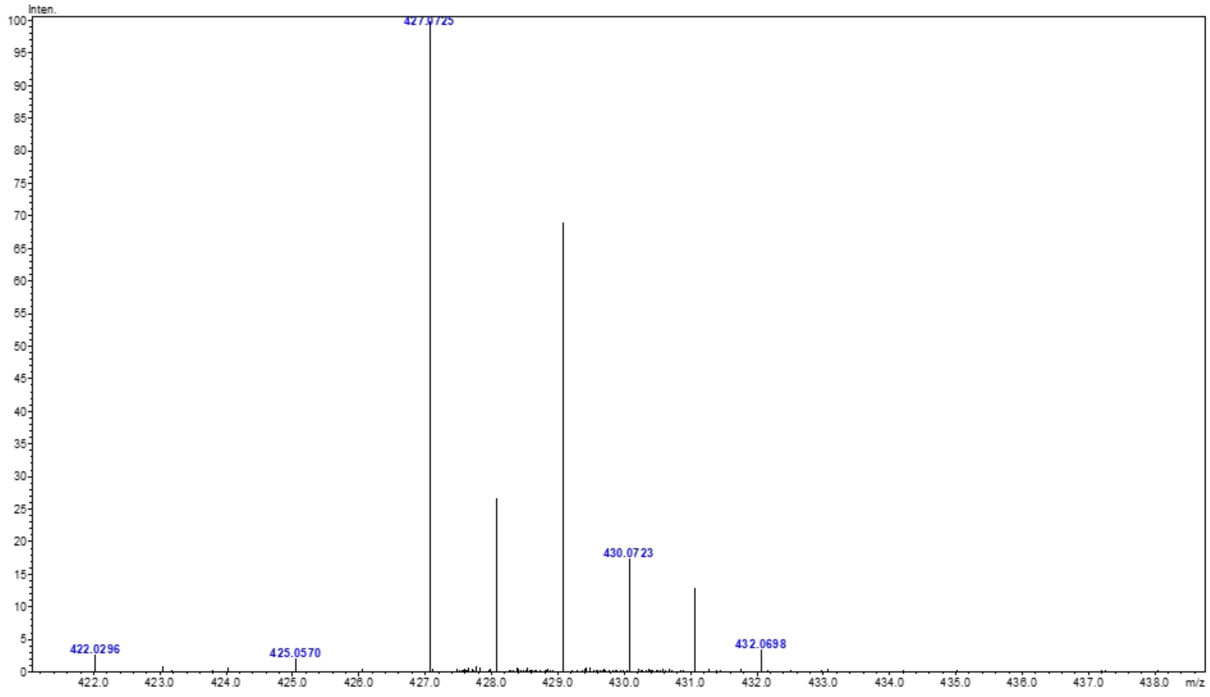

Figure S10. HRMS (ESI+) spectrum of (*3E,5E*)-1-(2-azidoacetyl)-3,5-bis(4-chlorobenzylidene)-piperidin-4-one (**18**).

*2.4 Figure S11-S13. Spectra of (3E,5E)-1-(2-azidoacetyl)-3,5-bis(4-bromobenzylidene)-piperidin-4-one (**19**)*

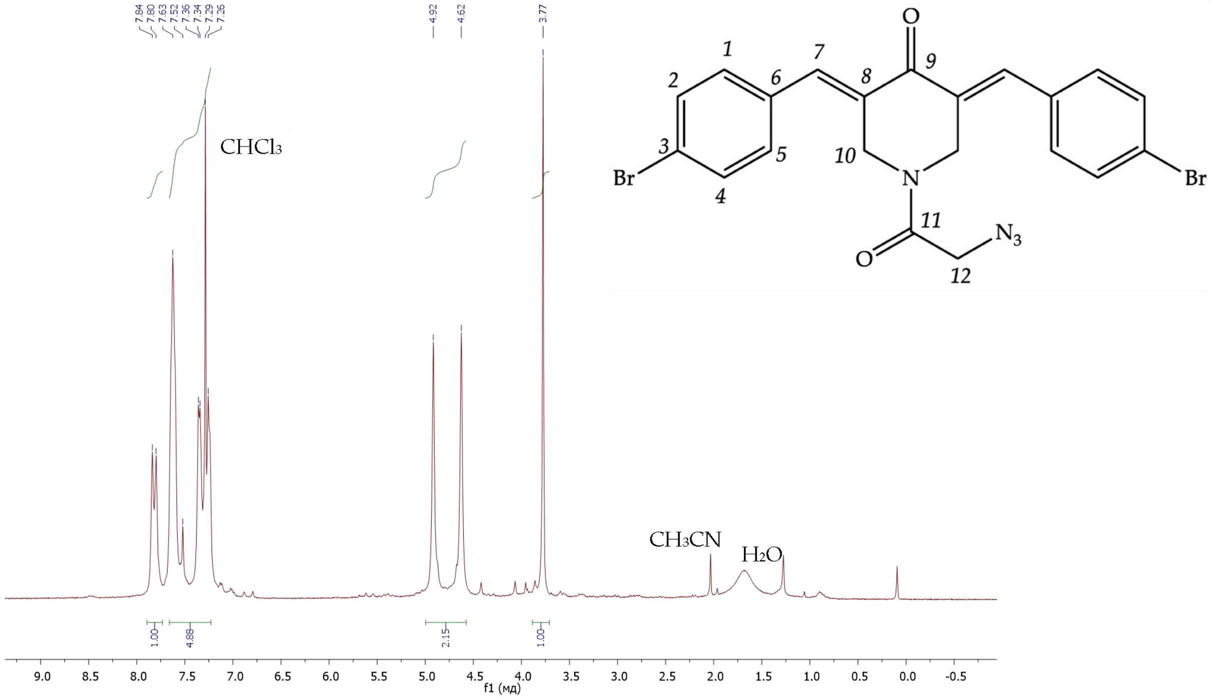

Figure S11. ^{1}H NMR spectrum of (3E,5E)-1-(2-azidoacetyl)-3,5-bis(4-bromobenzylidene)-piperidin-4-one (**19**).

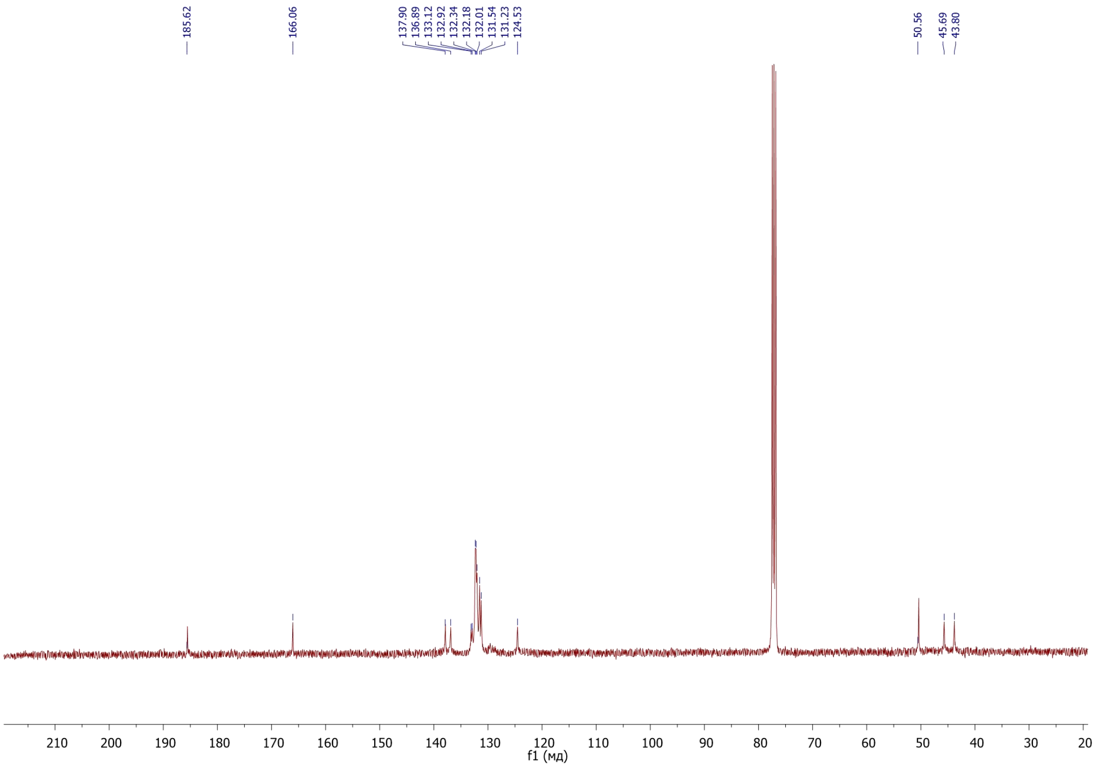

Figure S12. ^{13}C NMR spectrum of (*3E,5E*)-1-(2-azidoacetyl)-3,5-bis(4-bromobenzylidene)-piperidin-4-one (**19**).

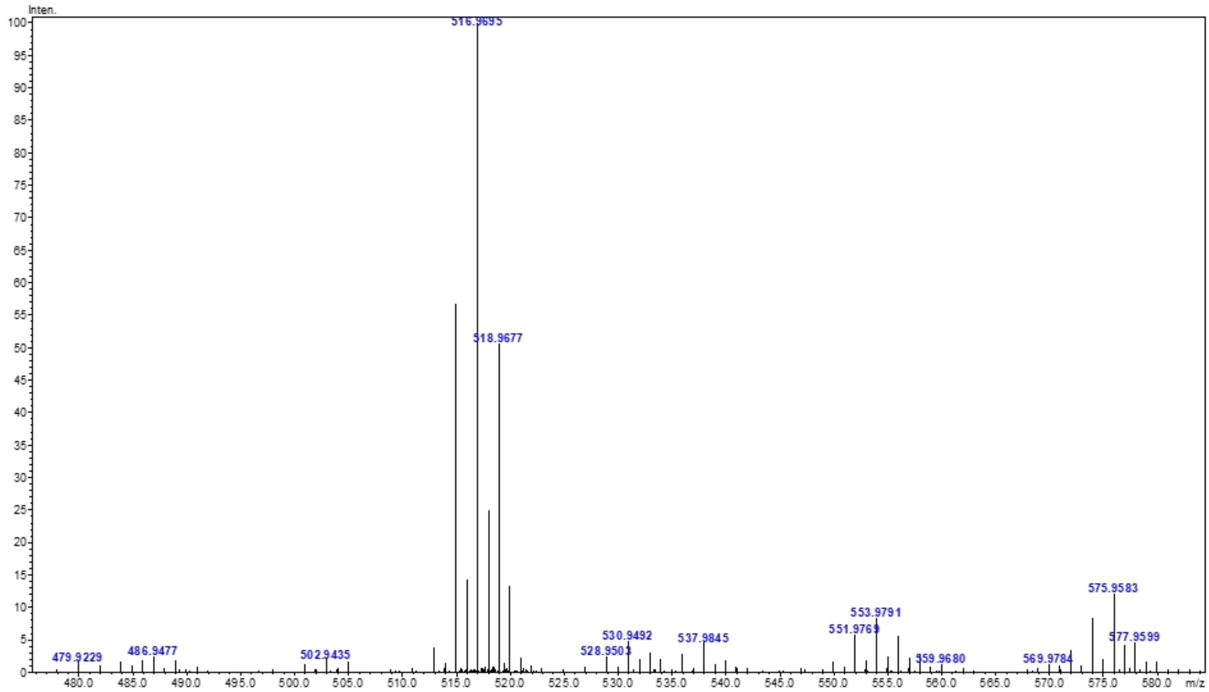

Figure S13. HRMS (ESI+) spectrum of (*3E,5E*)-1-(2-azidoacetyl)-3,5-bis(4-bromobenzylidene)-piperidin-4-one (**19**).

*2.5 Figure S14-S16. Spectra of (3E,5E)-1-(2-azidoacetyl)-3,5-bis(4-methoxybenzylidene)-piperidin-4-one (**20**)*

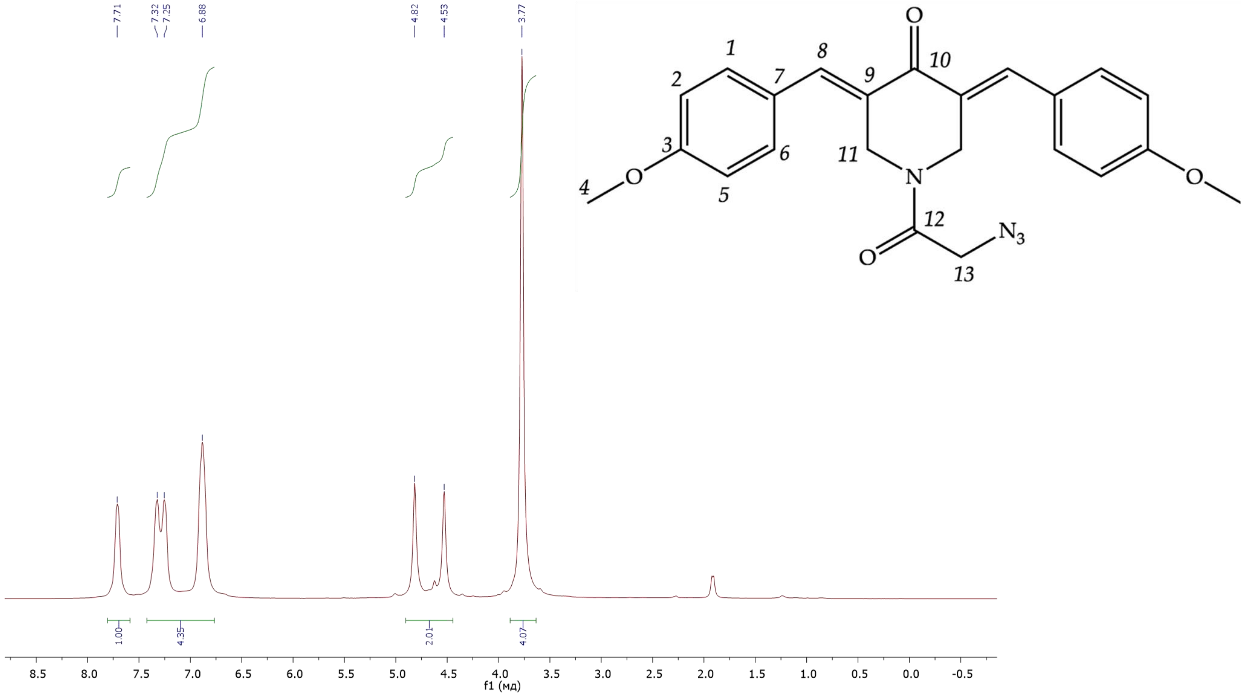

Figure S14. ^{1}H NMR spectrum of (*3E,5E*)-1-(2-azidoacetyl)-3,5-bis(4-methoxybenzylidene)-piperidin-4-one (**20**).

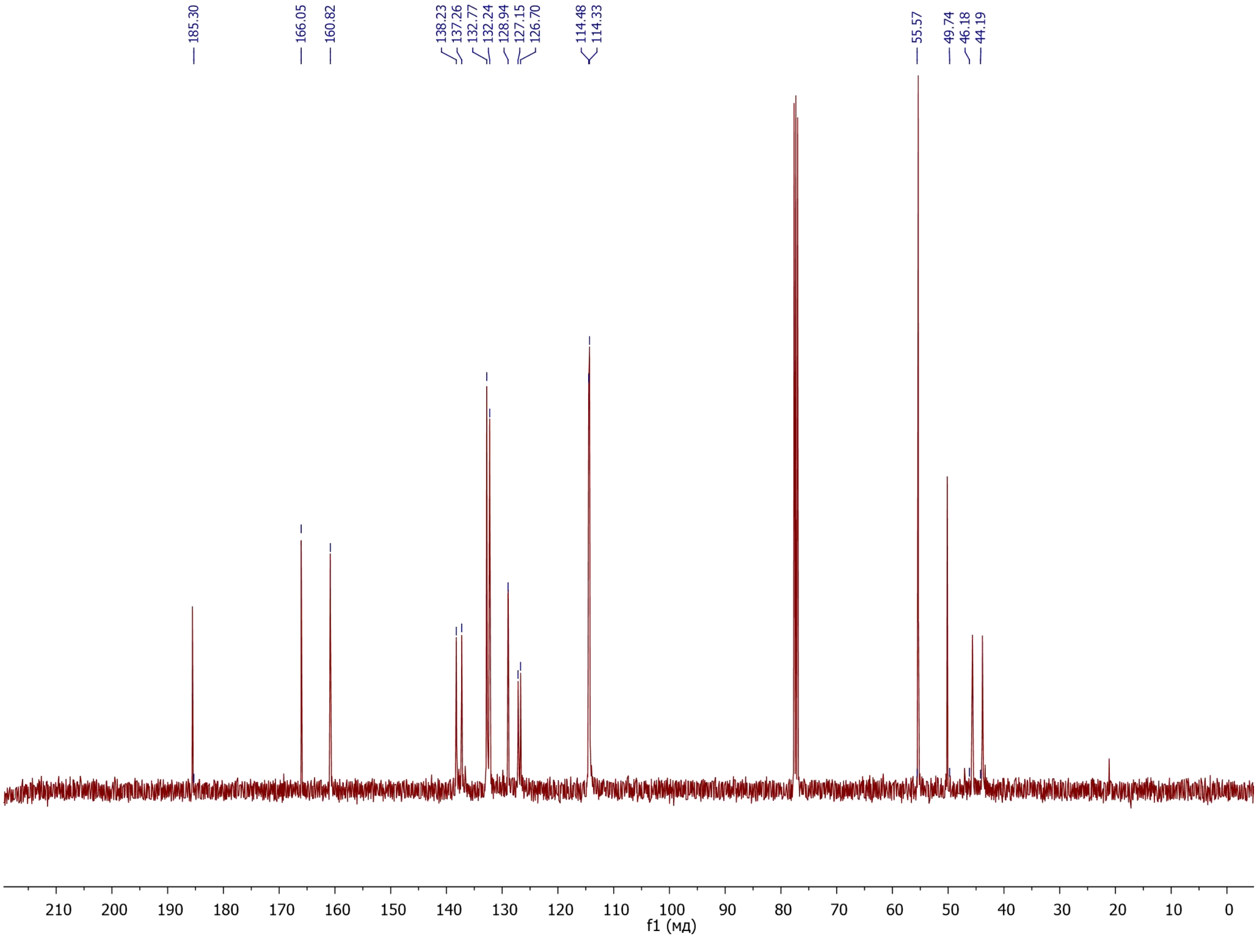

Figure S15. ^{13}C NMR spectrum of (*3E,5E*)-1-(2-azidoacetyl)-3,5-bis(4-methoxybenzylidene)-piperidin-4-one (**20**).

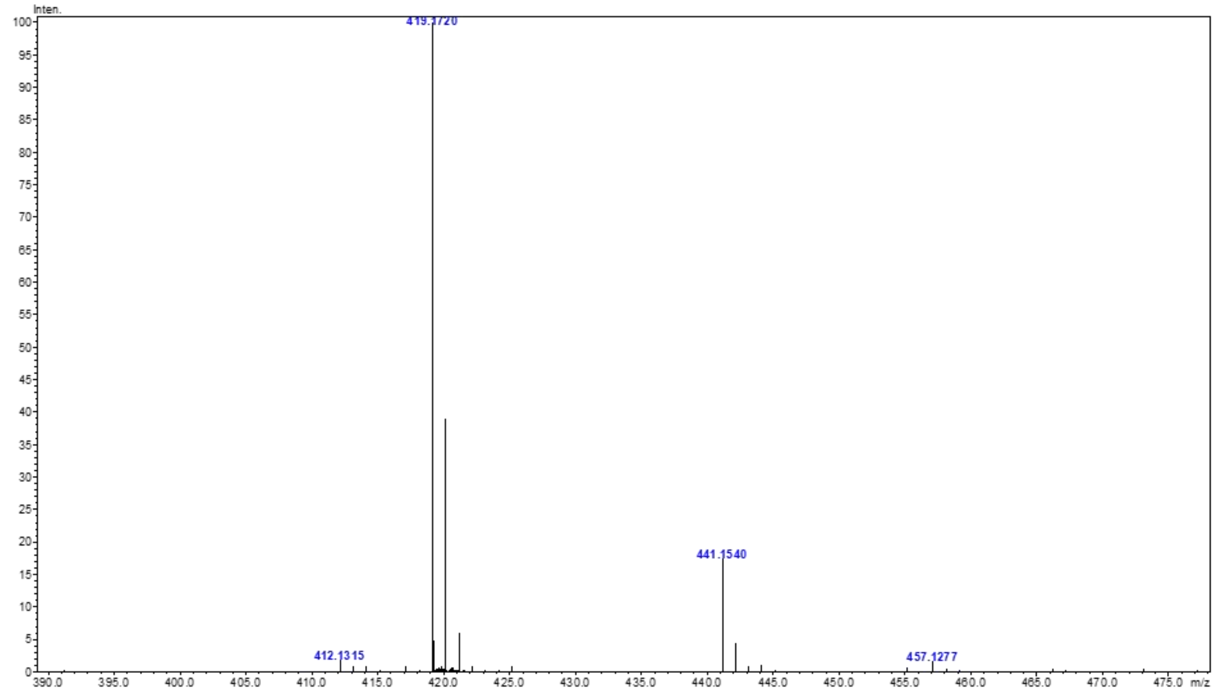

Figure S16. HRMS (ESI+) spectrum of (*3E,5E*)-1-(2-azidoacetyl)-3,5-bis(4-methoxybenzylidene)-piperidin-4-one (**20**).

*2.6 Figure S17-S19. Spectra of (3E,5E)-1-(2-azidoacetyl)-3,5-bis(4-isopropylbenzylidene)-piperidin-4-one (**21**)*

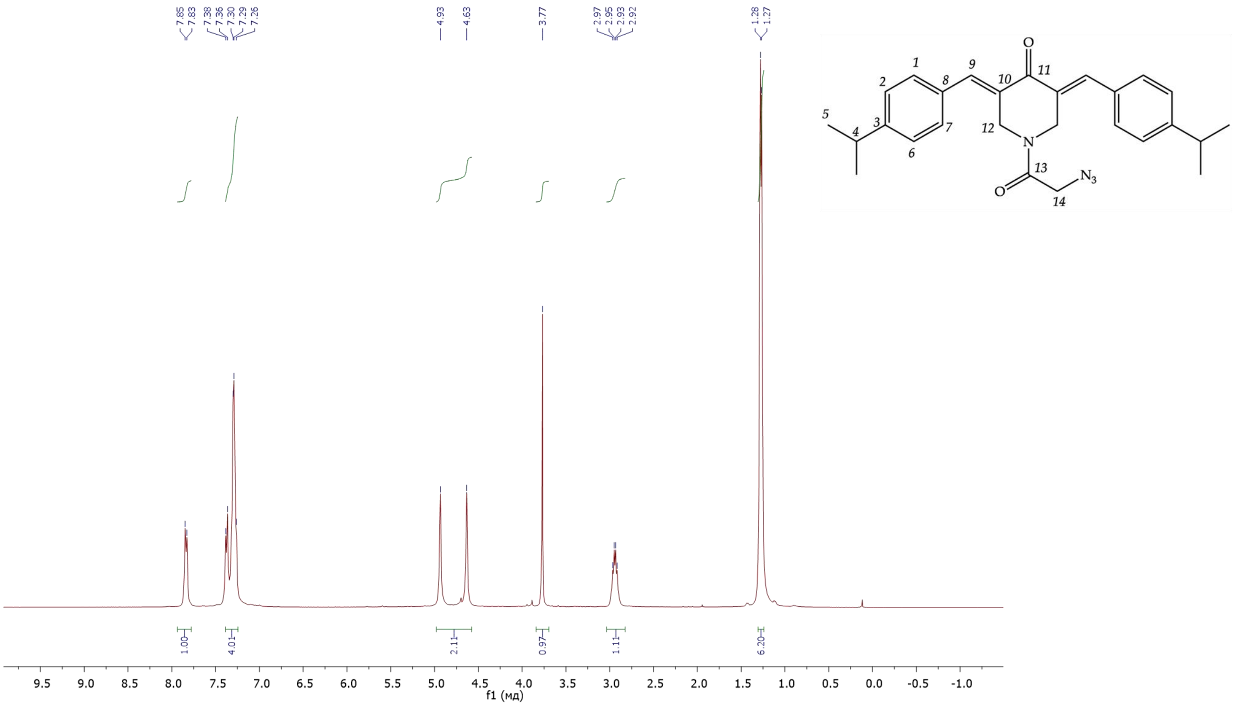

Figure S17. ^{1}H NMR spectrum of (*3E,5E*)-1-(2-azidoacetyl)-3,5-bis(4-isopropylbenzylidene)-piperidin-4-one (**21**).

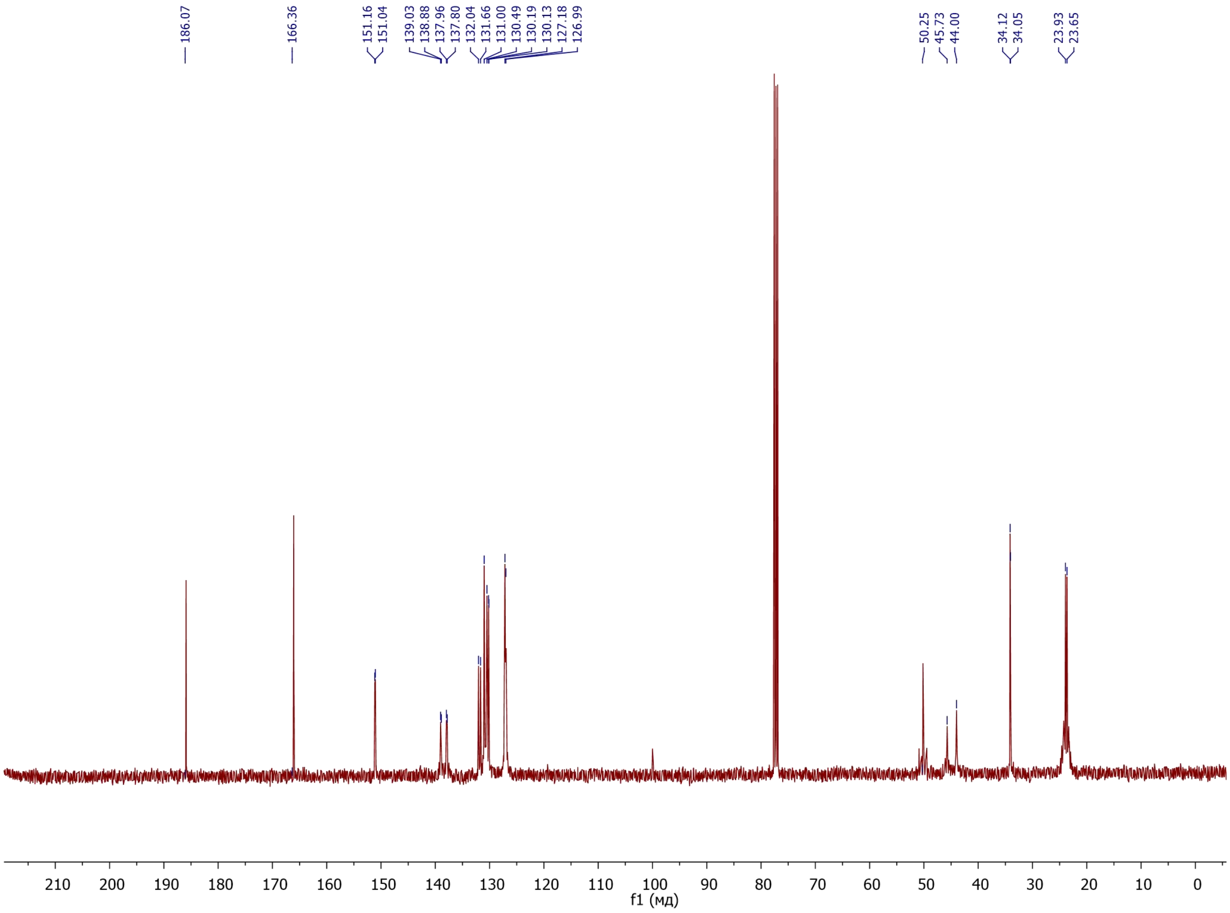

Figure S18. ^{13}C NMR spectrum of (*3E,5E*)-1-(2-azidoacetyl)-3,5-bis(4-isopropylbenzylidene)-piperidin-4-one (**21**).

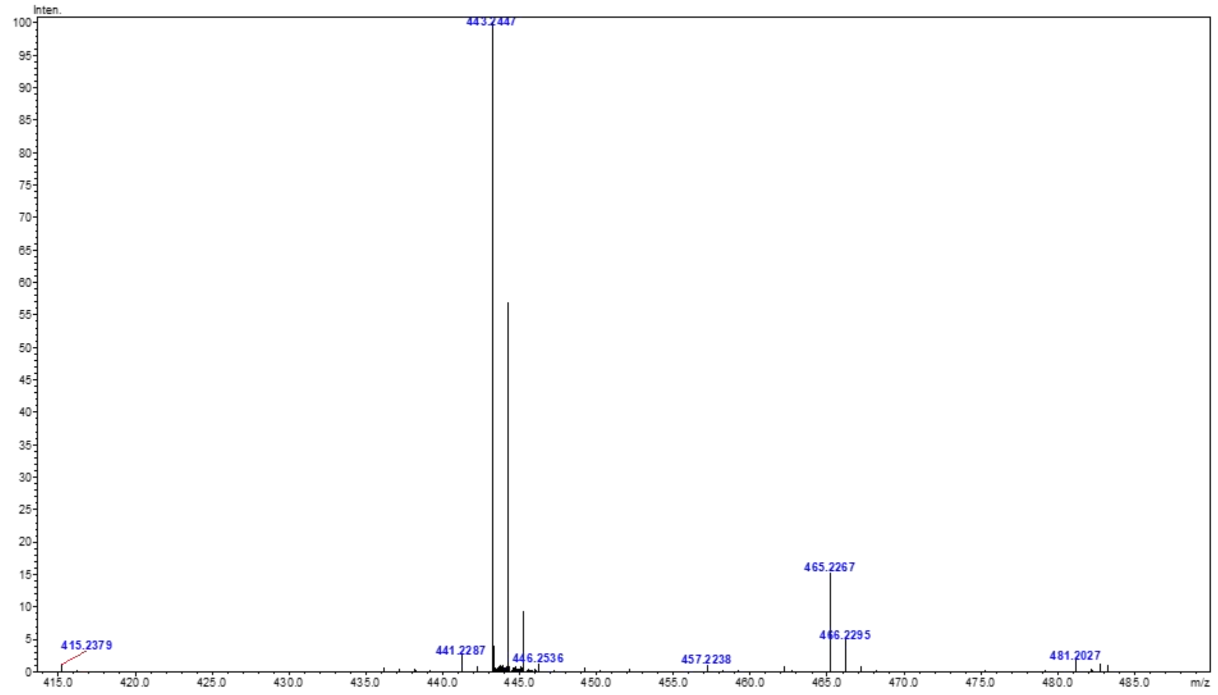

Figure S19. HRMS (ESI+) spectrum of (*3E,5E*)-1-(2-azidoacetyl)-3,5-bis(4-isopropylbenzylidene)-piperidin-4-one (**21**).

*2.7 Figure S20-S22. Spectra of (3E,5E)-1-(2-azidoacetyl)-3,5-bis(3,4,5-trimethoxybenzylidene)-piperidin-4-one (**22**)*

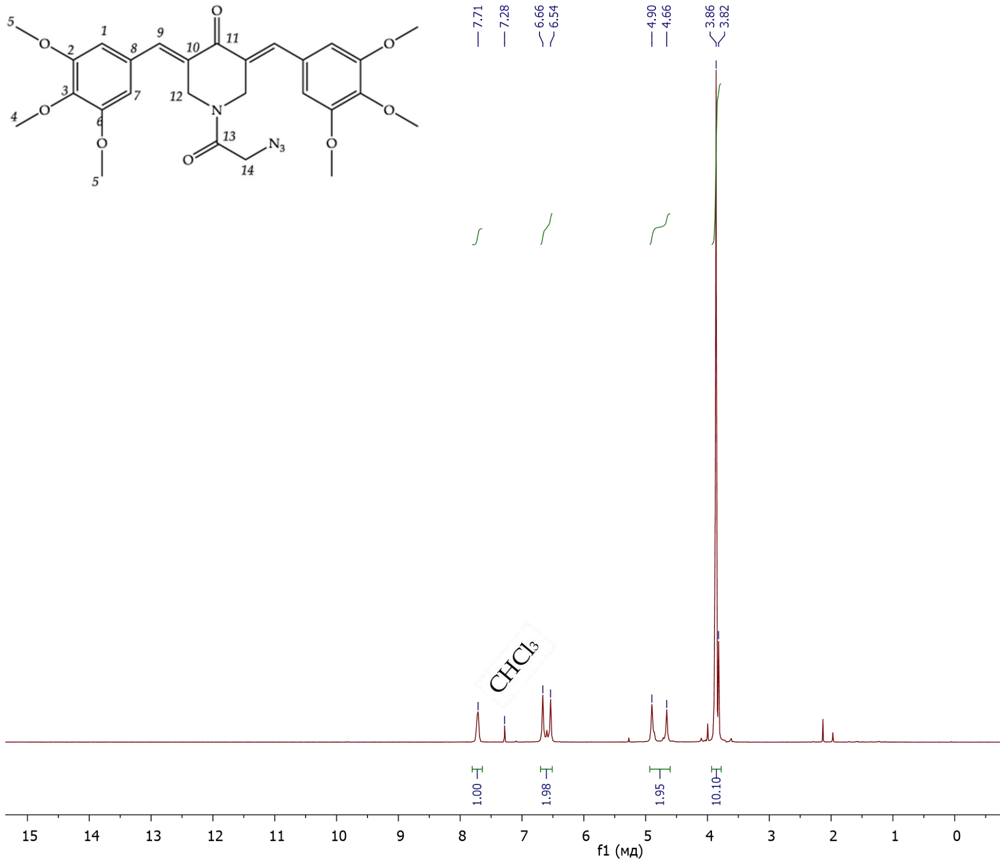

Figure S20. ^{1}H NMR spectrum of (*3E,5E*)-1-(2-azidoacetyl)-3,5-bis(3,4,5-trimethoxybenzylidene)-piperidin-4-one (**22**).

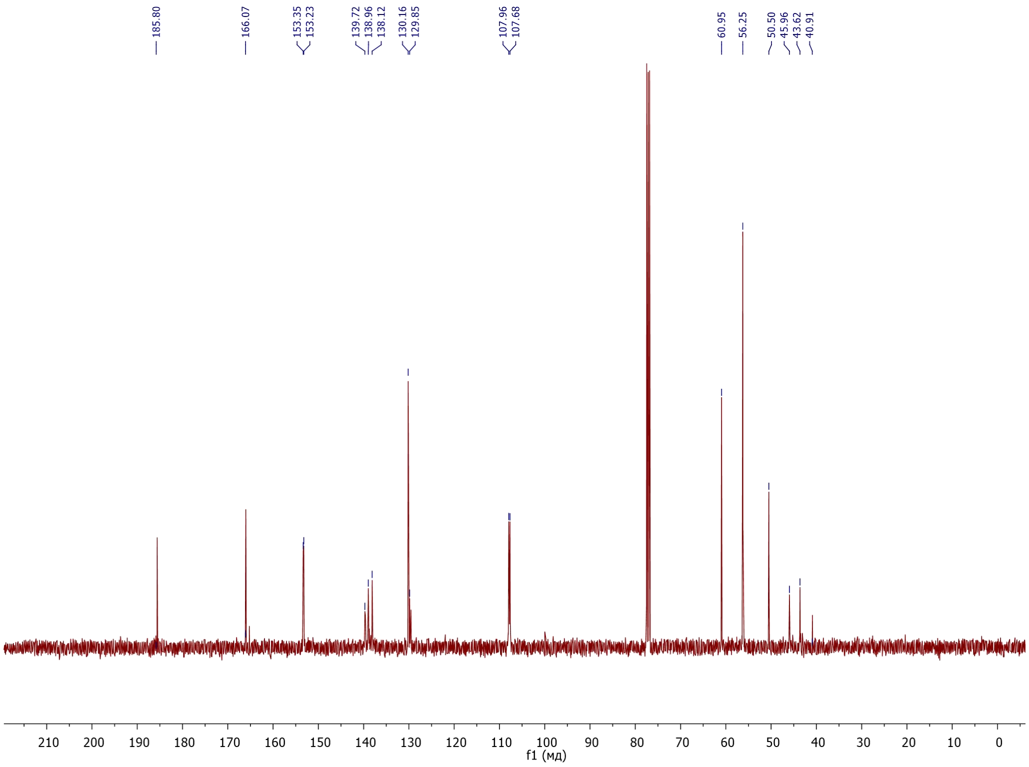

Figure S21. ^{13}C NMR spectrum of (*3E*,*5E*)-1-(2-azidoacetyl)-3,5-bis(3,4,5-trimethoxybenzylidene)-piperidin-4-one (**22**).

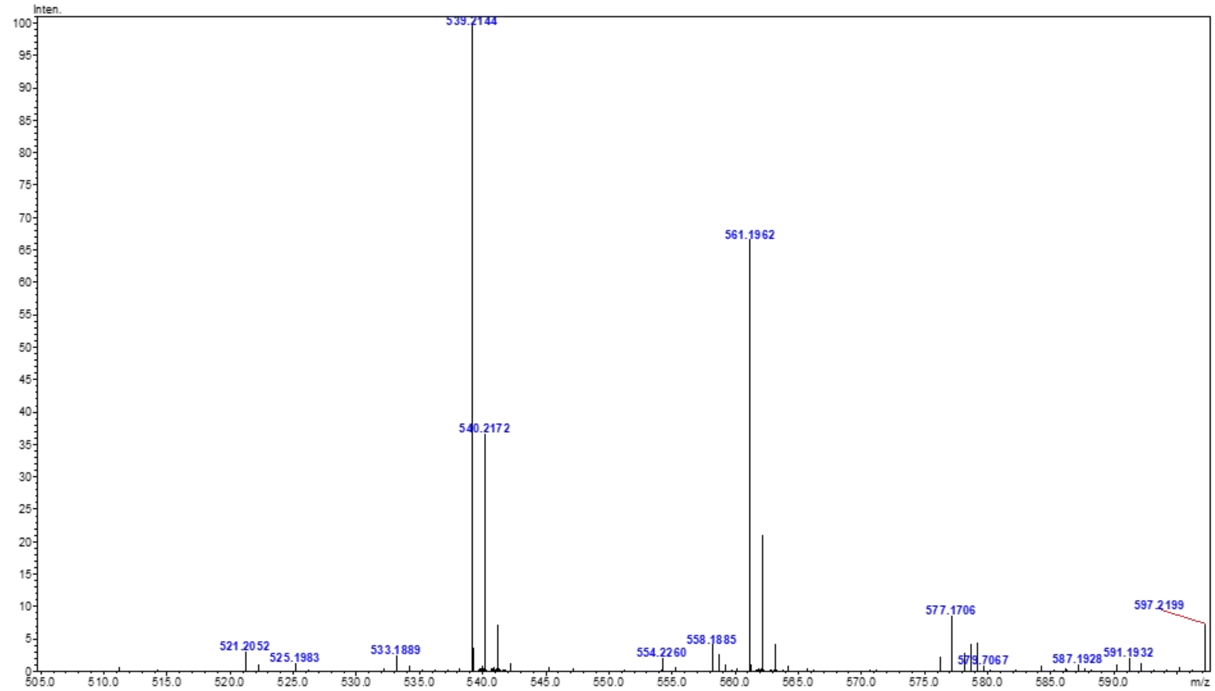

Figure S22. HRMS (ESI+) spectrum of (*3E*,*5E*)-1-(2-azidoacetyl)-3,5-bis(3,4,5-trimethoxybenzylidene)-piperidin-4-one (**22**).

3. *Figure S23-S43. NMR and HRMS (ESI+) spectra of (3E,5E)-1-(2-(4-((10H-phenothiazine-10-yl)methyl)-1H-1,2,3-triazol-1-yl)acetyl)-3,5-bis(benzylidene)-piperidin-4-ones (**23-29**)*

3.1 *Figure S23-S25. Spectra of (3E,5E)-1-(2-(4-((10H-phenothiazine-10-yl)methyl)-1H-1,2,3-triazol-1-yl)acetyl)-3,5-bis(benzylidene)-piperidin-4-one (**23**)*

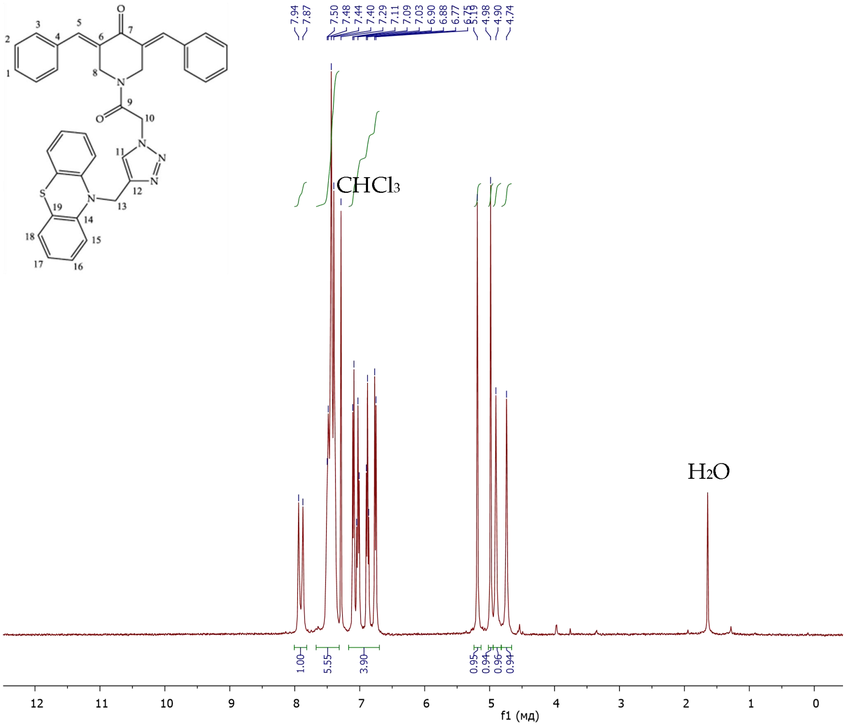

Figure S23. ^{1}H NMR spectrum of (*3E,5E*)-1-(2-(4-((10H-phenothiazine-10-yl)methyl)-1H-1,2,3-triazol-1-yl)acetyl)-3,5-bis(benzylidene)-piperidin-4-one (**23**).

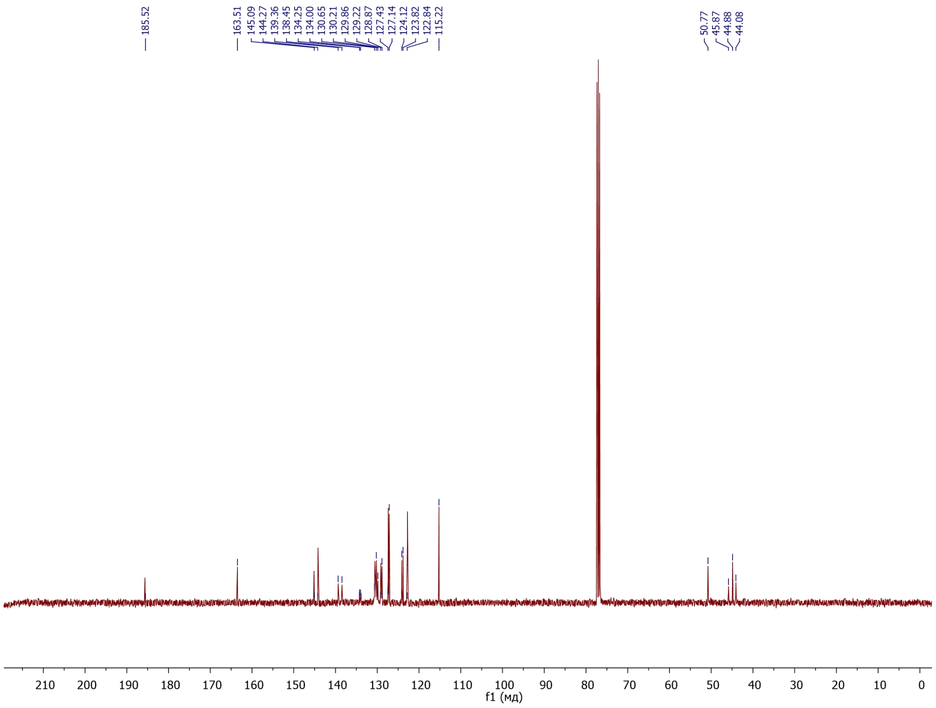

Figure S24. ^{13}C NMR spectrum of (*3E,5E*)-1-(2-(4-((10H-phenothiazine-10-yl)methyl)-1H-1,2,3-triazol-1-yl)acetyl)-3,5-bis(benzylidene)-piperidin-4-one (**23**).

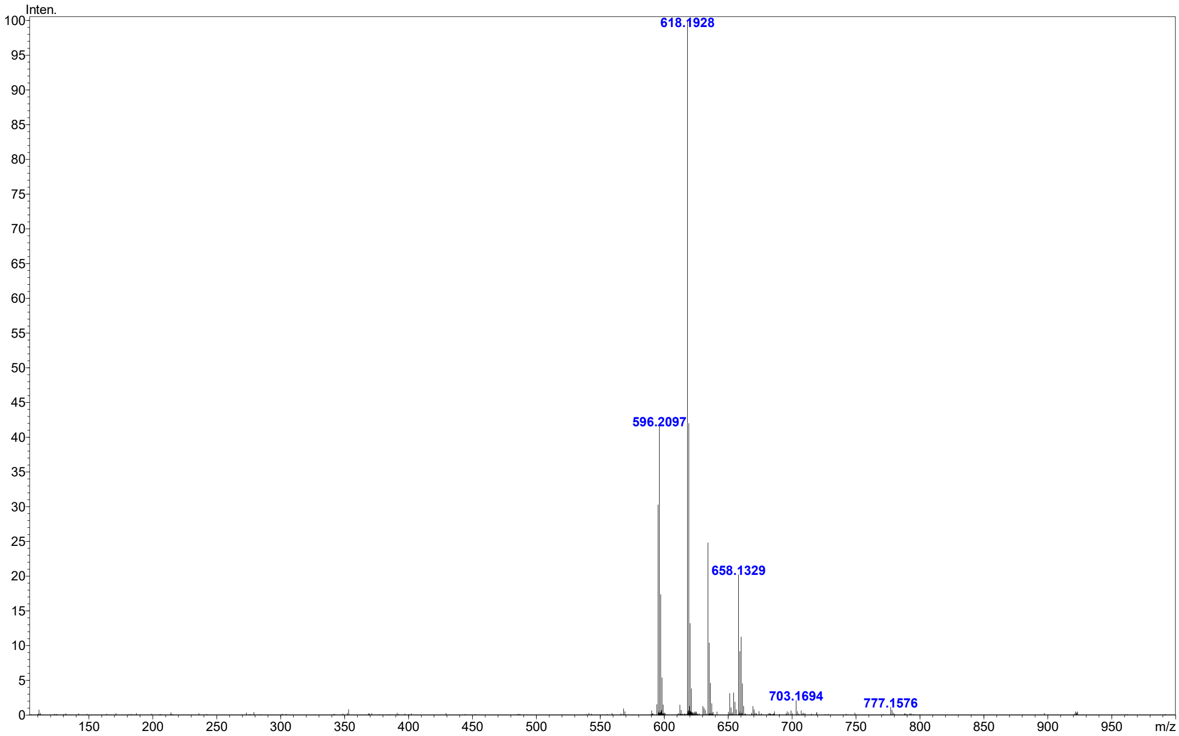

Figure S25. HRMS (ESI+) spectrum of (*3E,5E*)-1-(2-(4-((10H-phenothiazine-10-yl)methyl)-1H-1,2,3-triazol-1-yl)acetyl)-3,5-bis(benzylidene)-piperidin-4-one (**23**).

*3.2 Figure S26-S29. Spectra of (3E,5E)-1-(2-(4-((10H-phenothiazine-10-yl)methyl)-1H-1,2,3-triazol-1-yl)acetyl)-3,5-bis(4-fluorobenzylidene)-piperidin-4-one (**24**)*

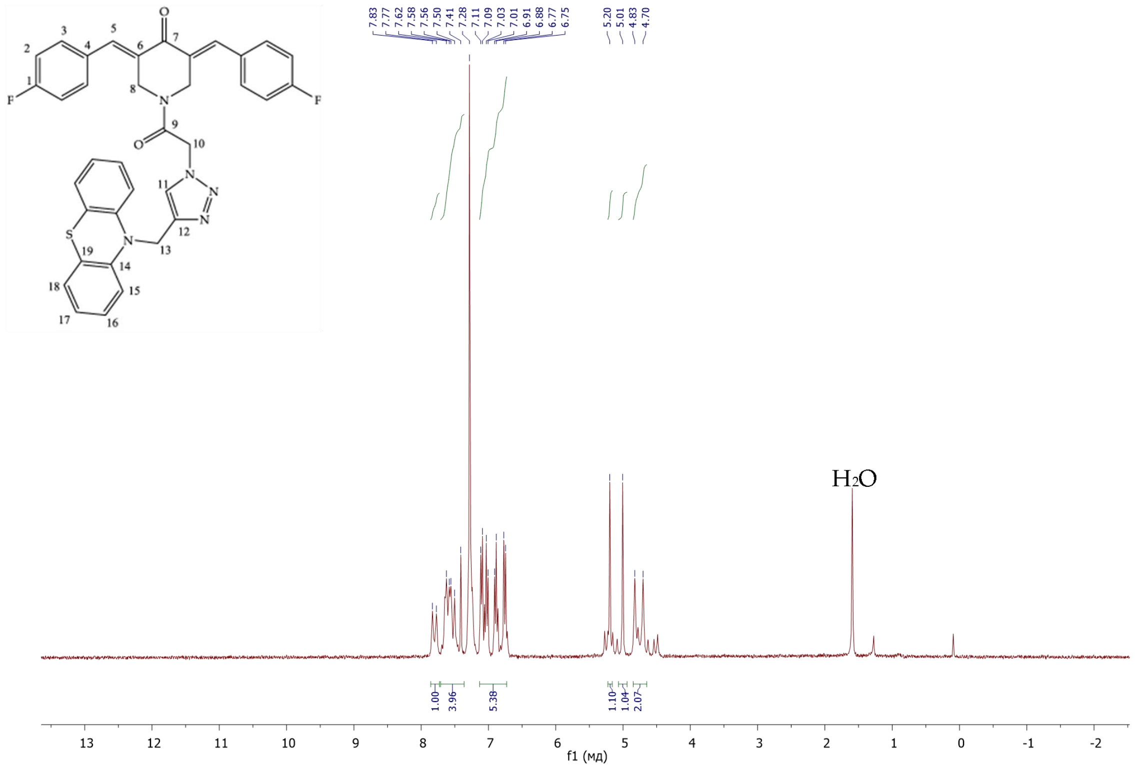

Figure S26. ^{1}H NMR spectrum of (*3E,5E*)-1-(2-(4-((10H-phenothiazine-10-yl)methyl)-1H-1,2,3-triazol-1-yl)acetyl)-3,5-bis(4-fluorobenzylidene)-piperidin-4-one (**24**).

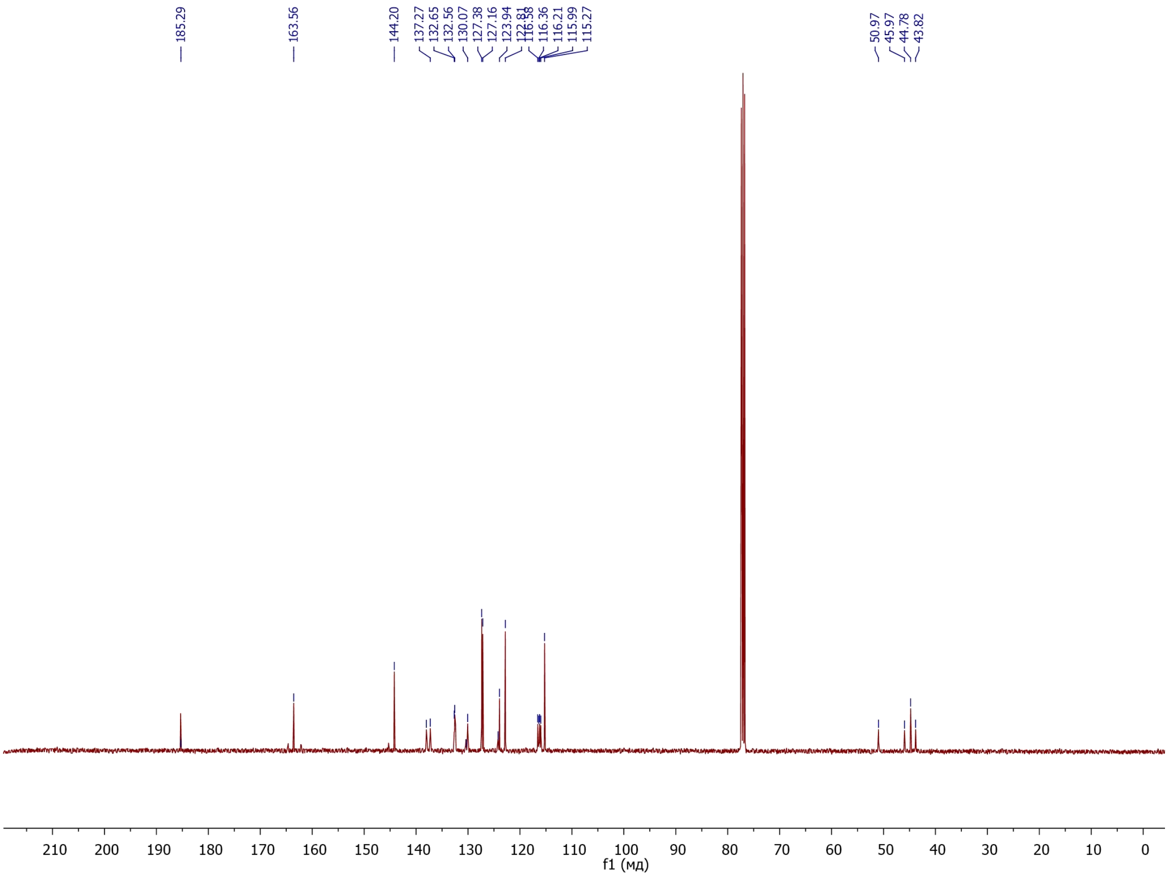

Figure S27. ^{13}C NMR spectrum of (*3E,5E*)-1-(2-(4-((10H-phenothiazine-10-yl)methyl)-1H-1,2,3-triazol-1-yl)acetyl)-3,5-bis(4-fluorobenzylidene)-piperidin-4-one (**24**).

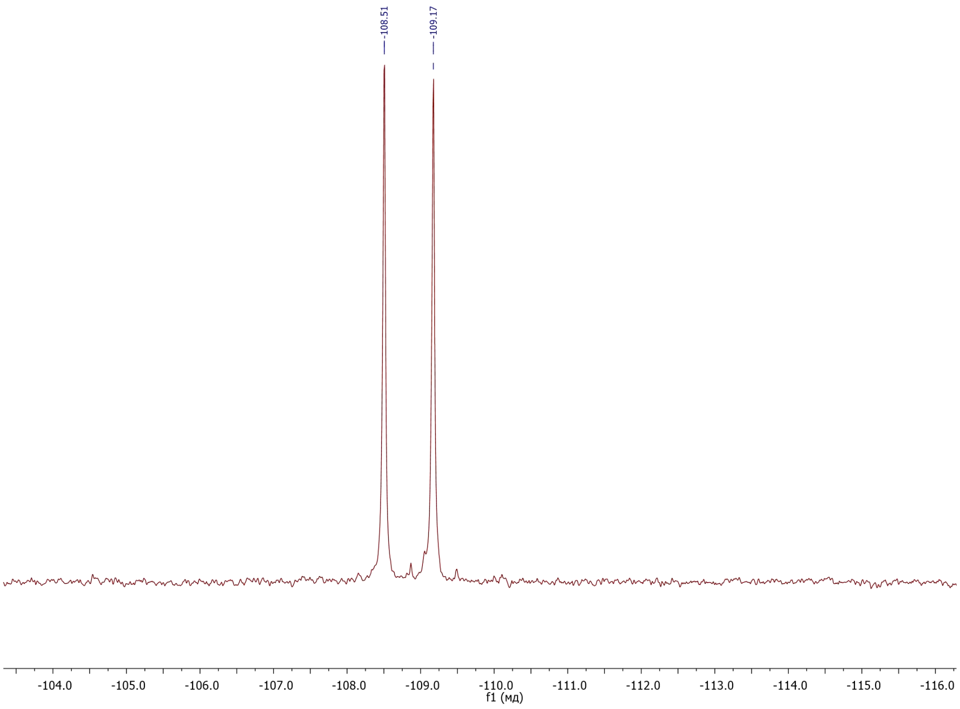

Figure S28. ^{19}F NMR spectrum of (*3E,5E*)-1-(2-(4-((10H-phenothiazine-10-yl)methyl)-1H-1,2,3-triazol-1-yl)acetyl)-3,5-bis(4-fluorobenzylidene)-piperidin-4-one (**24**).

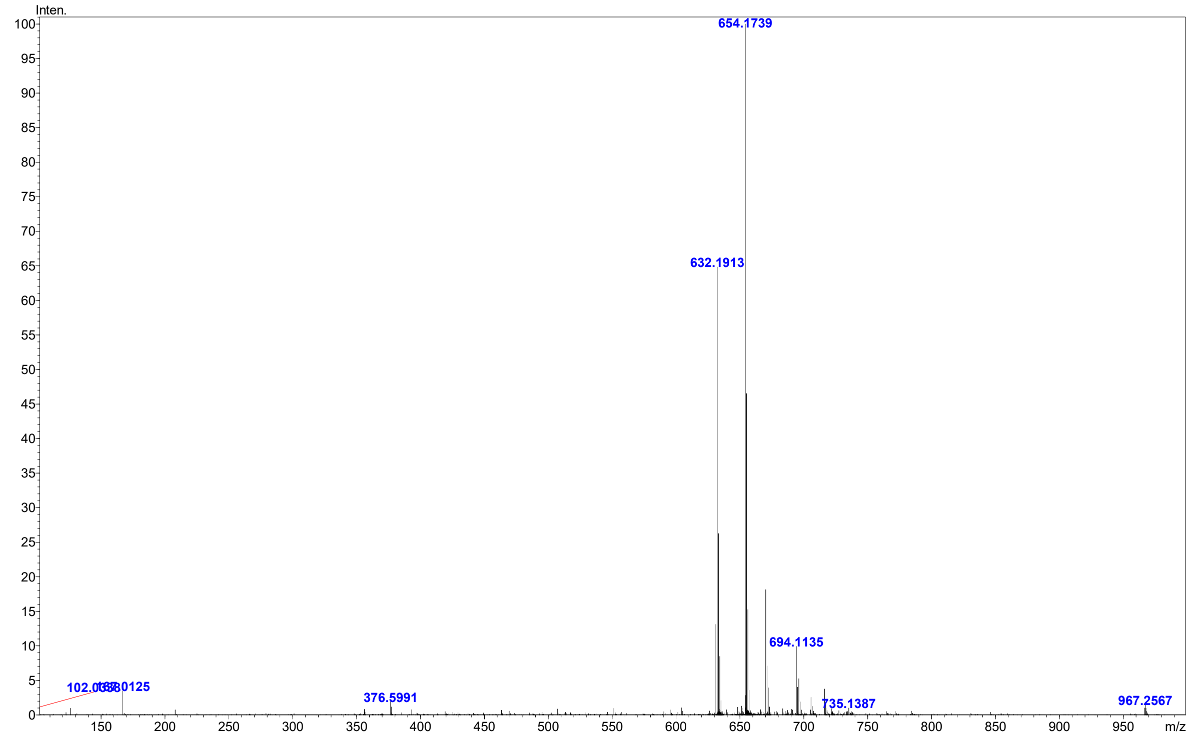

Figure S29. ESI-MS spectrum of (*3E,5E*)-1-(2-(4-((10H-phenothiazine-10-yl)methyl)-1H-1,2,3-triazol-1-yl)acetyl)-3,5-bis(4-fluorobenzylidene)-piperidin-4-one (**24**).

*3.3 Figure S30-S32. Spectra of (3E,5E)-1-(2-(4-((10H-phenothiazine-10-yl)methyl)-1H-1,2,3-triazol-1-yl)acetyl)-3,5-bis(4-chlorobenzylidene)-piperidin-4-one (**25**)*

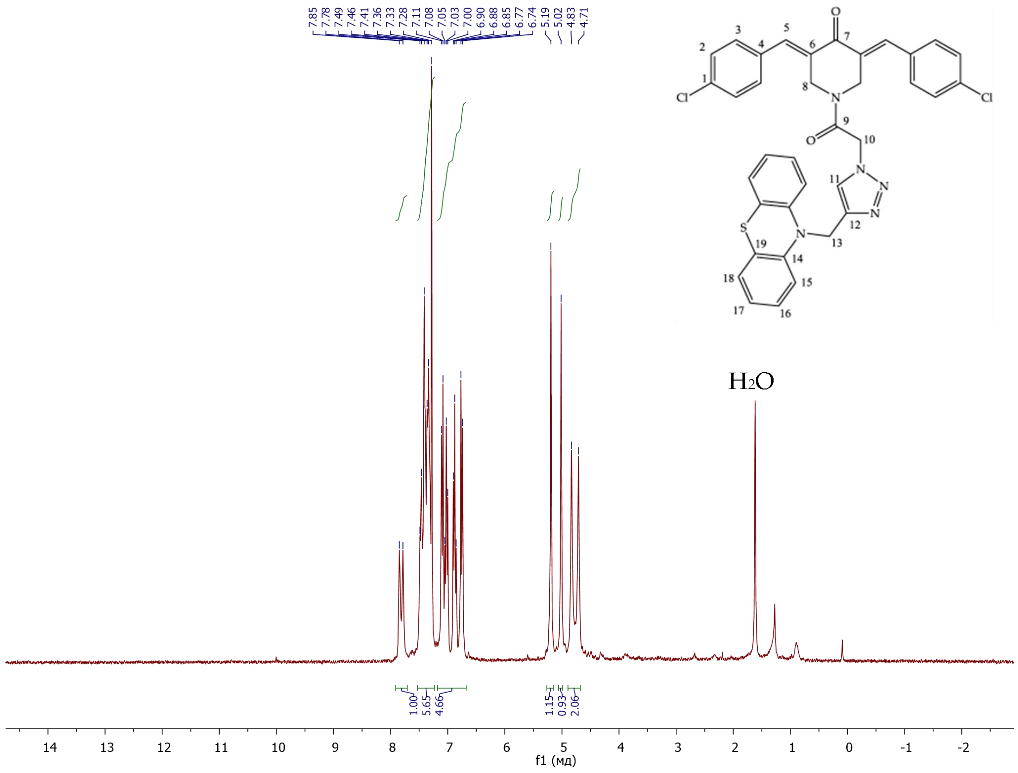

Figure S30. ^{1}H NMR spectrum of (*3E,5E*)-1-(2-(4-((10H-phenothiazine-10-yl)methyl)-1H-1,2,3-triazol-1-yl)acetyl)-3,5-bis(4-chlorobenzylidene)-piperidin-4-one (**25**).

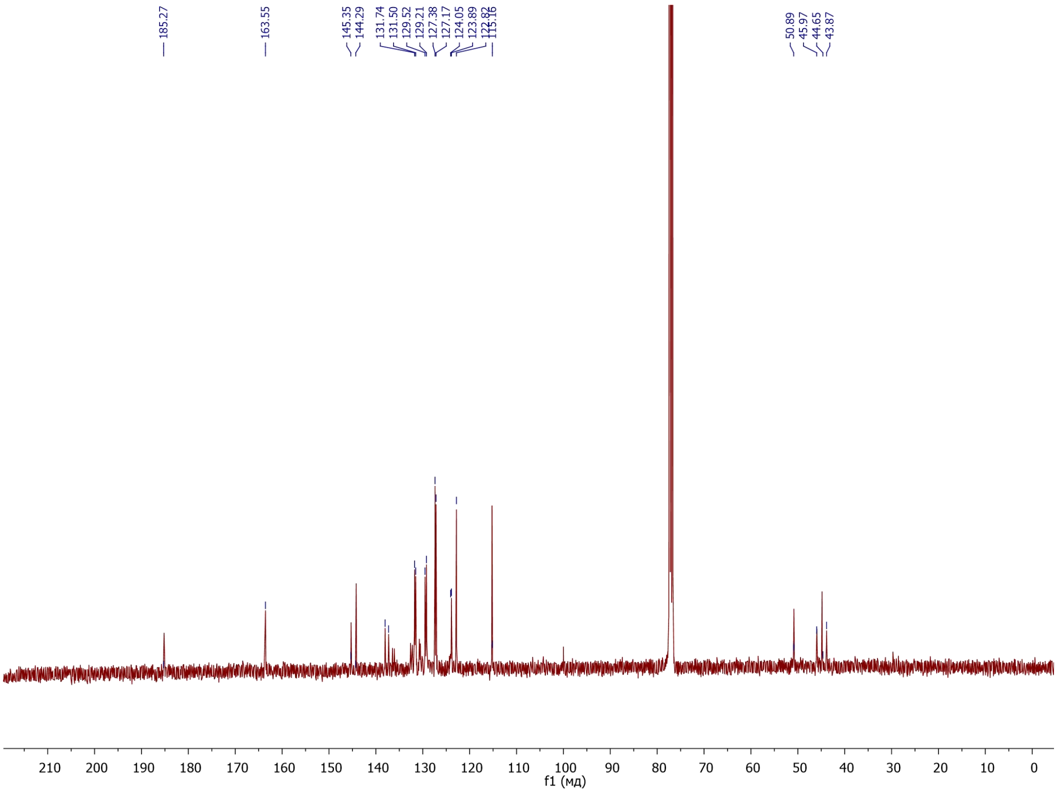

Figure S31. ^{13}C NMR spectrum of (*3E,5E*)-1-(2-(4-((10H-phenothiazin-10-yl)methyl)-1H-1,2,3-triazol-1-yl)acetyl)-3,5-bis(4-chlorobenzylidene)-piperidin-4-one (**25**).

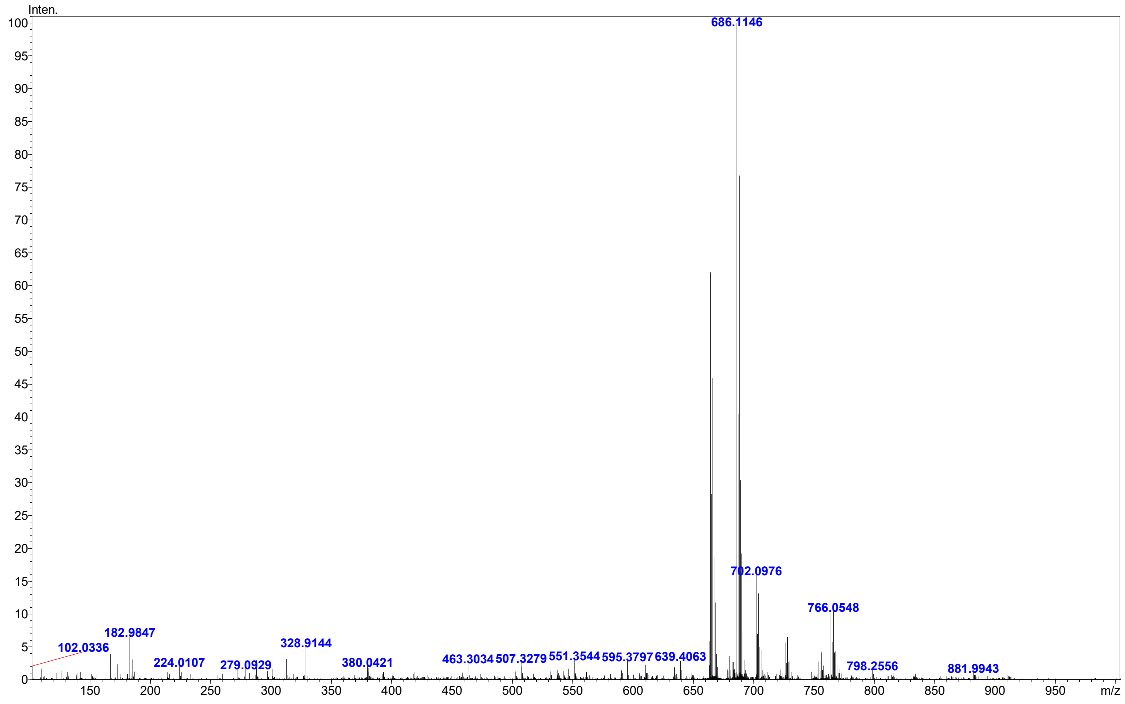

Figure S32. ESI-MS spectrum of (*3E,5E*)-1-(2-(4-((10H-phenothiazine-10-yl)methyl)-1H-1,2,3-triazol-1-yl)acetyl)-3,5-bis(4-chlorobenzylidene)-piperidin-4-one (**25**).

*3.4 Figure S33-S34. Spectra of (3E,5E)-1-(2-(4-((10H-phenothiazine-10-yl)methyl)-1H-1,2,3-triazol-1-yl)acetyl)-3,5-bis(4-bromobenzylidene)-piperidin-4-one (**26**)*

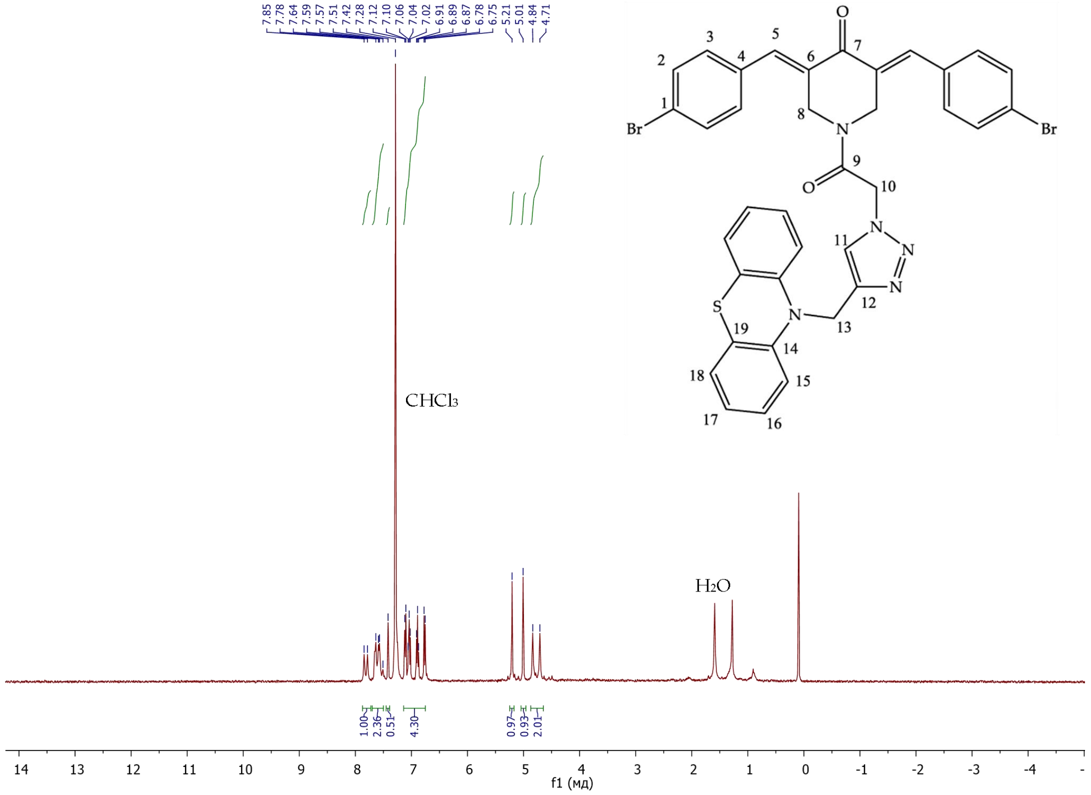

Figure S33. ^{1}H NMR spectrum of (*3E,5E*)-1-(2-(4-((10H-phenothiazine-10-yl)methyl)-1H-1,2,3-triazol-1-yl)acetyl)-3,5-bis(4-bromobenzylidene)-piperidin-4-one (**26**).

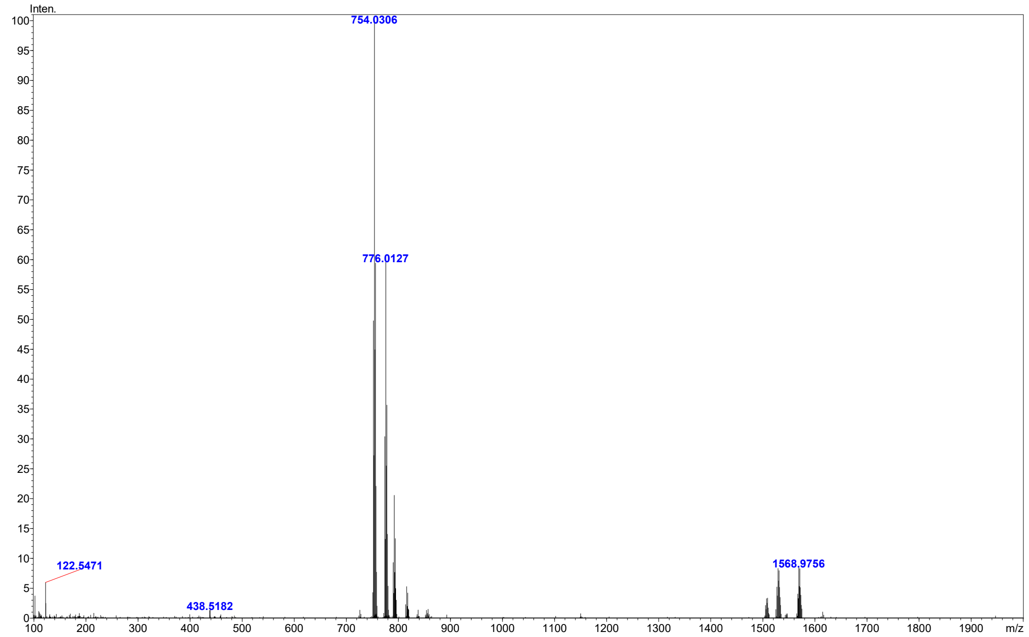

Figure S34. ESI-MS spectrum of (*3E,5E*)-1-(2-(4-((10H-phenothiazine-10-yl)methyl)-1H-1,2,3-triazol-1-yl)acetyl)-3,5-bis(4-bromobenzylidene)-piperidin-4-one (**26**).

*3.5 Figure S35-S37. Spectra of (3E,5E)-1-(2-(4-((10H-phenothiazine-10-yl)methyl)-1H-1,2,3-triazol-1-yl)acetyl)-3,5-bis(4-methoxybenzylidene)-piperidin-4-one (**27**)*

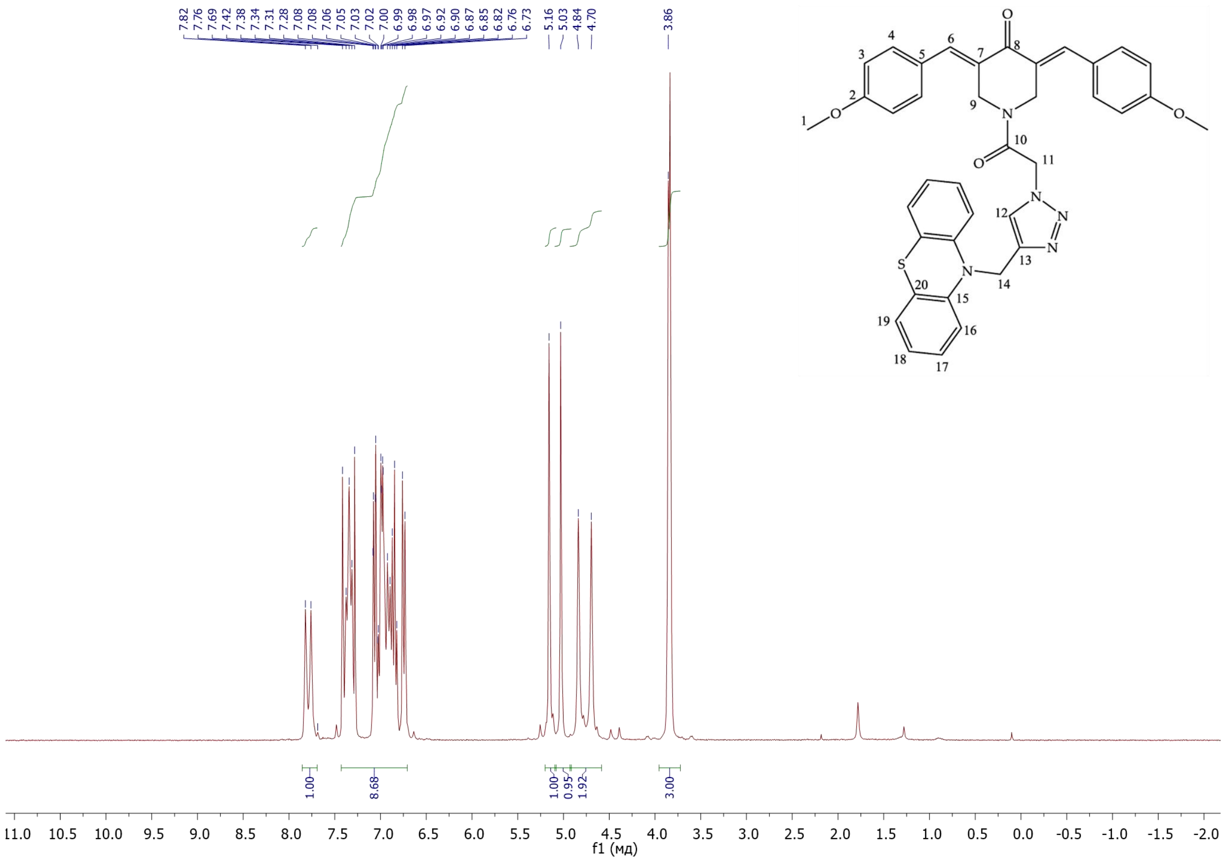

Figure S35. ^{1}H NMR spectrum of (*3E,5E*)-1-(2-(4-((10H-phenothiazine-10-yl)methyl)-1H-1,2,3-triazol-1-yl)acetyl)-3,5-bis(4-methoxybenzylidene)-piperidin-4-one (**27**).

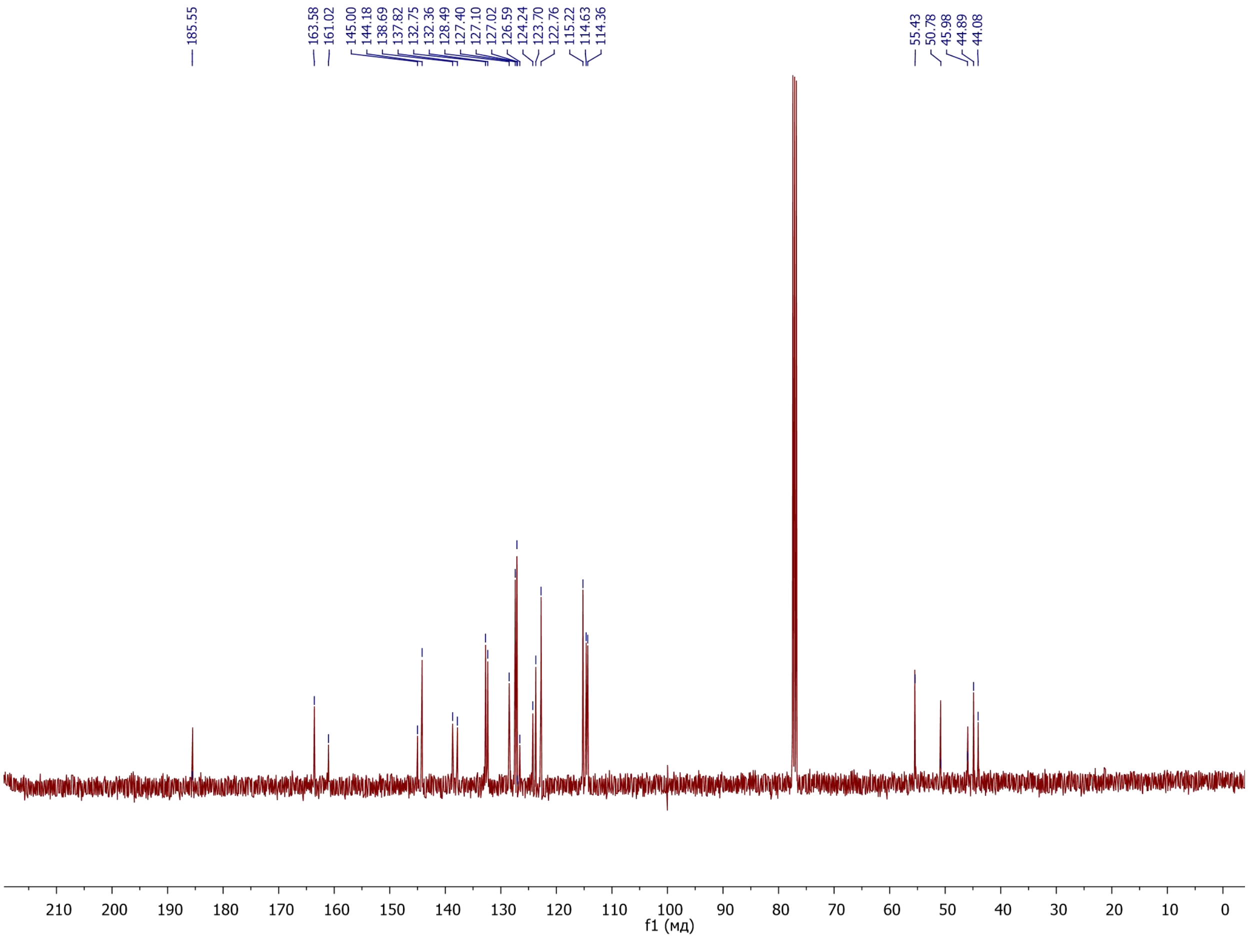

Figure S36. ^{13}C NMR spectrum of (*3E,5E*)-1-(2-(4-((10H-phenothiazine-10-yl)methyl)-1H-1,2,3-triazol-1-yl)acetyl)-3,5-bis(4-methoxybenzylidene)-piperidin-4-one (**27**).

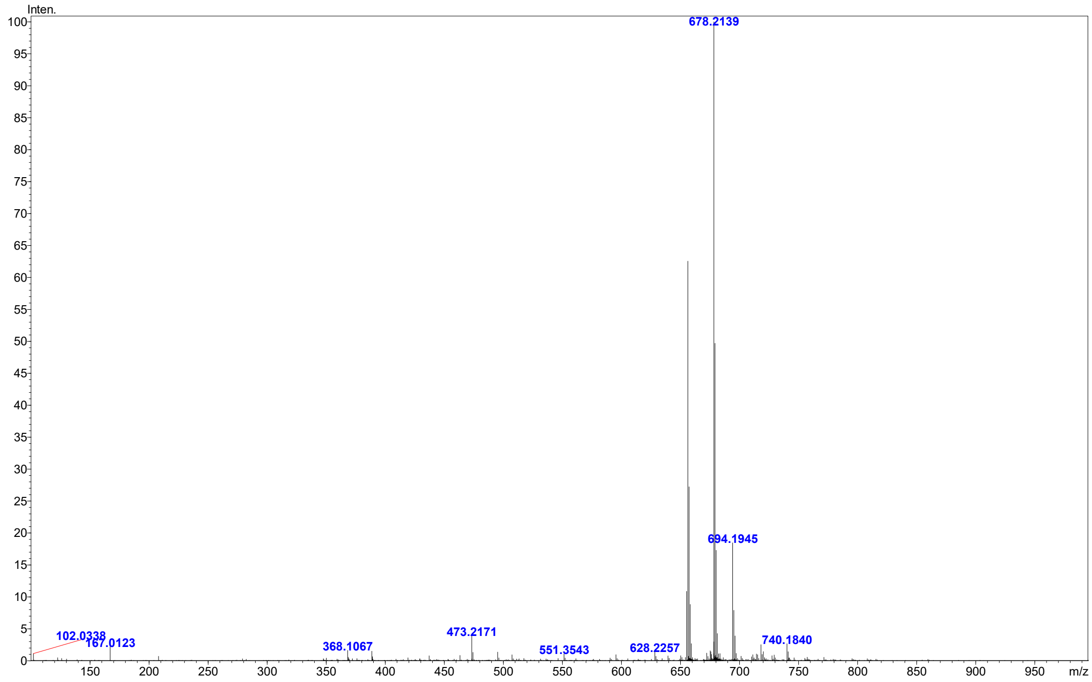

Figure S37. ESI-MS spectrum of (*3E,5E*)-1-(2-(4-((10H-phenothiazine-10-yl)methyl)-1H-1,2,3-triazol-1-yl)acetyl)-3,5-bis(4-methoxybenzylidene)-piperidin-4-one (**27**).

*3.6 Figure S38-S40. Spectra of (3E,5E)-1-(2-(4-((10H-phenothiazine-10-yl)methyl)-1H-1,2,3-triazol-1-yl)acetyl)-3,5-bis(4-isopropylbenzylidene)-piperidin-4-one (**28**)*

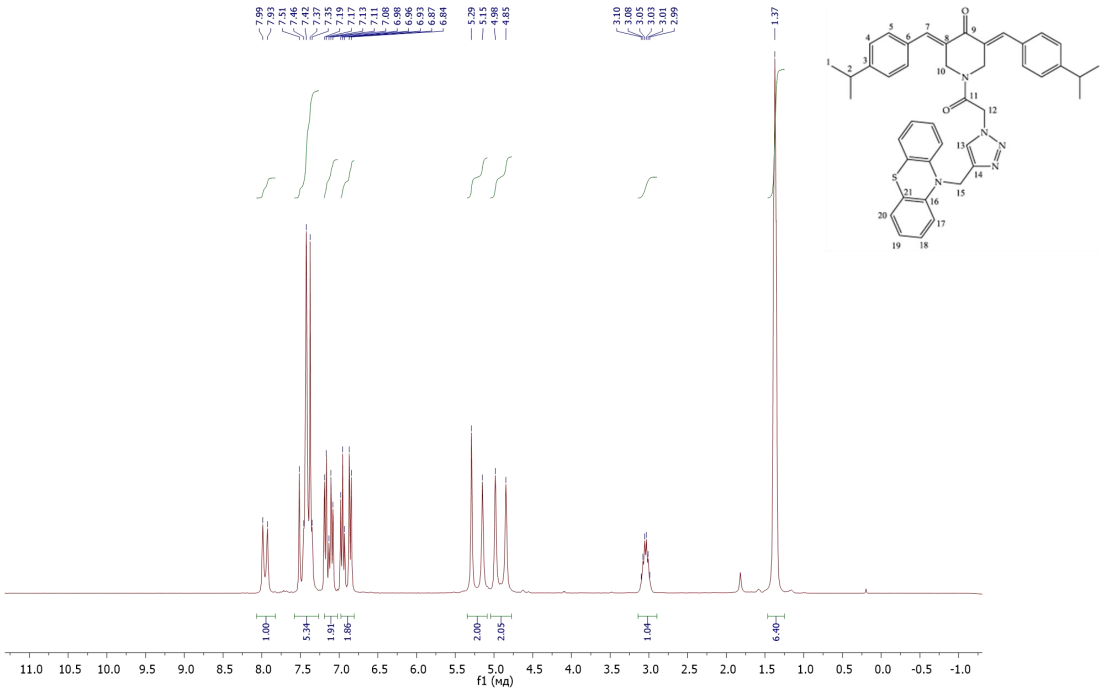

Figure S38. ^{1}H NMR spectrum of (*3E,5E*)-1-(2-(4-((10H-phenothiazine-10-yl)methyl)-1H-1,2,3-triazol-1-yl)acetyl)-3,5-bis(4-isopropylbenzylidene)-piperidin-4-one (**28**).

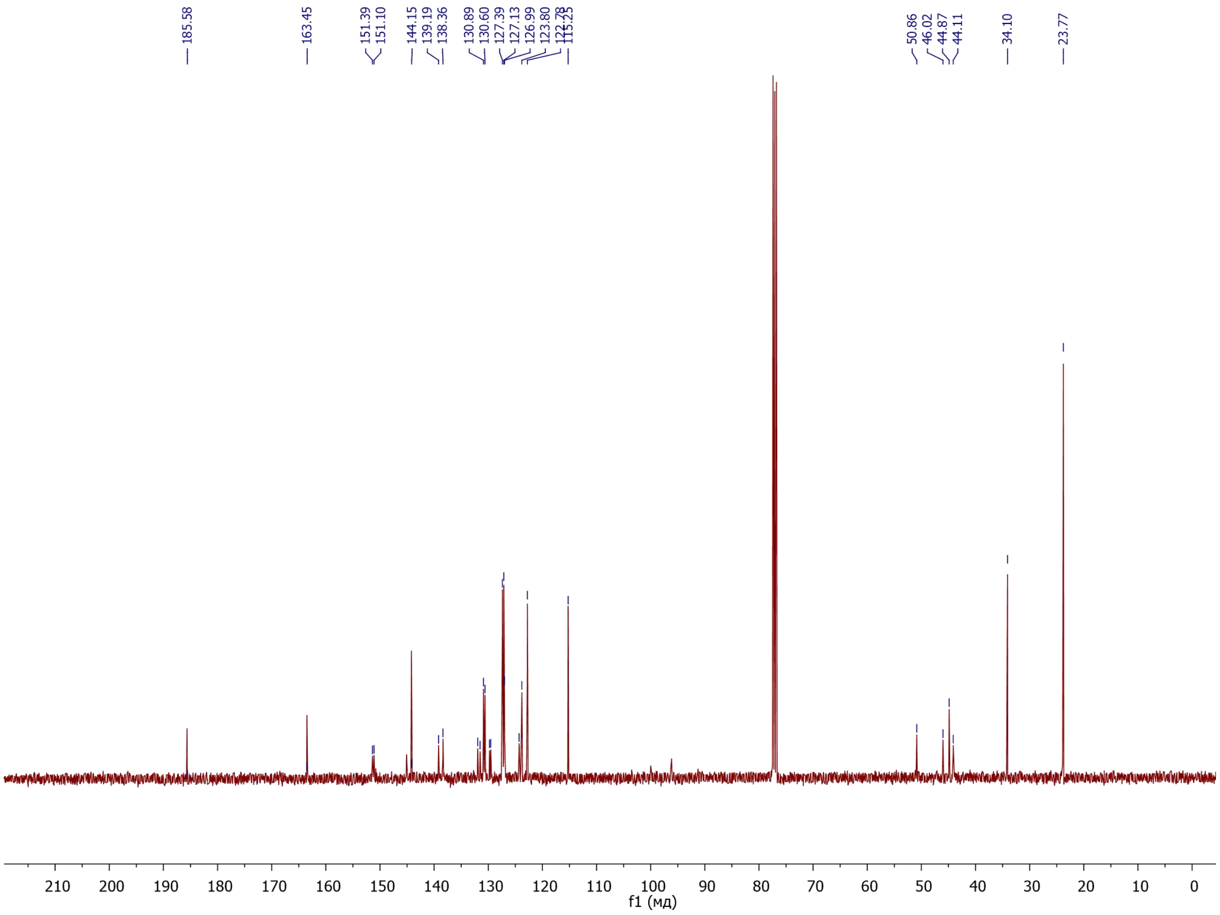

Figure S39. ^{13}C NMR spectrum of (*3E,5E*)-1-(2-(4-((10H-phenothiazin-10-yl)methyl)-1H-1,2,3-triazol-1-yl)acetyl)-3,5-bis(4-isopropylbenzylidene)-piperidin-4-one (**28**).

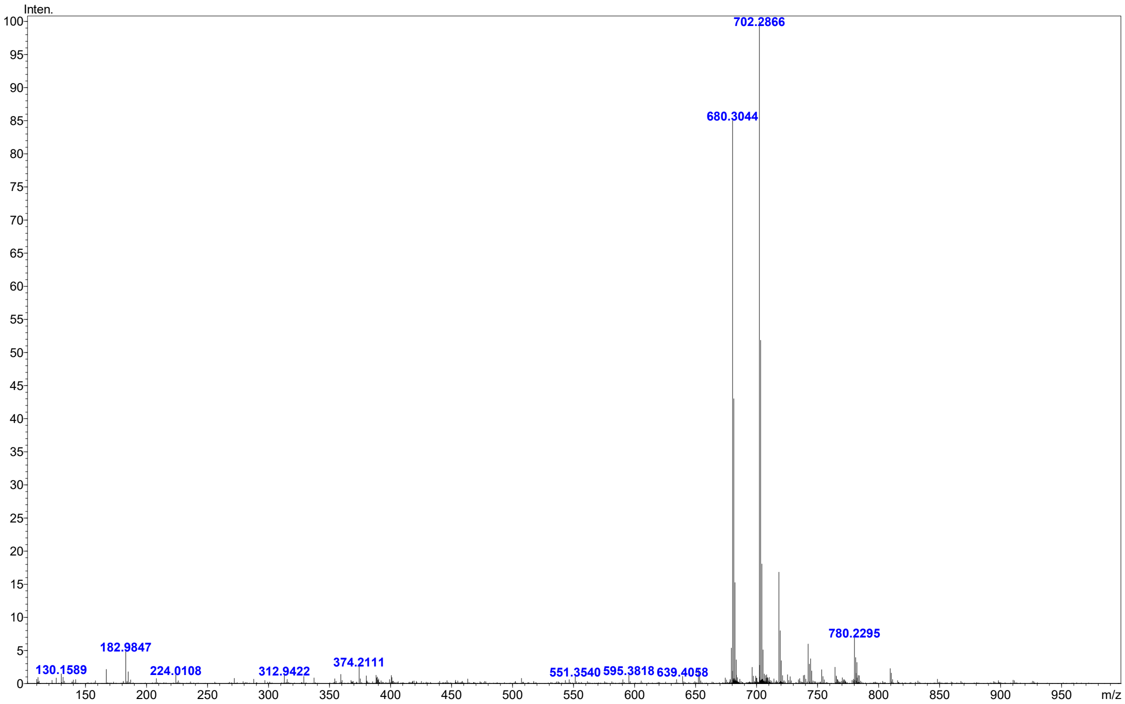

Figure S40. ESI-MS spectrum of (*3E,5E*)-1-(2-(4-((10H-phenothiazin-10-yl)methyl)-1H-1,2,3-triazol-1-yl)acetyl)-3,5-bis(4-isopropylbenzylidene)-piperidin-4-one (**28**).

*3.7 Figure S41-S43. Spectra of (3E,5E)-1-(2-(4-((10H-phenothiazine-10-yl)methyl)-1H-1,2,3-triazol-1-yl)acetyl)-3,5-bis(3,4,5-trimethoxybenzylidene)-piperidin-4-one (**29**)*

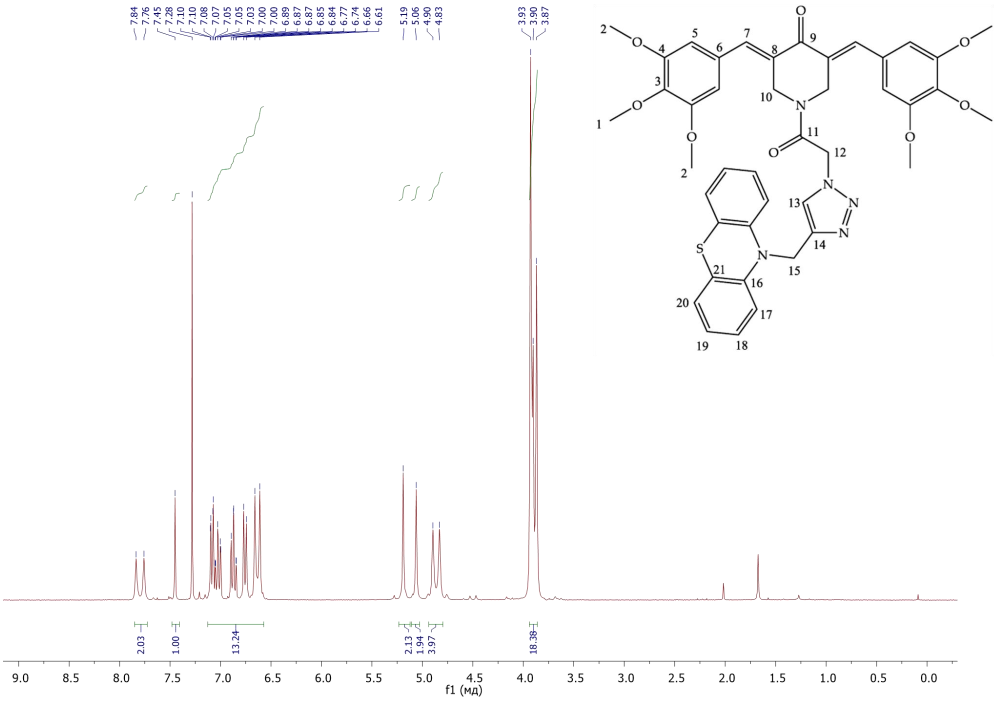

Figure S41. ^{1}H NMR spectrum of (*3E,5E*)-1-(2-(4-((10H-phenothiazine-10-yl)methyl)-1H-1,2,3-triazol-1-yl)acetyl)-3,5-bis(3,4,5-trimethoxybenzylidene)-piperidin-4-one (**29**).

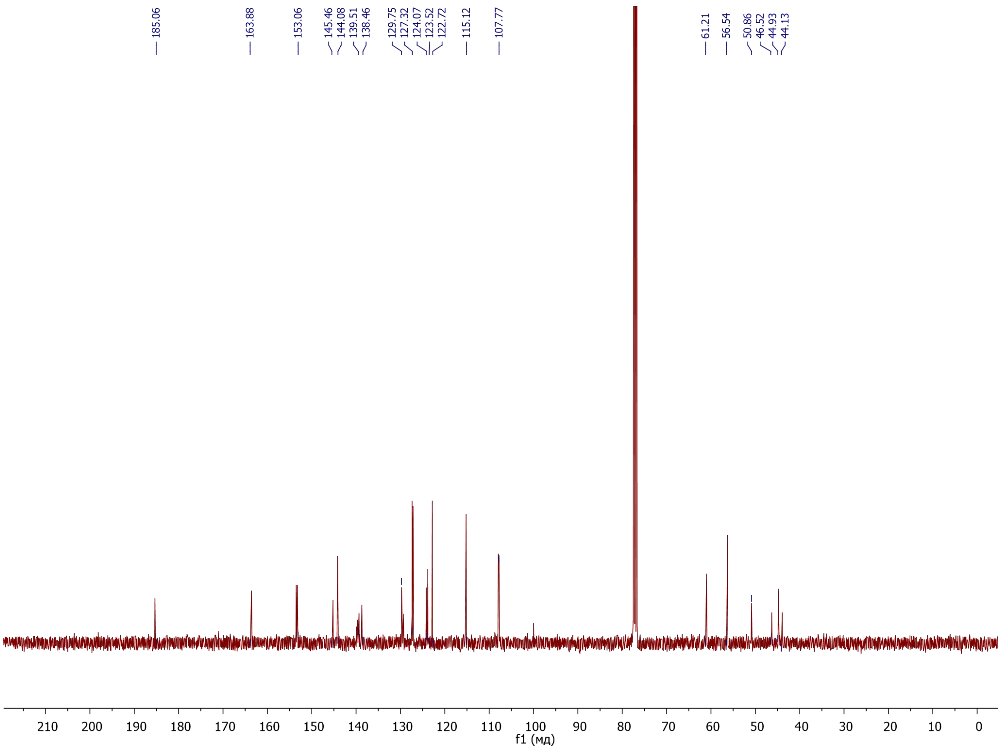

Figure S42. ^{13}C NMR spectrum of (*3E,5E*)-1-(2-(4-((10H-phenothiazine-10-yl)methyl)-1H-1,2,3-triazol-1-yl)acetyl)-3,5-bis(3,4,5-trimethoxybenzylidene)-piperidin-4-one (**29**).

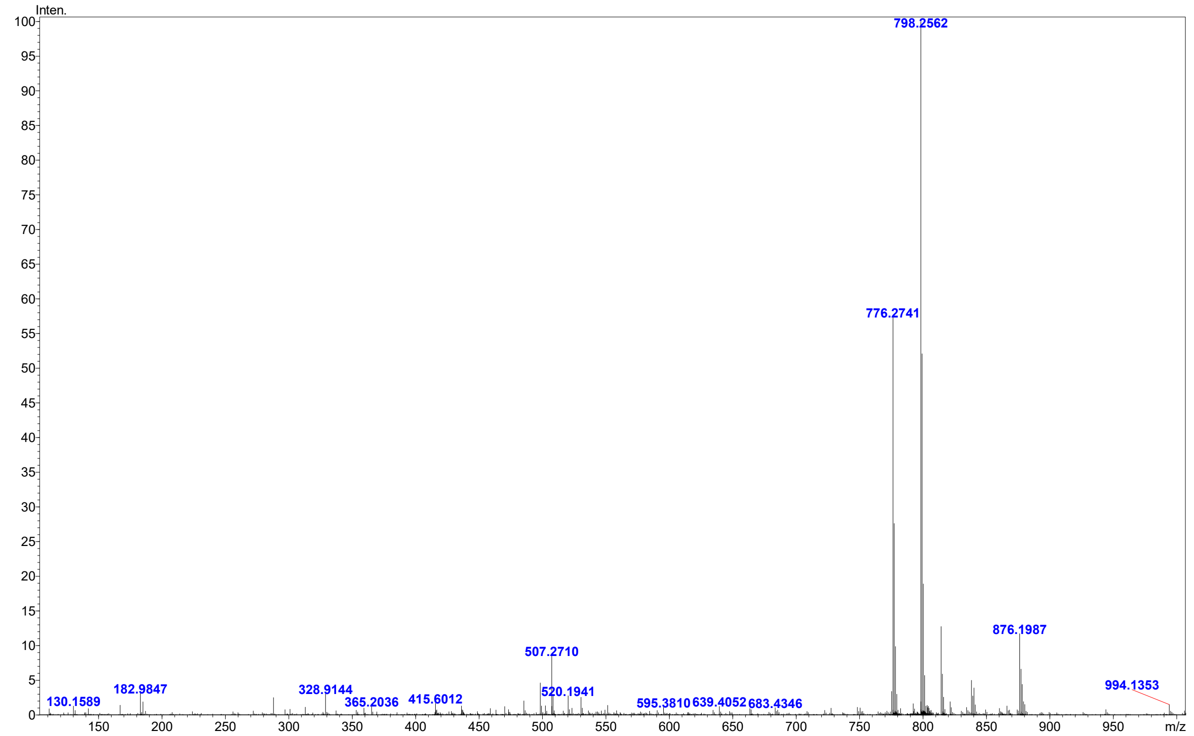

Figure S43. ESI-MS spectrum of (*3E,5E*)-1-(2-(4-((10H-phenothiazine-10-yl)methyl)-1H-1,2,3-triazol-1-yl)acetyl)-3,5-bis(3,4,5-trimethoxybenzylidene)-piperidin-4-one (**29**).

4. *Figure S44-S53. NMR spectra of (3E,5E)-1-(2-(4-((10H-phenothiazine-10-yl)methyl)-1H-1,2,3-triazol-1-yl)acetyl)-3,5-bis(benzylidene)-piperidin-4-ones hydrochloride (**30-36**)*

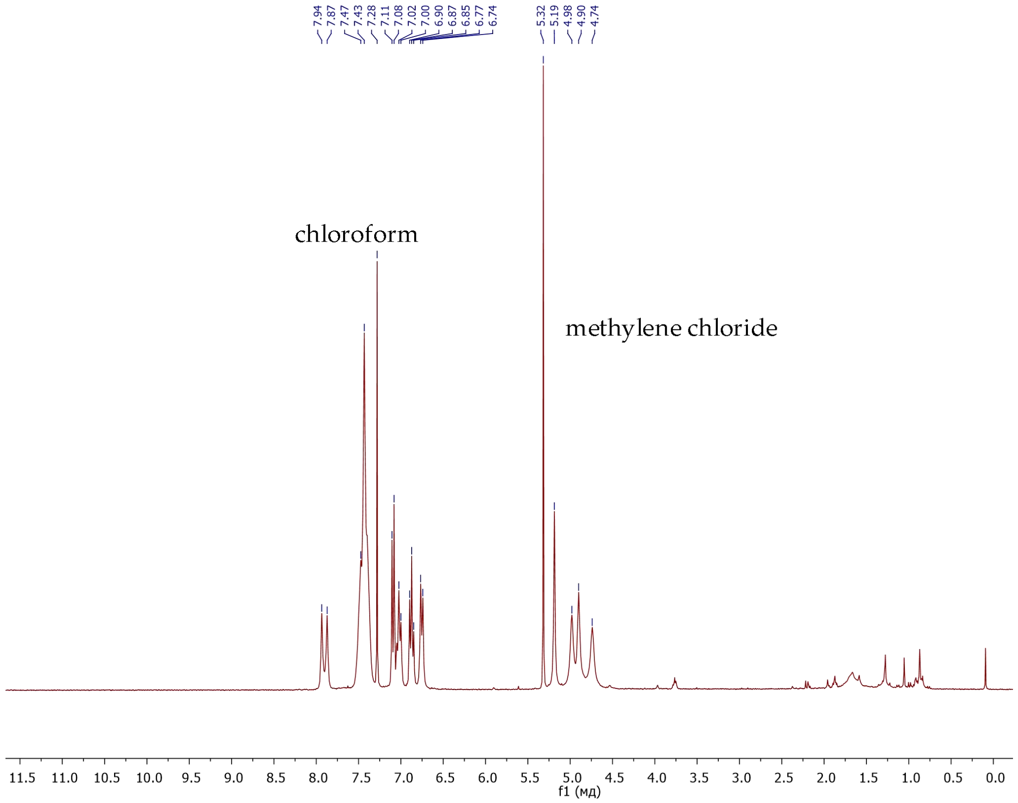

Figure S44. ^{1}H NMR spectrum of (3E,5E)-1-(2-(4-((10H-phenothiazin-10-yl)methyl)-1H-1,2,3-triazol-1-yl)acetyl)-3,5-bis(benzylidene)-piperidin-4-one hydrochloride (**30**).

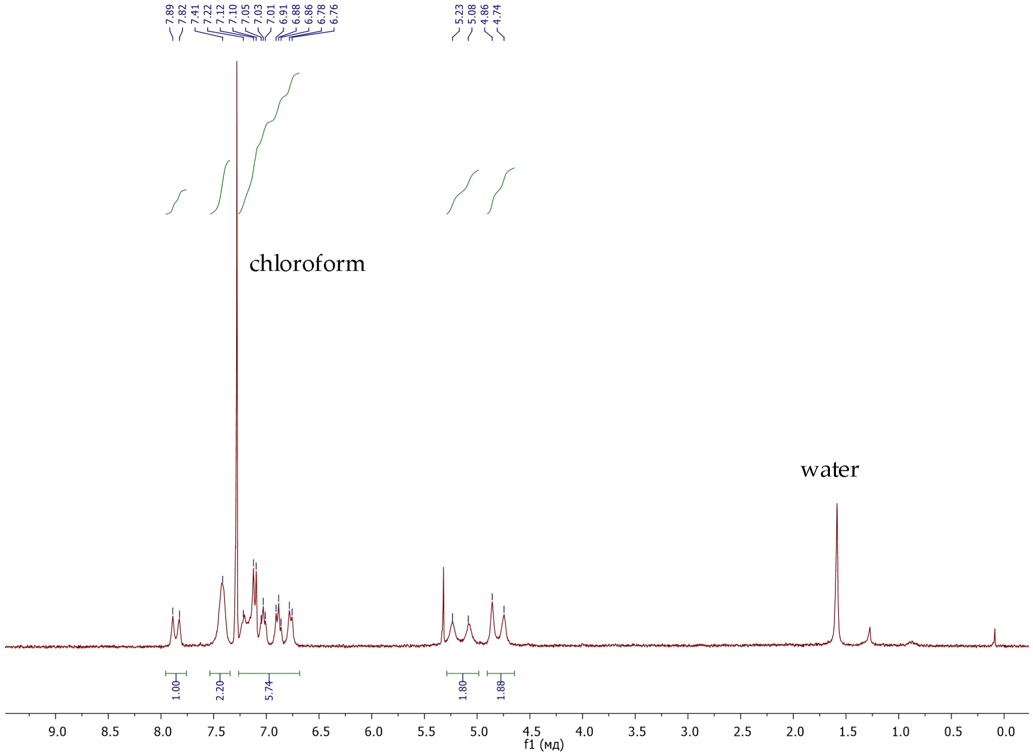

Figure S45. ^{1}H NMR spectrum of (3E,5E)-1-(2-(4-((10H-phenothiazin-10-yl)methyl)-1H-1,2,3-triazol-1-yl)acetyl)-3,5-bis(4-fluorobenzylidene)-piperidin-4-one hydrochloride (**31**).

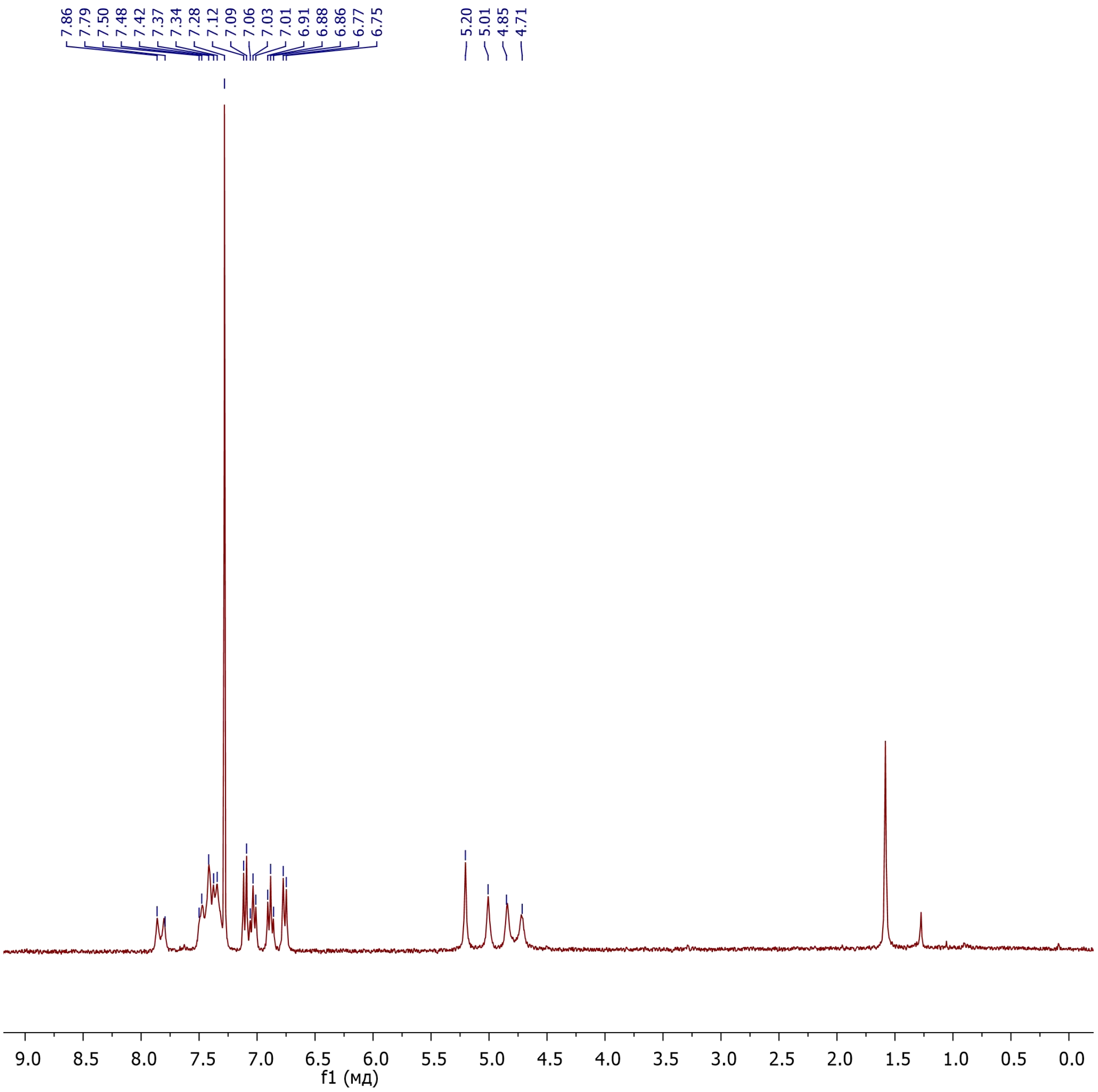

Figure S46. ^{1}H NMR spectrum of (3E,5E)-1-(2-(4-((10H-phenothiazin-10-yl)methyl)-1H-1,2,3-triazol-1-yl)acetyl)-3,5-bis(4-chlorobenzylidene)-piperidin-4-one hydrochloride (**32**).

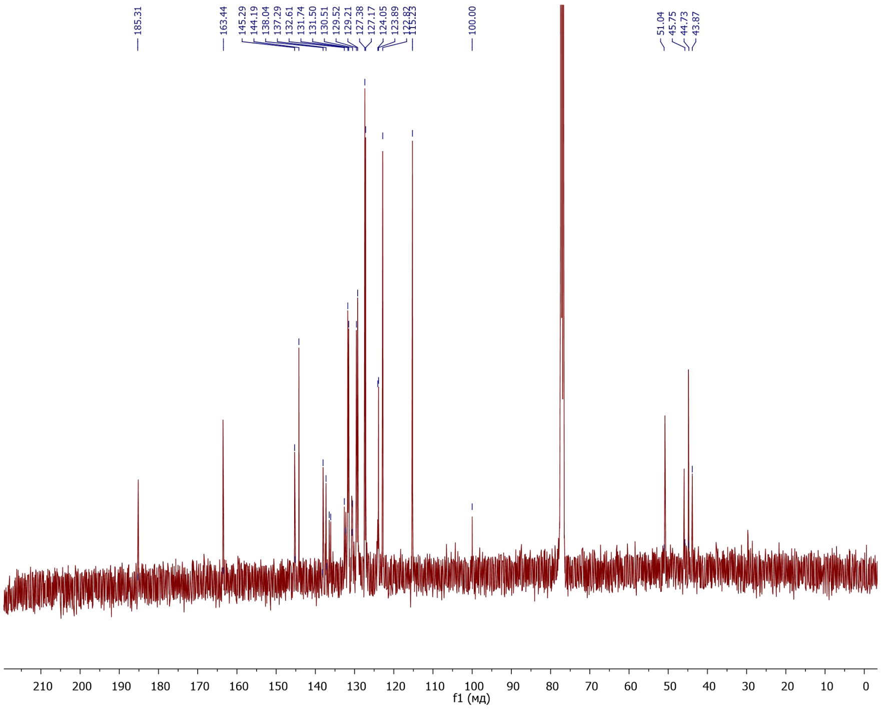

Figure S47. ^{13}C NMR spectrum of (3E,5E)-1-(2-(4-((10H-phenothiazin-10-yl)methyl)-1H-1,2,3-triazol-1-yl)acetyl)-3,5-bis(4-chlorobenzylidene)-piperidin-4-one hydrochloride (**32**).

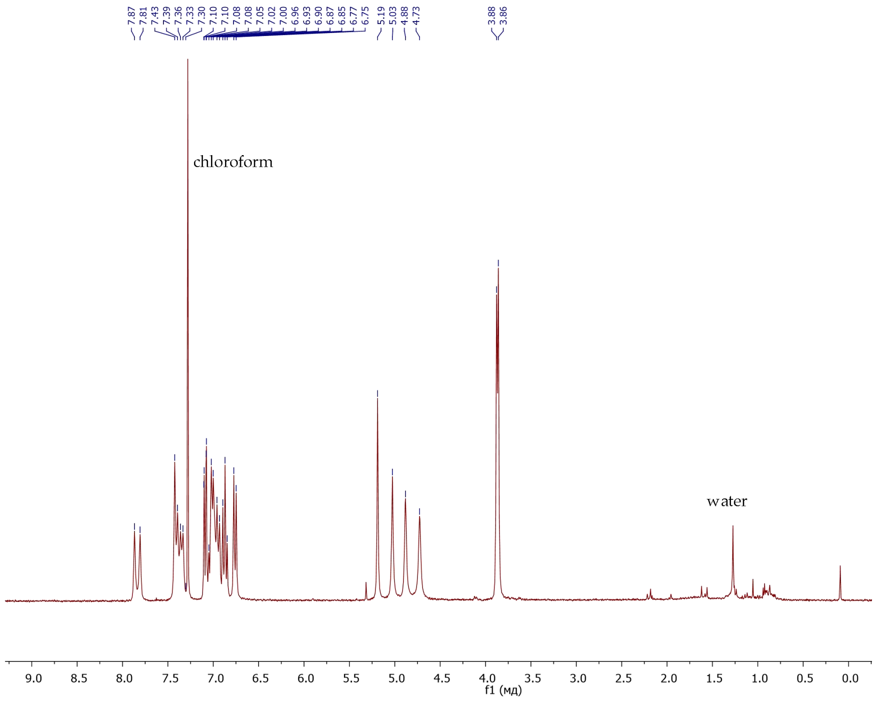

Figure S48. ^{1}H NMR spectrum of (3E,5E)-1-(2-(4-((10H-phenothiazin-10-yl)methyl)-1H-1,2,3-triazol-1-yl)acetyl)-3,5-bis(4-methoxybenzylidene)-piperidin-4-one hydrochloride (**34**).

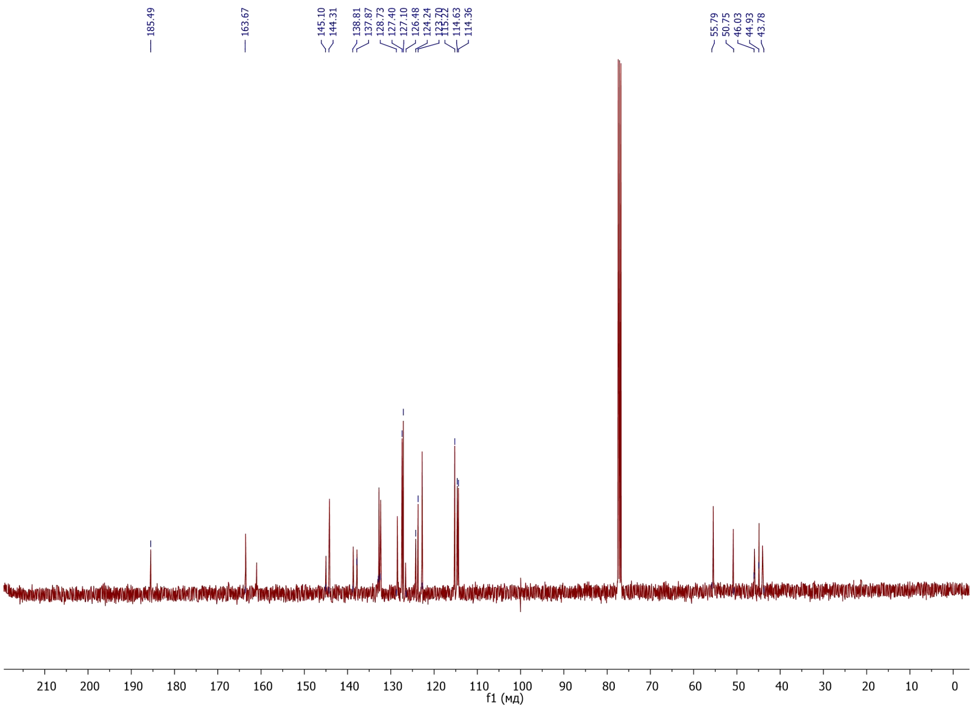

Figure S49. ^{13}C NMR spectrum of (3E,5E)-1-(2-(4-((10H-phenothiazin-10-yl)methyl)-1H-1,2,3-triazol-1-yl)acetyl)-3,5-bis(4-methoxybenzylidene)-piperidin-4-one hydrochloride (**34**)

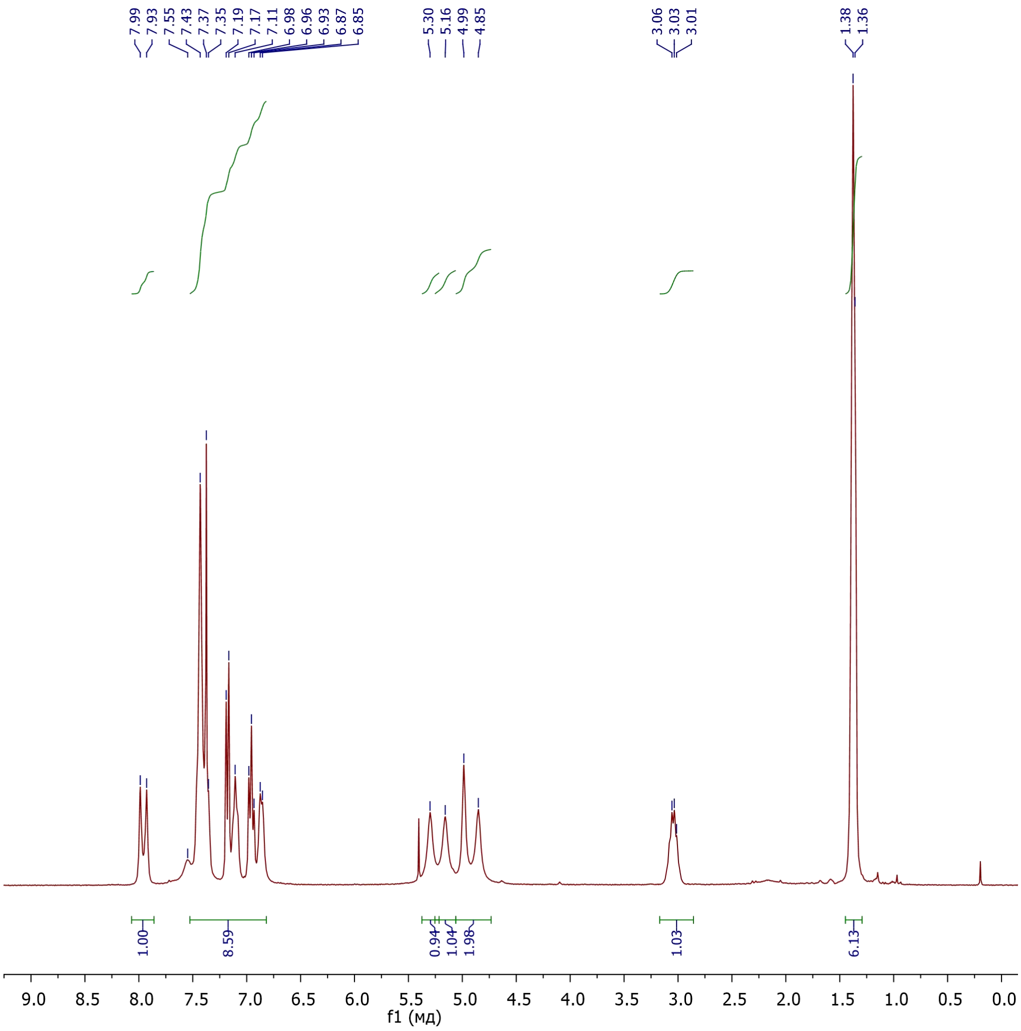

Figure S50. ^{1}H NMR spectrum of (3E,5E)-1-(2-(4-((10H-phenothiazin-10-yl)methyl)-1H-1,2,3-triazol-1-yl)acetyl)-3,5-bis(4-isopropylbenzylidene)-piperidin-4-one hydrochloride (**35**).

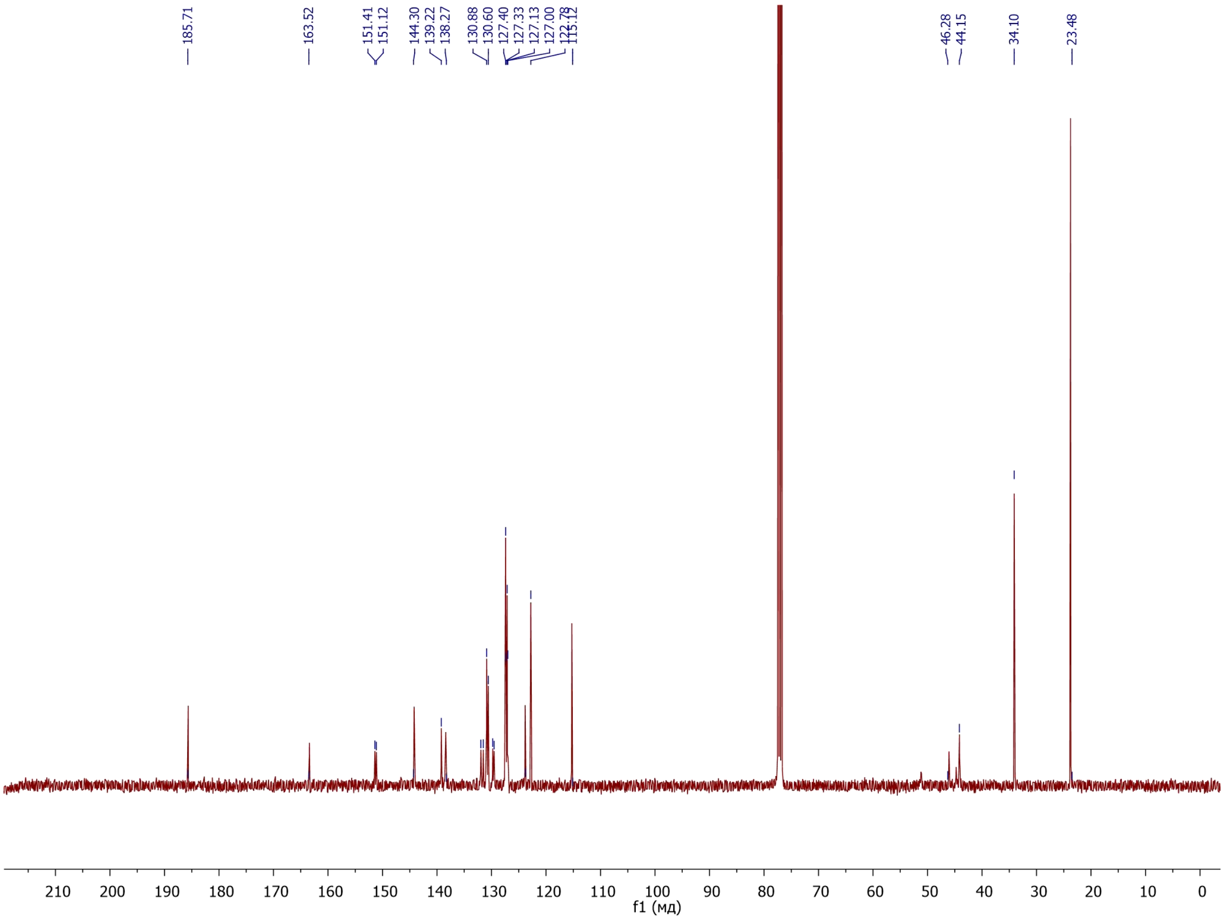

Figure S51. ^{13}C NMR spectrum of (3E,5E)-1-(2-(4-((10H-phenothiazin-10-yl)methyl)-1H-1,2,3-triazol-1-yl)acetyl)-3,5-bis(4-isopropylbenzylidene)-piperidin-4-one hydrochloride (**35**).

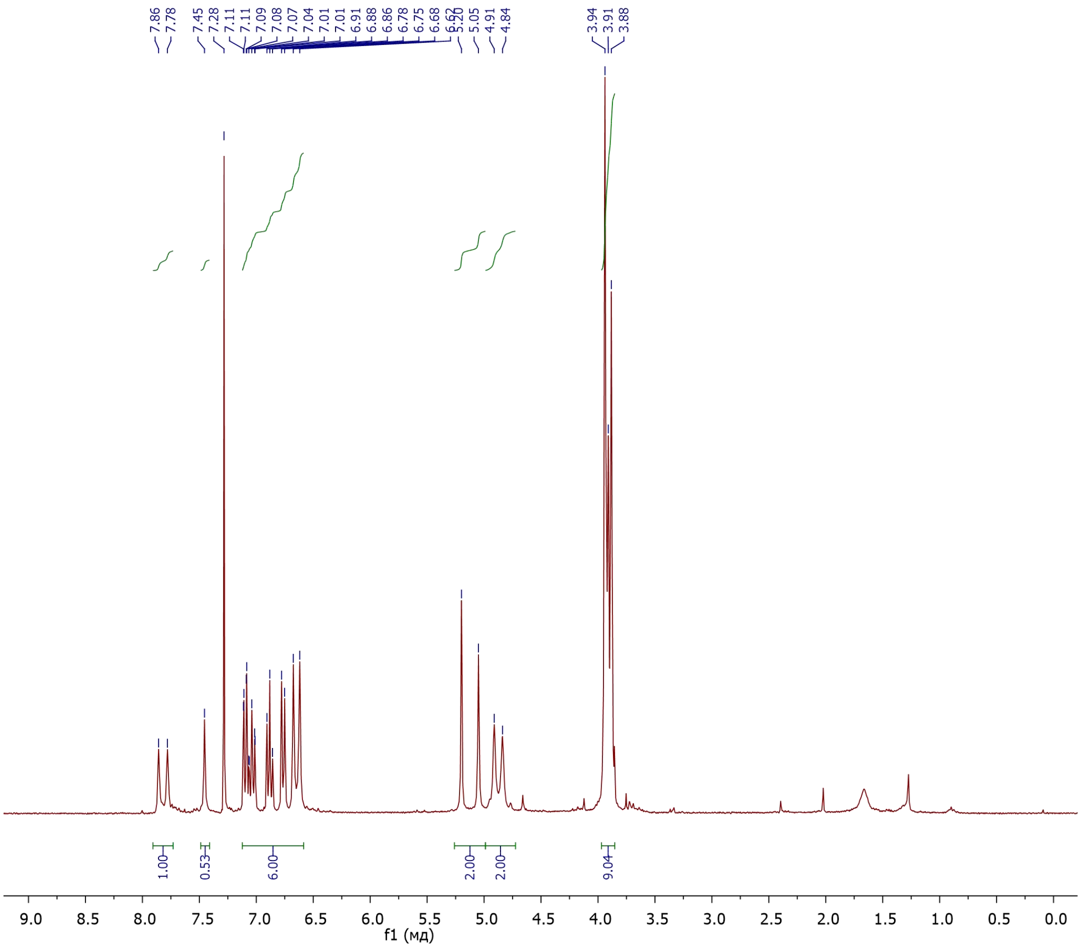

Figure S52. ^{1}H NMR spectrum of (3E,5E)-1-(2-(4-((10H-phenothiazin-10-yl)methyl)-1H-1,2,3-triazol-1-yl)acetyl)-3,5-bis(3,4,5-trimethoxybenzylidene)-piperidin-4-one hydrochloride (**36**).

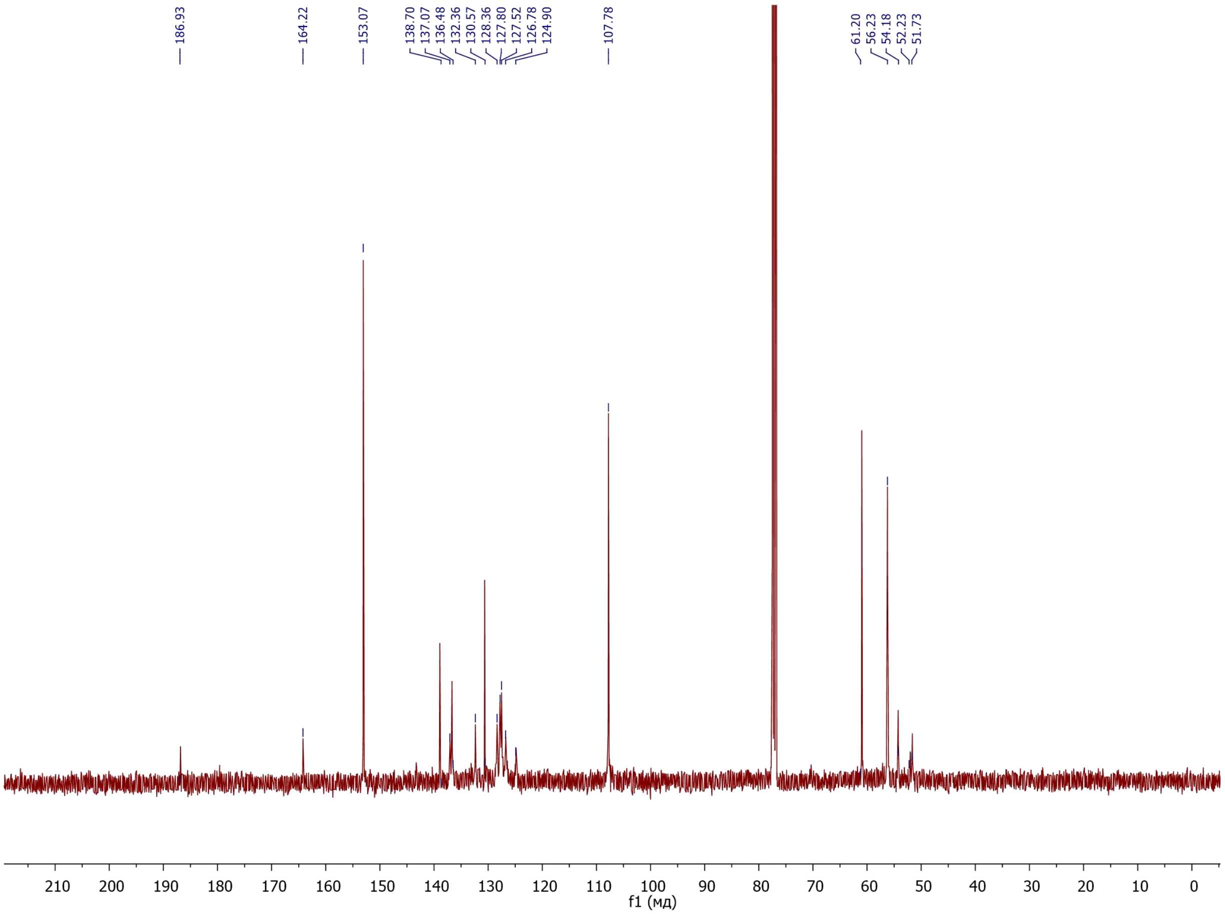

Figure S53. ^{13}C NMR spectrum of (3E,5E)-1-(2-(4-((10H-phenothiazin-10-yl)methyl)-1H-1,2,3-triazol-1-yl)acetyl)-3,5-bis(3,4,5-trimethoxybenzylidene)-piperidin-4-one hydrochloride (**36**)
